# New Verticillene Diterpenoids, Eudesmane Sesquiterpenoids, and Hydroperoxysteroids from the Further Chemical Investigation of a Taiwanese Soft Coral *Cespitularia* sp.

**DOI:** 10.3390/molecules28041521

**Published:** 2023-02-04

**Authors:** Chung-Wei Fu, You-Cheng Lin, Shu-Fen Chiou, Shu-Li Chen, Chi-Chien Lin, Hui-Chun Wang, Chang-Feng Dai, Jyh-Horng Sheu

**Affiliations:** 1 Department of Marine Biotechnology and Resources, National Sun Yat-sen University, Kaohsiung 804, Taiwan; 2 Doctoral Degree Program in Marine Biotechnology, National Sun Yat-sen University, Kaohsiung 804, Taiwan; 3 Graduate Institute of Natural Products, Kaohsiung Medical University, Kaohsiung 807, Taiwan; 4 Institute of Biomedical Sciences, National Chung Hsing University, Taichung 402, Taiwan; 5 Institute of Oceanography, National Taiwan University, Taipei 106, Taiwan; 6 Department of Medical Research, China Medical University Hospital, China Medical University, Taichung 404, Taiwan

**Keywords:** *Cespitularia* sp., verticillene diterpenoids, eudesmane sesquiterpenoids, hydroperoxysteroids

## Abstract

An investigation of the chemical composition of a Formosan soft coral *Cespitularia* sp. led to the discovery of one new verticillene-type diterpenoid, cespitulactam M (**1**); one new eudesmane sesquiterpenoid, cespilamide F (**2**); and three new hydroperoxysteroids (**3**–**5**) along with twelve known analogous metabolites (**6**–**17**). In addition, one new derivative, cespitulactam M-6,2′-diacetate (**1a**), was prepared from compound **1**. The structures were determined by detailed spectroscopic analyses, particularly HRESIMS and NMR techniques. Moreover, the in vitro cytotoxicity, anti-inflammatory, and antibacterial activity of **1**–**17** and **1a** were evaluated.

## 1. Introduction

In the past three decades, a number of studies have shown that the genus of soft coral, *Cespitularia* (a phylum of Xeniidae), elaborates diterpenoids with the verticillene skeleton [1,2,3,4,5,6,7,8,9,10,11,12,13,14,15,16,17] and eudesmane sesquiterpenoids [3,7,17] as major characteristic metabolites, mainly isolated from the species *C. hypotentaculata* and *C. taeniata*, which demonstrated cytotoxic [1,2,3,6,7,8,10,11,13], anti-inflammatory [5,16], antiviral [12], and antibacterial [11] activities. In our previous study, we investigated a series of bioactive verticillene diterpenoids as promising compounds for further marine anti-inflammatory drug development [16]. Herein, this continuous chemical investigation of the Formosan *Cespitularia* sp. collected in Green Island led to one new verticillene-type diterpenoid, one new eudesmane sesquiterpenoid, three new hydroperoxysteroids, and twelve known metabolites. Compounds **1**–**17** were evaluated for anti-inflammatory activity and cytotoxicity against human lung adenocarcinoma (A549), human hepatocellular liver carcinoma (HepG2), and human breast adenocarcinoma (MDA-MB-231) cancer cell lines, and tested for ten species of pathogenic microbes.

## 2. Results and Discussion

The extract of *Cespitularia* sp. was separated by column chromatography and HPLC to afford five new secondary metabolites (**1**–**5**) and twelve related known compounds, which were identified as 7α-hydroperoxycampesterol (**6**) [18], 7β-hydroperoxycholesterol (**7**) [19], 7α-hydroperoxycholesterol (**8**) [19], cespitulactam D (**9**) [11], cespitulactam F (**10**) [11], cespitulin S (**11**) [16], cespitularin D (**12**) [1], cespitularin O (**13**) [8], cespitulactone B (**14**) [10], cespihypotin Q (**15**) [13], atractylenolide II (**16**) [20], and atractylenolide III (**17**) [20] (Figure 1). (Supplementary materials, Figures S1–S68.)

**Figure 2:**
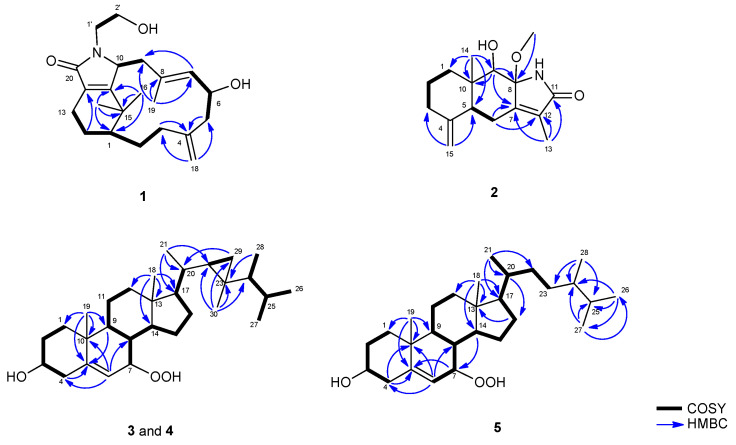
Selected ^1^H–^1^H COSY and HMBC correlations of **1**–**5**.

Cespitulactam M (**1**) was obtained as an amorphous solid and displayed HRESIMS (*m/z* 382.2350 [M + Na]^+^, calculated for C_22_H_33_NO_3_Na, 382.2353) consistent with the molecular formula C_22_H_33_NO_3_, implying seven degrees of unsaturation. The IR spectrum revealed the presence of amide (1656 cm^−1^) and hydroxy (3388 cm^−1^) groups. Subsequently, the ^1^H NMR and ^13^C NMR spectroscopic data (Table 1 and Table 2) demonstrated signals of three methyls, eight sp^3^ methylenes, one sp^2^ methylene, three sp^3^ methines, one sp^2^ methine, one sp^3^, and five sp^2^ quaternary carbons (including a carbonyl carbon appearing at *δ*_C_ 172.5 ppm). The above data accounted for four of the seven degrees of unsaturation, resulting in compound **1** with a tricyclic structure. The COSY spectrum, recorded in CDCl_3_, showed five proton sequences from H-1 to H_2_-3, H_2_-5 to H-7, H_2_-9 to H-10, H_2_-13 to H_2_-14, and H_2_-1′ to H_2_-2′. Furthermore, key HMBC correlations of H_2_-3 to C-4; H_2_-5 to C-4; H-7 to C-9; H_2_-14 to C-12; H_3_-16 to C-1, C-11, and C-15; H_3_-17 to C-1, C-11, and C-15; H_2_-18 to C-3 and C-5; H_3_-19 to C-7, C-8, and C-9 confirmed the connection of the carbon skeleton. Based on the above analysis, the planar structure of **1** was established (Figure 2).

**Figure 3:**
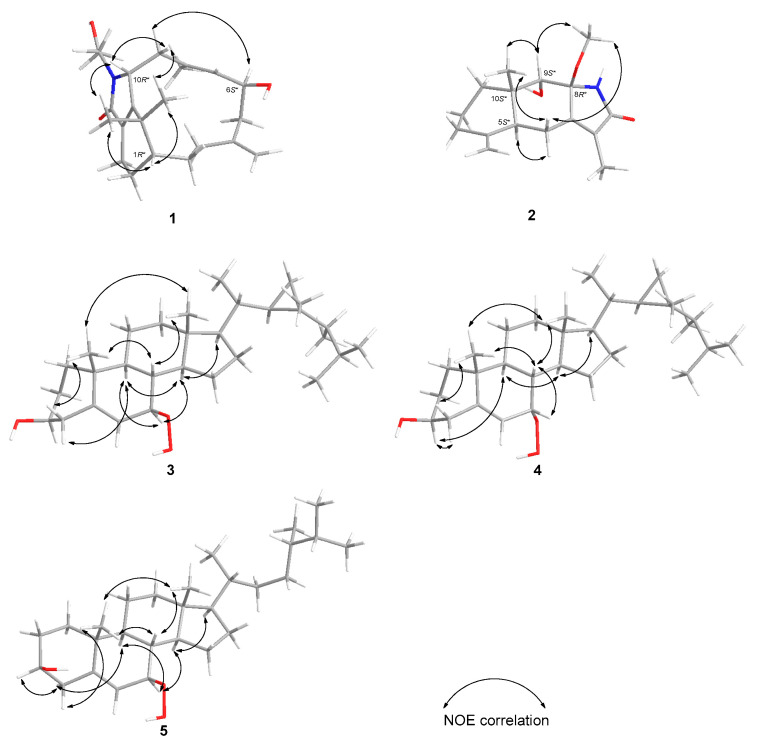
Selected NOE correlations of **1**–**5**.

With the planar structure of **1** determined, the relative stereochemistry of the three stereogenic centers, 1R*, 6S*, and 10R*, of **1,** was assigned via the analysis of the NOESY spectrum (Figure 3). It was found that H-1 displayed NOE interactions with H_3_-16 and H_3_-17, and H-10 also demonstrated NOE interactions with H_3_-16, H_3_-17, and one proton of H_2_-9 (*δ*_H_ 2.74, br d, *J* = 14.4 Hz). Based on previous studies, all naturally occurring verticillane diterpenoids are assigned H-1 as β-oriented, as shown in verticillene-type derivatives [1,2,3,4,5,6,7,8,9,10,11,12,13,14,15,16,17]. Hence, H-1, one proton of H_2_-9 (*δ*_H_ 2.74, br d, *J* = 14.4 Hz), as well as H-10, would be positioned on the β face. On the other hand, H-6 (*δ*_H_ 4.35, m) exhibited NOE correlations with the other proton of H_2_-9 (*δ*_H_ 2.68, dd, *J* = 15.0, 4.2 Hz), revealing the α-orientation of proton H-6. On the basis of the NOESY spectral analysis and MM2 force field analysis, the relative structure of cespitulactam M (**1**) was determined. The absolute configuration of **1** was suggested as 1*R*, 6*S*, and 10*R* by the proposed biosynthetic pathway as an intermediate from cespitularin C to cespitulamide C [7].

Furthermore, upon acetylation, compound **1** afforded the diacetate, cespitulactam M-6,2′-diacetate (**1a**), which exhibited two additional three-proton acetyl singlets at δ_H_ 2.01 and δ_H_ 2.04. Through the comparison of ^1^H and ^13^C NMR spectra of **1** and **1a**, the deshielding of H_2_-2′ from δ_H_ 3.85 to 4.31 and 4.12 suggested the location of an acetyl group at C-2′, while the deshielding of H-6 from δ_H_ 4.35 to 5.29 also indicated the location of an acetyl group at C-6. In addition, the HRESIMS of **1a** revealed a molecular ion at *m/z* 466.2565, [M + Na]^+^ (calculated for C_22_H_33_NO_3_Na, 466.2564).

Cespilamide F (**2**) was obtained as a white powder. The HRESIMS (*m/z* 300.1572 [M + Na]^+^, calculated for C_16_H_23_NO_3_Na, 300.1570) of **2** established the molecular formula C_16_H_23_NO_3_, appropriate for six degrees of unsaturation. Its IR spectrum also revealed the presence of amide (1693 cm^−1^) and hydroxy (3418 cm^−1^) groups. The ^13^C NMR and HSQC spectroscopic data (Table 1 and Table 2) illustrated signals of three methyls (including one methoxyl group appearing at *δ*_H_ 3.09 and *δ*_C_ 49.6 ppm), four sp^3^ methylenes, one sp^2^ methylene, two sp^3^ methines, two sp^3^, and four sp^2^ quaternary carbons (including one carbonyl carbon appearing at *δ*_C_ 174.3). The above data accounted for three of the six degrees of unsaturation, indicating a tricyclic structure for **2**. From the COSY spectrum measured in CDCl_3_, we established two proton sequences from H_2_-1 to H_2_-3 and H-5 to H_2_-6. The key HMBC correlations of H_2_-6 to C-7 and C-8; H-9 to C-7, C-8, and C-10; H_2_-13 to C-7, C-11, and C-12; H_3_-14 to C-1, C-5, C-9, and C-10; H_3_-15 to C-3 and C-5; and H_3_-16 to C-8 permitted the connection of the carbon skeleton (supplementary materials, S12–S20). Based on the above analysis, the planar structure of **2** was established (Figure 2).

The relative configuration of **2** was determined on the basis of the NOESY experiment and compared with the published compound, taenialactams A, which was isolated from *C. taeniata* in 2009 [3]. Assuming that H-5 (*δ*_H_ 2.28, d, *J* = 12.0 Hz) possesses an α-orientation similar to that of taenialactams A, it was found that H-5 (*δ*_H_ 2.28, d, *J* = 12.0 Hz) demonstrated NOE interactions with one proton of H_2_-6 (*δ*_H_ 2.54, dd, *J* = 13.2, 3.0 Hz); therefore, H-5, and one proton of H_2_-6 (*δ*_H_ 2.54, dd, *J* = 13.2, 3.0 Hz) should also be positioned on the α face. On the contrary, the other one proton of H_2_-6 (*δ*_H_ 2.16, m) exhibited NOE correlations with H_3_-14 (*δ*_H_ 1.00, s) and H_3_-8-OMe (*δ*_H_ 3.09, s). Moreover, H-9 (*δ*_H_ 3.51, s) also showed NOE correlations with H_3_-14 (*δ*_H_ 1.00, s) and H_3_-8-OMe (*δ*_H_ 3.09, s), revealing the β-orientation of H_2_-6β (*δ*_H_ 2.16, m), H_3_-8-OMe (*δ*_H_ 3.09, s), H-9 (*δ*_H_ 3.51, s), and H_3_-14 (*δ*_H_ 1.00, s). On the basis of the NOESY spectral analysis and MM2 force field analysis, the relative structure of **2** was determined as 5S*, 8R*, 9S*, and 10S* (Figure 3).

7*β*-Hydroperoxygorgosterol (**3**) was also obtained as a white powder. The HRESIMS (*m/z* 481.3651 [M + Na]^+^) of **3** confirmed the molecular formula C_30_H_50_O_3_, implying 6 degrees of unsaturation. The presence of the hydroxy (3380 cm^−1^) group was shown on the IR spectrum. The 1D ^13^C NMR and DEPT spectroscopic data (Table 1 and Table 2) showed signals of seven methyls, eight sp^3^ methylenes, ten sp^3^ methines, one sp^2^ methine, three sp^3^, and one sp^2^ quaternary carbons. The above data accounted for one of the six degrees of unsaturation, indicating a pentacyclic structure for **3** (supplementary materials, S21–S28). From the COSY spectrum measured in CDCl_3_, it was possible to establish five proton sequences from H_2_-1 to H_2_-4, H-7 to H_2_-11, H-14 to H-17, H-17 to H-20, H-22 to H_2_-29, and H-24 to H_3_-28. Key HMBC correlations of H_2_-4 to C-5; H-6 to C-4, C-8, and C-10; H-9 to C-5; H_3_-18 to C-12, C-13, C-14, and C-17; H_3_-19 to C-1, C-5, C-9, and C-10; H_3_-21 to C-17, C-20, and C-22; H_3_-28 to C-23; and H_3_-30 to C-22, C-23, C-24, and C-29 permitted the connection of the carbon skeleton. On the basis of the above analysis, the planar structure of **3** was established (Figure 2).

The relative configuration of **3** was elucidated on the basis of the observed key NOE correlations (Figure 3). In particular, the stereo center of C-7 was the most significant result in this compound; thus, it was compared with the reported compounds 7*β*-hydroperoxycholesterol and its stereoisomer 7*α*-hydroperoxycholesterol [19]. In terms of the absolute configuration of the side chain, the 1D NMR spectra of compound **3** were compared with those of gorgosterol [21] and 7-oxogorgosterol [22] from previous research. It turned out that the chemical shifts of compound **3** were similar to that of 7-oxogorgosterol as 20*R*, 22*R*, 23*R*, and 24*R*. Thus, the absolute configuration of 7*β*-hydroperoxygorgosterol (**3**) was proposed.

7*α*-Hydroperoxygorgosterol (**4**) was isolated as a white powder. The HRESIMS exhibited a [M + Na]^+^ ion peak at 481.3652 *m/z*, establishing a molecular formula of C_30_H_50_O_3_. By 2D NMR spectroscopy data, including HSQC, COSY, and HMBC (supplementary materials, S29–S36), compound **4** was displayed to possess the same molecular framework as that of **3**. The molecular formula of **3** and **4** indicates that **4** is an isomer of **3**. On the basis of the above references and its NOE correlations, compound **4** was revealed to be the C-7 epimer of **3**, namely 7*α*-hydroperoxygorgosterol (**4**).

The HRESIMS of 7*β*-hydroperoxycampesterol (**5**) showed that it possesses the molecular formula C_28_H_48_O_3_ (*m/z* 455.3495 [M + Na]^+^). The IR spectrum of **5** showed the absorption of a hydroxy group (3383 cm^−1^). Comparison of the ^1^H and ^13^C NMR spectroscopic data of compound **5** with known compound 7*α*-hydroperoxycampesterol (**6**) suggested that the planar structure of **5** was the same as 7*α*-hydroperoxycampesterol [18].

Owing to the NMR data of **5** and the reported compound 7*α*-hydroperoxycampesterol (**6**), this suggests that **5** is an isomer of **6**. Compound **5** was also compared with the known compounds 7*β*-hydroperoxycholesterol and 7*α*-hydroperoxycholesterol in order to define the stereo center C-7 [19]. In terms of the chiral center C-24 on the side chain, the 1D NMR data were compared with those of (24*R*)-campesterol and (24*S*)-methylcholesterol [23,24]. Based on the previous literature and NOE correlations, the absolute configuration of **5** was proposed to be 3*S*, 7*R*, and 24*S*.

Compounds **1**–**16** and **1a** were also evaluated for their cytotoxicity to human lung adenocarcinoma (A549), human hepatocellular carcinoma (HepG2), and human breast adenocarcinoma (MDA-MB-231) cancer cell lines by using the Almar Blue assay [25,26]. The results showed that only 7*β*-hydroperoxycampesterol (**5**) exhibited cytotoxicity (IC_50_ = 15.40, 18.74 μg/mL) toward the cell lines MDA-MB-231 and A549, compared with the positive control, doxorubicin (IC_50_ 0.30, 0.15 μg/mL), respectively, while others did not exhibit cytotoxicity within 20 μg/mL.

Furthermore, the antibacterial activities of **3**–**7**, **9**, and **12** were tested against the growth of a limited panel of bacteria strains, including *Bacillus subtilis*, *Enterobacter aerogenes*, *Escherichia coli*, *Klebsiella pneumoniae*, *Salmonella enteritidis*, *Salmonella typhimurium*, *Serratia marcescens*, *Shigella sonnei*, *Staphylococcus aureus*, and *Yersinia enterocolitica*. As a result, compound **5** exhibited antibacterial activities against *S. enteritidis* (inhibition zone: 6.0 mm) and *K. pneumoniae* (inhibition zone: 5.0 mm), and compound **9** showed an inhibition zone of 9.0 mm against *K. pneumoniae* at the dosage of 25 μg/disk by the disc diffusion method, compared with the positive control, ampicillin, against *S. enteritidis* (inhibition zone: 10.0 mm) and *K. pneumoniae* (inhibition zone: 5.0 mm) at the same dosage of 25 μg/disk, while others did not show activities to these bacteria strains.

In order to discover bioactive compounds with anti-inflammatory activities by inhibiting TNF-α, PGE_2_, and NO overproduction, **1**–**10**, **1a**, **14**, and **15** isolated from this extract were assayed as previously described [16]. At a concentration of 100 µM, cespihypotin Q (**15**) could weakly inhibit TNF-α expression and PGE_2_ by 23.6 ± 2.5% and 21.2 ± 0.9%, respectively, relative to the control cells treated with LPS only. In addition, compounds **3**, **4**, **14**, and **15** inhibited NO release by 33.8 ± 1.5, 34.9 ± 3.9, 24.8 ± 1.4, and 35.0 ± 3.7%, respectively, at a concentration of 100 µM for compounds **3**, **14**, and **15**, and at 25 µM for compound **4** (supplementary materials, Tables S1–S3).

## 3. Materials and Methods

### 3.1. General Experimental Procedures

Optical rotations were measured on a JASCO P-1020 digital polarimeter (Jasco Corporation, Tokyo, Japan). IR spectra were recorded on a JASCO P-1020 FT-IR-4100 (Jasco Corporation, Tokyo, Japan) and Nicolet iS5 FT-IR infrared spectrophotometers (Thermo Fisher Scientific Inc., Waltham, MA, USA). The NMR spectra were recorded on a JEOL ECZ600R FT-NMR (JEOL Ltd., Tokyo, Japan) at 600 and 150 MHz for ^1^H and ^13^C, respectively, or on a Varian Unity Inova 500 FT-NMR (Varian Inc., Palo Alto, CA, USA) at 500 and 125 MHz for ^1^H and ^13^C, respectively, or on a Varian 400 FT-NMR at 400 and 100 MHz for ^1^H and ^13^C, respectively. All NMR experiments were performed at room temperature using CDCl_3_ as solvent. ESIMS and HRESIMS data were obtained with a Bruker APEX II mass spectrometer (Bruker, Bremen, Germany). Silica gel (200–400 mesh, Merck, Darmstadt, Germany), reversed-phase silica gel (C18; 230–400 mesh, Merck, Darmstadt, Germany), or Sephadex LH-20 gel (particle size: 18–111 μg, GE Healthcare, Chicago, IL, USA) were used for column chromatography (C.C.). Precoated silica gel plates (Kieselgel 60 F254, 0.2 mm, Merck, Darmstadt, Germany) were also used for analytical thin-layer chromatography (TLC). HPLC was performed on a Hitachi diode array detector L-2455 system and a pump L-2130 system equipped with a Supelco C18 column (5 µm, 250 × 21.2 mm; Merck, Darmstadt, Germany).

### 3.2. Animal Material

The soft coral, *Cespitularia* sp., was collected by hand using SCUBA from the coast of Green Island, Taiwan, in June 2007, at a depth of 10–15 m and stored in a –20 °C freezer until extraction. The soft coral was identified by Professor Chang-Feng Dai, Institute of Oceanography, National Taiwan University. A voucher sample was deposited at the Department of Marine Biotechnology and Resources, National Sun Yat-sen University.

### 3.3. Extraction and Isolation

The frozen bodies of soft coral *Cespitularia* sp. (880 g, wet weight) were minced and extracted with EtOAc (1 L × 5) and further extracted exhaustively with MeOH (1 L × 5). Afterward, the EtOAc extract (4.26 g) was chromatographed by silica gel open column chromatography with solution EtOAc in *n*-hexane (0–100%, gradient) and then substituted for MeOH in EtOAc (0–100%, gradient) to yield 14 fractions. Fraction 6 was further separated via *n*-hexane/EtOAc (6:1–2:1, gradient) to afford seven subfractions (6-1–6-7). In the next step, subfraction 6-7 was purified by reversed-phase HPLC with MeOH/H_2_O (19:1) to afford six hydroperoxysterols; that is, novel chemical structures **3** (2.2 mg), **4** (1.2 mg), **5** (2.6 mg), and known compounds **6** (1.7 mg), **7** (1.8 mg) and **8** (0.7 mg). In terms of known compound **16** (1.9 mg), this compound was isolated from subfraction 6-2 via reversed-phase HPLC with ACN/H_2_O (7:4).

On the other hand, the MeOH extract of this soft coral, *Cespitularia* sp., was partitioned by CH_2_Cl_2_ and H_2_O in order to separate the CH_2_Cl_2_ soluble fraction for further study. Initially, the CH_2_Cl_2_ extract (3.83 g) was chromatographed by silica gel open column chromatography and eluted with EtOAc in *n*-hexane (0–100%, gradient), and then replaced by acetone, MeOH in EtOAc (0–100%, gradient) to yield 17 fractions. Subsequently, fraction 7 was eluted with *n*-hexane/EtOAc (6:1–3:1, gradient) so as to afford five subfractions (7-1–7-5). Afterward, subfraction 7-3 was separated with *n*-hexane/EtOAc (5:1) and further purified by reversed-phase HPLC with MeOH/H_2_O (2:1) to obtain known compounds **15** (1.7 mg) and **17** (11.6 mg). Similarly, subfraction 7-4 was separated with *n*-hexane/EtOAc (4:1) and eluted via reversed-phase HPLC with ACN/H_2_O (1:1) to obtain known verticillene diterpenoids **11** (1.5 mg) and **14** (1.2 mg). In the next fraction (Fr. 8), this sample was eluted by Sephadex LH-20 column with MeOH, which belongs to size-exclusion chromatography, to yield 6 fractions. Afterward, subfraction 8-5 was purified by RP-HPLC with ACN/H_2_O (1:1) in an effort to obtain a new sesquiterpenoid **2** (1.0 mg) as well as two known verticillene diterpenoids **12** (2.2 mg) and **13** (0.9 mg). Subsequently, a similar method to that used for fraction 8 was used for fractions 14 and 15, except for different solvent systems in RP-HPLC at the last step. To be more specific, known cespitulactam **9** (2.7 mg) and **10** (1.6 mg) were isolated from fraction 14 by reversed-phase HPLC with MeOH/H_2_O (3:2); a new cespitulactam **1** (2.8 mg) was eluted with ACN/H_2_O (1:2) from fraction 15.

*Cespitulactam M* (**1**): Amorphous powder; [α]^25^_D_ −165 (*c* 0.05, CHCl_3_); UV (MeOH) *λ*_max_ (log *ε*) 241 (3.5) nm; IR (neat) *v*_max_ 3388, 2921, 2362, 1656 and 1450 cm^−1^; ^1^H (600 MHz, CDCl_3_) (see Table 1) and ^13^C NMR (150 MHz, CDCl_3_) data (see Table 2); HRESIMS *m/z* 382.2350 [M + Na]^+^ (calcd for C_22_H_33_NO_3_Na, 382.2353).

*Cespilamide F* (**2**): Amorphous powder; [α]^25^_D_ +40 (*c* 0.04, CHCl_3_); UV (MeOH) *λ*_max_ (log *ε*) 230 (3.4) nm; IR (neat) *v*_max_ 3418, 2924, 2854, 2362, and 1693 cm^−1^; ^1^H (600 MHz, CDCl_3_) (see Table 1) and ^13^C NMR (150 MHz, CDCl_3_) data (see Table 2); HRESIMS *m/z* 300.1572 [M + Na]^+^ (calcd for C_16_H_23_NO_3_Na, 300.1570).

*7β-Hydroperoxygorgosterol* (**3**): White solid; [α]^25^_D_ +12 (*c* 0.11, CHCl_3_); UV (MeOH) *λ*_max_ (log *ε*) 213 (3.5) nm; IR (neat) *v*_max_ 3380, 2933, 2850, 2362, and 1459 cm^−1^; ^1^H (600 MHz, CDCl_3_) (see Table 1) and ^13^C NMR (150 MHz, CDCl_3_) data (see Table 2); HRESIMS *m/z* 481.3651 [M + Na]^+^ (calculated for C_30_H_50_O_3_Na, 481.3652).

*7α-Hydroperoxygorgosterol* (**4**): White solid; [α]^25^_D_ −93 (*c* 0.07, CHCl_3_); UV (MeOH) *λ*_max_ (log *ε*) 213 (3.5) nm; IR (neat) *v*_max_ 3384, 2933, 2871, 2360, and 1457 cm^−1^; ^1^H (600 MHz, CDCl_3_) (see Table 1) and ^13^C NMR (150 MHz, CDCl_3_) data (see Table 2); HRESIMS *m/z* 481.3652 [M + Na]^+^ (calculated for C_30_H_50_O_3_Na, 481.3652).

*7β-Hydroperoxycampesterol* (**5**): White solid; [α]^25^_D_ +17 (*c* 0.12, CHCl_3_); UV (MeOH) *λ*_max_ (log *ε*) 239 (3.5) and 215 (3.5) nm; IR (neat) *v*_max_ 3383, 2933, 2868, 2360, and 1457 cm^−1^; ^1^H (600 MHz, CDCl_3_) (see Table 1) and ^13^C NMR (150 MHz, CDCl_3_) data (see Table 2); HRESIMS *m/z* 455.3495 [M + Na]^+^ (calculated for C_28_H_48_O_3_Na, 455.3496).

*7α-Hydroperoxycampesterol* (**6**): White solid; [α]^25^_D_ −134 (*c* 0.05, CHCl_3_); ^1^H (400 MHz, CDCl_3_) and ^13^C NMR (100 MHz, CDCl_3_) data (supplementary materials, Figures S45 and S46); ESIMS *m/z* 455 [M + Na]^+^, molecular formula C_28_H_48_O_3._

*7β-Hydroperoxycholesterol* (**7**): White solid; [α]^25^_D_ +43 (*c* 0.05, CHCl_3_); ^1^H (400 MHz, CDCl_3_) and ^13^C NMR (100 MHz, CDCl_3_) data (supplementary materials, Figures S47 and S48); ESIMS *m/z* 441 [M + Na]^+^, molecular formula C_27_H_46_O_3_.

*7α-Hydroperoxycholesterol* (**8**): White solid; [α]^25^_D_ −97 (*c* 0.03, CHCl_3_); ^1^H (400 MHz, CDCl_3_) and ^13^C NMR (100 MHz, CDCl_3_) data (supplementary materials, Figures S49 and S50); ESIMS *m/z* 441 [M + Na]^+^, molecular formula C_27_H_46_O_3_.

*Cespitulactam D* (**9**): White solid; [α]^25^_D_ −86 (*c* 0.05, CHCl_3_); ^1^H (400 MHz, CDCl_3_) and ^13^C NMR (100 MHz, CDCl_3_) data (supplementary materials, Figures S51 and S52); ESIMS *m/z* 338 [M + Na]^+^, molecular formula C_20_H_29_NO_2_.

*Cespitulactam F* (**10**): White solid; [α]^25^_D_ −168 (*c* 0.02, CHCl_3_); ^1^H (400 MHz, CDCl_3_) and ^13^C NMR (100 MHz, CDCl_3_) data (supplementary materials, Figures S53 and S54); ESIMS *m/z* 354 [M + Na]^+^, molecular formula C_20_H_29_NO_3_.

*Cespitulin S* (**11**): White solid; [α]^25^_D_ +13 (*c* 0.06, CHCl_3_); ^1^H (600 MHz, CDCl_3_) and ^13^C NMR (150 MHz, CDCl_3_) data (supplementary materials, Figures S55 and S56); ESIMS *m/z* 387 [M + Na]^+^, molecular formula C_21_H_32_O_5_.

*Cespitularin D* (**12**): White solid; [α]^25^_D_ −67 (*c* 0.03, CHCl_3_); ^1^H (400 MHz, CDCl_3_) and ^13^C NMR (100 MHz, CDCl_3_) data (supplementary materials, Figures S57 and S58); ESIMS *m/z* 355 [M + Na]^+^, molecular formula C_20_H_28_O_4_.

*Cespitularin O* (**13**): White solid; [α]^25^_D_ −25 (*c* 0.02, CH_2_Cl_2_); ^1^H (400 MHz, CDCl_3_) and ^13^C NMR (100 MHz, CDCl_3_) data (supplementary materials, Figures S59 and S60); ESIMS *m/z* 339 [M + Na]^+^, molecular formula C_20_H_28_O_3_.

*Cespitulactone B* (**14**): White solid; [α]^25^_D_ −148 (*c* 0.03, CHCl_3_); ^1^H (400 MHz, CDCl_3_) and ^13^C NMR (100 MHz, CDCl_3_) data (supplementary materials, Figures S61 and S62); ESIMS *m/z* 369 [M + Na]^+^, molecular formula C_21_H_30_O_4_.

*Cespihypotin Q* (**15**): White solid; [α]^25^_D_ −36 (*c* 0.06, CH_2_Cl_2_); ^1^H (400 MHz, CDCl_3_) and ^13^C NMR (100 MHz, CDCl_3_) data (supplementary materials, Figures S63 and S64); ESIMS *m/z* 385 [M + Na]^+^.

*Atractylenolide II* (**16**): White solid; [α]^25^_D_ +190 (*c* 0.08, CHCl_3_); ^1^H (400 MHz, CDCl_3_) and ^13^C NMR (100 MHz, CDCl_3_) data (supplementary materials, Figures S65 and S66); ESIMS *m/z* 255 [M + Na]^+^.

*Atractylenolide III* (**17**): White solid; [α]^25^_D_ +244 (*c* 0.41, CHCl_3_); ^1^H (400 MHz, CDCl_3_) and ^13^C NMR (100 MHz, CDCl_3_) data (supplementary materials, Figures S67 and S68); ESIMS *m/z* 271 [M + Na]^+^.

*Cespitulactam M-6*,*2′-diacetate* (**1a**): Cespitulactam M (**1**) (1.2 mg) in pyridine was mixed with Ac_2_O, and the mixture was stirred at room temperature for 24 h. After evaporation of excess reagent, the acetyl derivative of **1a** (1.0 mg) was yielded as a white solid. [α]^25^_D_ −100 (*c* 0.02, CHCl_3_); ^1^H (600 MHz, CDCl_3_) and ^13^C NMR (150 MHz, CDCl_3_) data (supplementary materials, Figures S9–S11); HRESIMS *m/z* 466.2565 [M + Na]^+^ (calcd for C_22_H_33_NO_3_Na, 466.2564).

### 3.4. Cytotoxicity Assay

Cell lines were purchased from the American Type Culture Collection (ATCC). Cytotoxicity of compounds **1**–**17** and **1a** were assayed using the Almar Blue assay [25,26]. Doxorubicin, employed as positive control, showed cytotoxic activity toward HepG2, MDA-MB231, and A549 cell lines with IC_50_ = 0.37, 0.30, and 0.15 μg/mL, respectively.

### 3.5. In Vitro Antibacterial Assay

The antibacterial assay of compounds **1**–**17** and **1a** was evaluated against *B. subtilis* (ATCC 6051), *E. aerogenes* (ATCC 13048), *E. coli* (ATCC 25922), *K. pneumoniae* (ATCC 10031), *S. enteritidis* (ATCC 13076), *S. typhimurium* (ATCC 14028), *S. marcescens* (ATCC 25419), *S. sonnei* (ATCC 11060), *S. aureus* (ATCC 9144), and *Y. enterocolitica* (ATCC 23715), by the procedures described previously [27].

### 3.6. In Vitro Anti-Inflammatory Assay

#### 3.6.1. Measurement of Cytokine Production by Dendritic Cells (DCs)

The experiment for measuring cytokine was tested by enzyme-link immunosorbent assay (ELISA) from the previously reported method [28,29]. The DCs were manipulated with lipopolysaccharide (LPS, 100 ng/mL) from *Escherichia coli* 055:B5 and the following treatment with the isolated compounds for 24 h. The optical density of the production of TNF-α was measured at 450 nm using the ELISA reader.

#### 3.6.2. Measurement of Nitric Oxide (NO) Production by DCs

DC cells were seeded in 24-well plates at a density of 1 × 10^6^ cells/mL. DCs were treated with each compound for 1 h and then stimulated with 100 ng/mL LPS for 24 h. The nitrite concentration in the medium was measured as an indicator of NO production through the Griess reaction. Briefly, 100 µL of cell culture supernatant was reacted with 100 µL of Griess reagent (1:1 mixture of 2% sulfanilamide and 0.2% *N*-(1-naphthyl)ethylenediamine dihydrochloride in water) in 96-well plate at room temperature for 10 min, and absorbance at 540 nm was recorded using sandwich ELISA assays [28,29].

#### 3.6.3. Statistical Analysis

The results are expressed as the mean ± SEM, and comparisons were made using one-way ANOVA by Tukey’s post hoc test (GraphPad Prism 5.0, GraphPad Software, San Diego, CA, USA). A probability value of 0.05 or less was considered significant. The software Sigma Plot was used for the statistical analysis.

## 4. Conclusions

In conclusion, a new nitrogen-containing verticillene diterpenoid, cespitulactam M (**1**); one new eudesmane sesquiterpenoid, cespilamide F (**2**); and three new hydroperoxysteroids (**3**–**5**) along with twelve known analogous metabolites (**6**–**17**) were isolated from a Formosan soft coral, *Cespitularia* sp. Subsequently, one new acetyl-derivative, cespitulactam M-6,2′-diacetate (**1a**), was prepared from compound **1**, and its bioactivities were evaluated. Furthermore, hydroperoxysteroids (**3**–**8**) were discovered in the genus of *Cespitularia* for the first time; in particular, 7β-hydroperoxygorgosterol (**3**) and 7α-hydroperoxygorgosterol (**4**) showed anti-inflammatory activities. Moreover, 7β-hydroperoxycampesterol (**5**) exhibited weak cytotoxicity and antibacterial activities. In this study, soft coral *Cespitularia* sp., with abundant natural product resources, resulted in a wide variety of chemical structures as well as diverse bioactivities for further research.

## Figures and Tables

**Figure 1:**
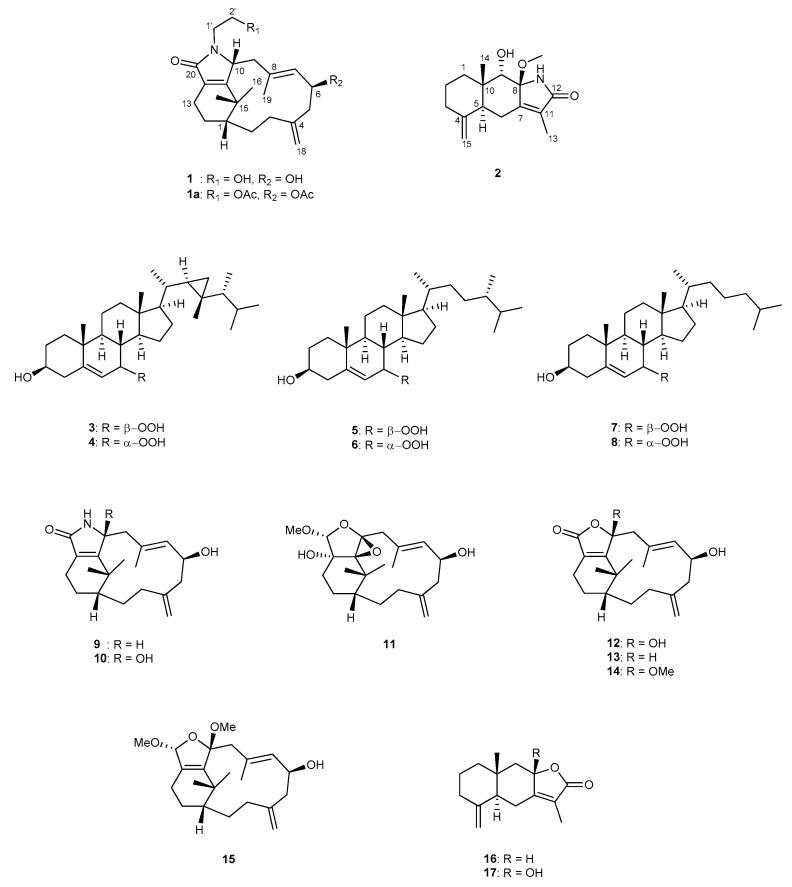
Structures of compounds **1**–**17**.

**Table 1:** ^1^H NMR spectroscopic data of compounds **1**–**5**.

Position	1	2	3	4	5
	*δ*_H_^a^	*δ*_H_^a^	*δ*_H_^a^	*δ*_H_^a^	*δ*_H_^a^
1	1.68 m	2.00 m 1.31 m	1.10 td (12.0, 4.2)	1.84 m 1.14 m	1.85 m 1.06 m
2	2.35 m 2.20 m	1.64 m	1.55 m	1.86 m	1.87 m 1.55 m
3	2.34 m	2.33 m 1.94 m	3.56 quint (4.8)	3.63 quint (5.4)	3.57 m
4			2.40 ddd (13.2, 4.8, 2.4) 2.29 tt (11.4, 2.4)	2.41 ddd (15.0, 5.4, 1.8) 2.33 tt (11.4, 2.4)	2.40 ddd (13.2, 4.8, 2.4) 2.29 tt (9.0, 2.4)
5	2.36 m	2.28 d (12.0)			
6	4.35 m	2.54 dd (13.2, 3.0) 2.16 m	5.58 t (1.8)	5.73 dd (5.4, 1.8)	5.58 t (2.4)
7	5,41 d (8.4) ^b^		4.15 dt (8.4, 1.8)	4.17 td (4.8, 1.8)	4.15 dt (9.0, 2.4)
8			1.59 m	1.61 m	1.65 m
9	2.74 br d (14.4) 2.68 dd (14.4, 4.2)	3.51 s	1.09 m	1.41 m	1.09 m
10	4.32 br s				
11			1.55 m 1.46 m	1.48 m	1.56 m
12			2.03 m 1.18 m	1.98 m 1.18 m	2.02 dt (12.6, 3.6) 1.14 m
13	1.57 m	1.85 s			
14	1.69 m	1.00 s	1.10 m	1.47 m	1.10 m
15		4.86 s 4.59 s	1.37 m	1.89 m 1.12 m	1.77 m 1.35 m
16	1.17 s		1.36 m	2.10 m 1.33 m	1.87 m 1.29 m
17	1.40 s		1.23 m	1.31 m	1.17 m
18	4.82 d (6.0)		0.67 s	0.65 s	0.69 s
19	1.43 s		1.04 s	1.00 s	1.05 s
20			1.60 m	1.02 m	1.37 m
21			1.01 d (4.8)	1.04 br s	0.92 d (6.6)
22			0.18 m	0.18 m	1.40 m 0.95 m
23					1.37 m 0.93 m
24			0.24 m	0.24 m	1.18 m
25			1.56 m	1.54 m	1.57 m
26			0.95 d (7.8)	0.96 d (6.6)	0.86 d (7.2)
27			0.85 d (5.4)	0.85 d (7.2)	0.79 d (6.6)
28			0.94 d (7.8)	0.94 d (7.2)	0.78 d (6.6)
29			0.46 dd (9.0, 4.2) −0.12 dd (6.0, 4.2)	0.46 dd (9.6, 4.2) −0.13 dd (6.0, 4.2)	
30			0.90 s	0.91 s	
1′	3.92 ddd (15.0, 7.8, 3.0) 3.34 ddd (15.0, 7.8, 3.0)				
2′	3.85 m				
7−OOH			7.45 s	7.59 s	7.48 s
8−OMe		3.09 s			

^a^ Spectrum recorded at 600 MHz in CDCl_3_. ^b^ *J* values (in Hz) in parentheses.

**Table 2:** ^13^C NMR spectroscopic data of compounds **1**–**5**.

Position	1	2	3	4	5
	*δ*_C_^a^	*δ*_C_^a^	*δ*_C_^a^	*δ*_C_^a^	*δ*_C_^a^
1	43.1 (CH) ^b^	34.8 (CH_2_)	36.8 (CH_2_)	36.7 (CH_2_)	36.8 (CH_2_)
2	18.2 (CH_2_)	22.2 (CH_2_)	31.6 (CH_2_)	31.3 (CH_2_)	31.6 (CH_2_)
3	32.5 (CH_2_)	36.1 (CH_2_)	71.3 (CH)	71.4 (CH)	71.3 (CH)
4	146.5 (C)	No detected (C)	41.9 (CH_2_)	42.2 (CH_2_)	41.8 (CH_2_)
5	43.8 (CH_2_)	43.9 (CH)	146.0 (C)	148.9 (C)	146.1 (C)
6	68.3 (CH)	24.3 (CH_2_)	121.5 (CH)	119.9 (CH)	121.5 (CH)
7	134.2 (CH)	151.8 (C)	86.6 (CH)	78.5 (CH)	86.6 (CH)
8	133.4 (C)	93.1 (C)	34.6 (CH)	37.1 (CH)	34.5 (CH)
9	38.5 (CH_2_)	78.7 (CH)	48.7 (CH)	43.5 (CH)	48.7 (CH)
10	62.4 (CH)	40.5 (C)	36.4 (C)	37.4 (C)	36.4 (C)
11	161.3 (C)	130.0 (C)	21.3 (CH_2_)	20.9 (CH_2_)	21.3 (CH_2_)
12	131.6 (C)	174.3 (C)	39.6 (CH_2_)	39.1 (CH_2_)	39.5 (CH_2_)
13	32.0 (CH_2_)	8.1 (CH_3_)	43.3 (C)	42.8 (C)	42.8 (C)
14	24.3 (CH_2_)	16.2 (CH_3_)	55.8 (CH)	48.9 (CH)	55.4 (CH)
15	37.1 (C)	106.6 (CH_2_)	26.2 (CH_2_)	24.7 (CH_2_)	26.0 (CH_2_)
16	35.1 (CH_3_)		28.3 (CH_2_)	28.2 (CH_2_)	28.3 (CH_2_)
17	25.3 (CH_3_)		57.4 (CH)	57.5 (CH)	55.9 (CH)
18	113.8 (CH_2_)		11.9 (CH_3_)	11.3 (CH_3_)	11.8 (CH_3_)
19	17.2 (CH_3_)		18.8 (CH_3_)	18.2 (CH_3_)	18.8 (CH_3_)
20	172.5 (C)		35.2 (CH)	35.4 (CH)	36.1 (CH)
21			21.2 (CH_3_)	21.2 (CH_3_)	18.9 (CH_3_)
22			32.0 (CH)	32.0 (CH)	33.7 (CH_2_)
23			25.8 (C)	25.8 (C)	30.6 (CH_2_)
24			50.8 (CH)	50.8 (CH)	39.1 (CH)
25			32.1 (CH)	32.2 (CH)	31.4 (CH)
26			22.2 (CH_3_)	22.2 (CH_3_)	20.5 (CH_3_)
27			21.5 (CH_3_)	21.5 (CH_3_)	17.6 (CH_3_)
28			15.4 (CH_3_)	15.5 (CH_3_)	15.4 (CH_3_)
29			21.3 (CH_2_)	21.3 (CH_2_)	
30			14.3 (CH_3_)	14.3 (CH_3_)	
1′	44.5 (CH_2_)				
2′	62.2 (CH_2_)				
8−OMe		49.6 (CH_3_)			

^a^ Spectrum recorded at 150 MHz in CDCl_3_. ^b^ Attached protons were deduced by the DEPT experiment.

## Data Availability

Data from the present study are available in the article and Supplementary Materials.

## Supplementary Materials

The following supporting information can be downloaded at: https://www.mdpi.com/article/10.3390/molecules28041521/s1, Figures S1–S68: ESIMS and NMR spectra of compounds **1**–**17**, Tables S1–S3: the results of cytotoxic, antibacterial, and anti-inflammatory activities.

## Sample Availability

Samples of the compounds are not available from the authors.
