# Supplementary material for: New Verticillene Diterpenoids, Eudesmane Sesquiterpenoids, and Hydroperoxysteroids from the Further Chemical Investigation of a Taiwanese Soft Coral Cespitularia sp
Source: Molecules. 2023 Feb 4;28(4):1521. doi: 10.3390/molecules28041521 (PMC9965867; doi:10.3390/molecules28041521)
Supplement: Supplementary file 1 [file molecules-28-01521-s001.zip › molecules-2196217-supplementary.pdf]

Supporting Information

Further Verticillene Diterpenoids, Eudesmane Sesquiterpe-noids, and Hydroperoxysteroids from a Taiwanese Soft Coral *Cespitularia* sp.

Chung-Wei Fu[1], You-Cheng Lin[2], Shu-Fen Chiou[1], Shu-Li Chen[3], Chi-Chien Lin[4], Hui-Chun Wang[3], Chang-Feng Dai[5], and Jyh-Horng Sheu[1,2,3,6*]

[1]Department of Marine Biotechnology and Resources, National Sun Yat-sen University, Kaohsiung 804, Taiwan

[2]Doctoral Degree Program in Marine Biotechnology, National Sun Yat-sen University, Kaohsiung 804, Taiwan

[3]Graduate Institute of Natural Products, Kaohsiung Medical University, Kaohsiung 807, Taiwan

[4]Institute of Biomedical Sciences, National Chung-Hsing University, Taichung 402, Taiwan

[5]Institute of Oceanography, National Taiwan University, Taipei 106, Taiwan

[6]Department of Medical Research, China Medical University Hospital, China Medical University, Taichung 404, Taiwan

*** Correspondence:**

Prof. Jyh-Horng Sheu

sheu@mail.nsysu.edu.tw

No.	Content	page

List of Figures

Figure S1. HRESIMS of compound **1** 5
Figure S2. ^{1}H NMR spectrum (600MHz) of compound **1** in $CDCl_3$ 5
Figure S3. ^{13}C NMR spectrum (150 MHz) of compound **1** in $CDCl_3$ 6
Figure S4. DEPT spectrum (150 MHz) of compound **1** in $CDCl_3$ 6
Figure S5. HSQC spectrum of compound **1** in $CDCl_3$ 7
Figure S6. ^{1}H-^{1}H COSY spectrum of compound **1** in $CDCl_3$ 7
Figure S7. HMBC spectrum of compound **1** in $CDCl_3$ 8
Figure S8. NOESY spectrum of compound **1** in $CDCl_3$ 8
Figure S9. HRESIMS of compound **1a** 9
Figure S10. ^{1}H NMR spectrum (600MHz) of compound **1a** in $CDCl_3$ 9
Figure S11. ^{13}C NMR spectrum (150 MHz) of compound **1a** in $CDCl_3$. 10
Figure S12. HRESIMS of compound **2** 10
Figure S13. ^{1}H NMR spectrum (600MHz) of compound **2** in $CDCl_3$ 11
Figure S14. ^{13}C NMR spectrum (150 MHz) of compound **2** in $CDCl_3$ 11
Figure S15. DEPT spectrum (150 MHz) of compound **2** in $CDCl_3$ 12
Figure S16. HSQC spectrum of compound **2** in $CDCl_3$ 12
Figure S17. ^{1}H-^{1}H COSY spectrum of compound **2** in $CDCl_3$ 13
Figure S18. HMBC spectrum of compound **2** in $CDCl_3$ 13
Figure S19. The amplificatory HMBC spectrum of compound **2** in $CDCl_3$ 14
Figure S20. NOESY spectrum of compound **2** in $CDCl_3$ 14
Figure S21. HRESIMS of compound **3** 15
Figure S22. ^{1}H NMR spectrum (600MHz) of compound **3** in $CDCl_3$ 15
Figure S23. ^{13}C NMR spectrum (150 MHz) of compound **3** in $CDCl_3$ 16
Figure S24. DEPT spectrum (150 MHz) of compound **3** in $CDCl_3$ 16
Figure S25. HSQC spectrum of compound **3** in $CDCl_3$ 17
Figure S26. ^{1}H-^{1}H COSY spectrum of compound **3** in $CDCl_3$ 17
Figure S27. HMBC spectrum of compound **3** in $CDCl_3$ 18
Figure S28. NOESY spectrum of compound **3** in $CDCl_3$ 18
Figure S29. HRESIMS of compound **4** 19
Figure S30. ^{1}H NMR spectrum (600MHz) of compound **4** in $CDCl_3$ 19
Figure S31. ^{13}C NMR spectrum (150 MHz) of compound **4** in $CDCl_3$ 20
Figure S32. DEPT spectrum (150 MHz) of compound **4** in $CDCl_3$ 20

Figure S33. HSQC spectrum of compound **4** in $CDCl_3$........................... 21
Figure S34. ^1H-^1H COSY spectrum of compound **4** in $CDCl_3$................ 21
Figure S35. HMBC spectrum of compound **4** in $CDCl_3$.......................... 22
Figure S36. NOESY spectrum of compound **4** in $CDCl_3$ 22
Figure S37. HRESIMS of compound **5** 23
Figure S38. ^1H NMR spectrum (600MHz) of compound **5** in $CDCl_3$..... 23
Figure S39. ^{13}C NMR spectrum (150 MHz) of compound **5** in $CDCl_3$ ... 24
Figure S40. DEPT spectrum (150 MHz) of compound **5** in $CDCl_3$......... 24
Figure S41. HSQC spectrum of compound **5** in $CDCl_3$........................... 25
Figure S42. ^1H-^1H COSY spectrum of compound **5** in $CDCl_3$................ 25
Figure S43. HMBC spectrum of compound **5** in $CDCl_3$.......................... 26
Figure S44. NOESY spectrum of compound **5** in $CDCl_3$ 26
Figure S45. ^1H NMR spectrum (400MHz) of compound **6** in $CDCl_3$..... 27
Figure S46. ^{13}C NMR spectrum (100 MHz) of compound **6** in $CDCl_3$ ... 27
Figure S47. ^1H NMR spectrum (400MHz) of compound **7** in $CDCl_3$..... 28
Figure S48. ^{13}C NMR spectrum (100 MHz) of compound **7** in $CDCl_3$ ... 28
Figure S49. ^1H NMR spectrum (400MHz) of compound **8** in $CDCl_3$..... 29
Figure S50. ^{13}C NMR spectrum (100 MHz) of compound **8** in $CDCl_3$ ... 29
Figure S51. ^1H NMR spectrum (400MHz) of compound **9** in $CDCl_3$..... 30
Figure S52. ^{13}C NMR spectrum (100 MHz) of compound **9** in $CDCl_3$ ... 30
Figure S53. ^1H NMR spectrum (400MHz) of compound **10** in $CDCl_3$... 31
Figure S54. ^{13}C NMR spectrum (100 MHz) of compound **10** in $CDCl_3$. 31
Figure S55. ^1H NMR spectrum (600MHz) of compound **11** in $CDCl_3$... 32
Figure S56. ^{13}C NMR spectrum (150 MHz) of compound **11** in $CDCl_3$. 32
Figure S57. ^1H NMR spectrum (400MHz) of compound **12** in $CDCl_3$... 33
Figure S58. ^{13}C NMR spectrum (100 MHz) of compound **12** in $CDCl_3$. 33
Figure S59. ^1H NMR spectrum (400MHz) of compound **13** in $CDCl_3$... 34
Figure S60. ^{13}C NMR spectrum (100 MHz) of compound **13** in $CDCl_3$. 34
Figure S61. ^1H NMR spectrum (400MHz) of compound **14** in $CDCl_3$... 35
Figure S62. ^{13}C NMR spectrum (100 MHz) of compound **14** in $CDCl_3$. 35
Figure S63. ^1H NMR spectrum (400MHz) of compound **15** in $CDCl_3$... 36
Figure S64. ^{13}C NMR spectrum (100 MHz) of compound **15** in $CDCl_3$. 36
Figure S65. ^1H NMR spectrum (400MHz) of compound **16** in $CDCl_3$... 37
Figure S66. ^{13}C NMR spectrum (100 MHz) of compound **16** in $CDCl_3$. 37
Figure S67. ^1H NMR spectrum (400MHz) of compound **17** in $CDCl_3$... 38
Figure S68. ^{13}C NMR spectrum (100 MHz) of compound **17** in $CDCl_3$. 38

List of Tables

Table S1. The cytotoxicity data of compounds **1–16** and **1a**. 39
Table S2. Antibacterial activity (zone of inhibition in mm). 40
Table S3. Inhibitory effects of compounds **1–10**, **1a**, **14**, and **15** on TNF-α expression, PGE_2, and NO production in LPS induced dendritic cells. 41

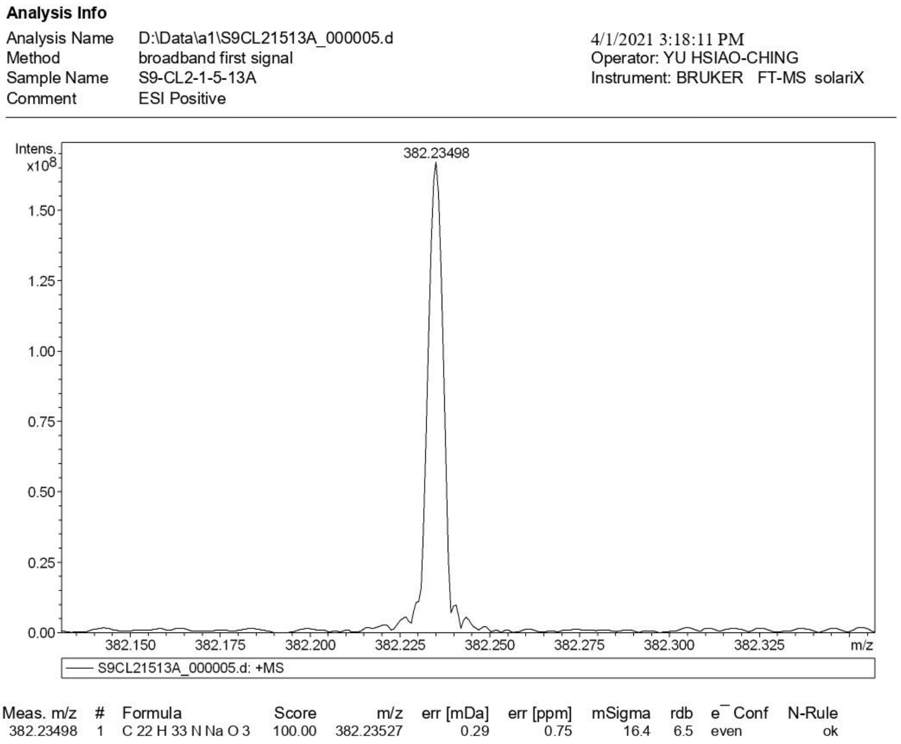

Figure S1. HRESIMS of compound **1**

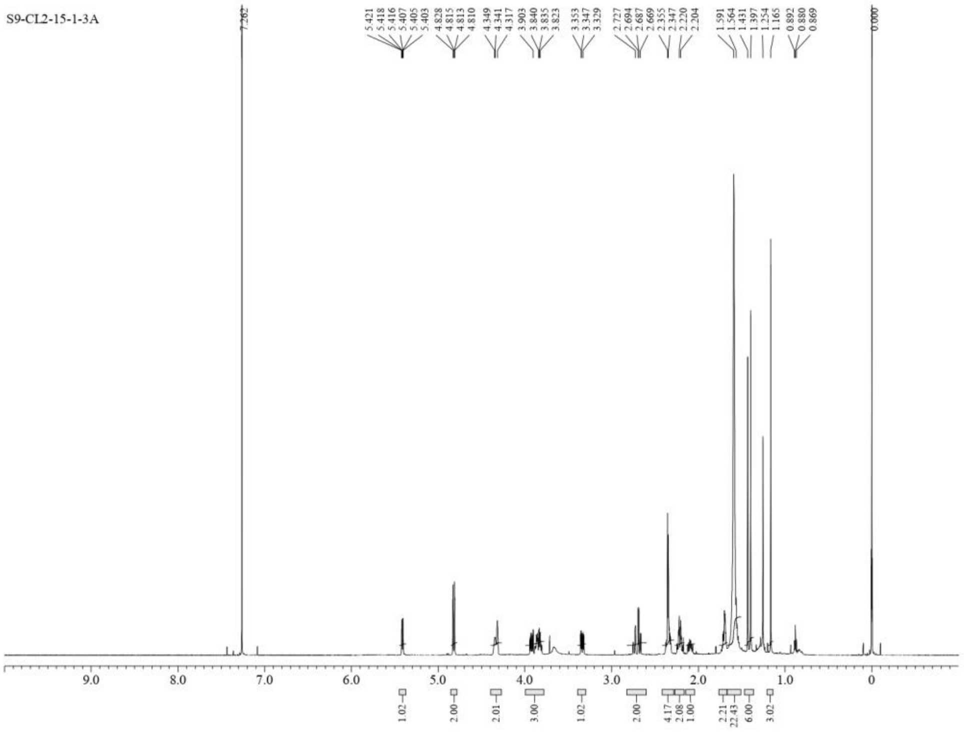

Figure S2. ^{1}H NMR spectrum (600MHz) of compound **1** in $CDCl_3$

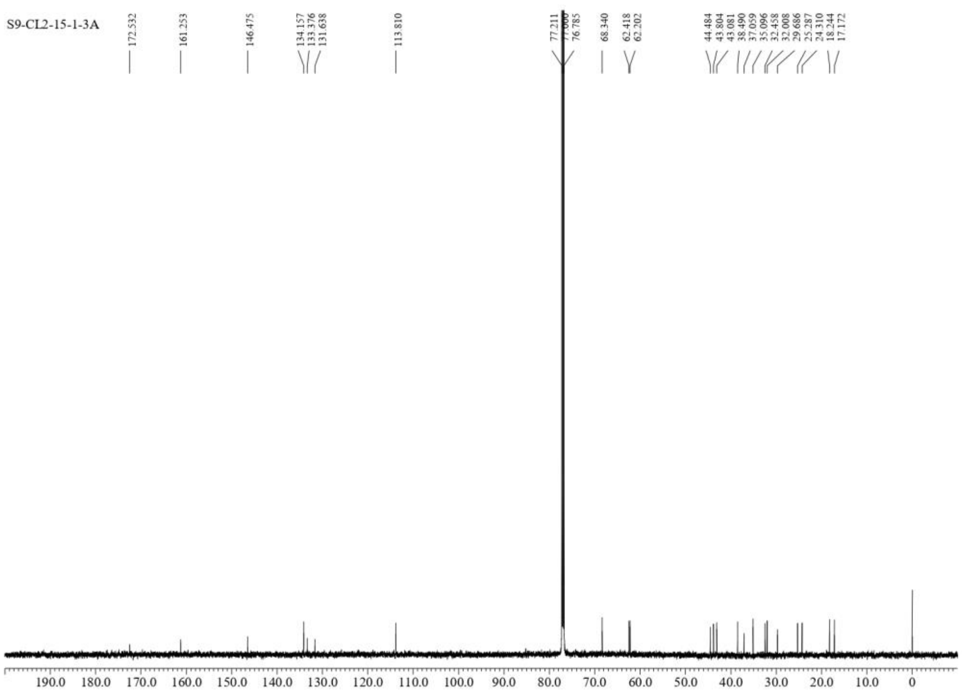

Figure S3. ^{13}C NMR spectrum (150 MHz) of compound **1** in $CDCl_3$

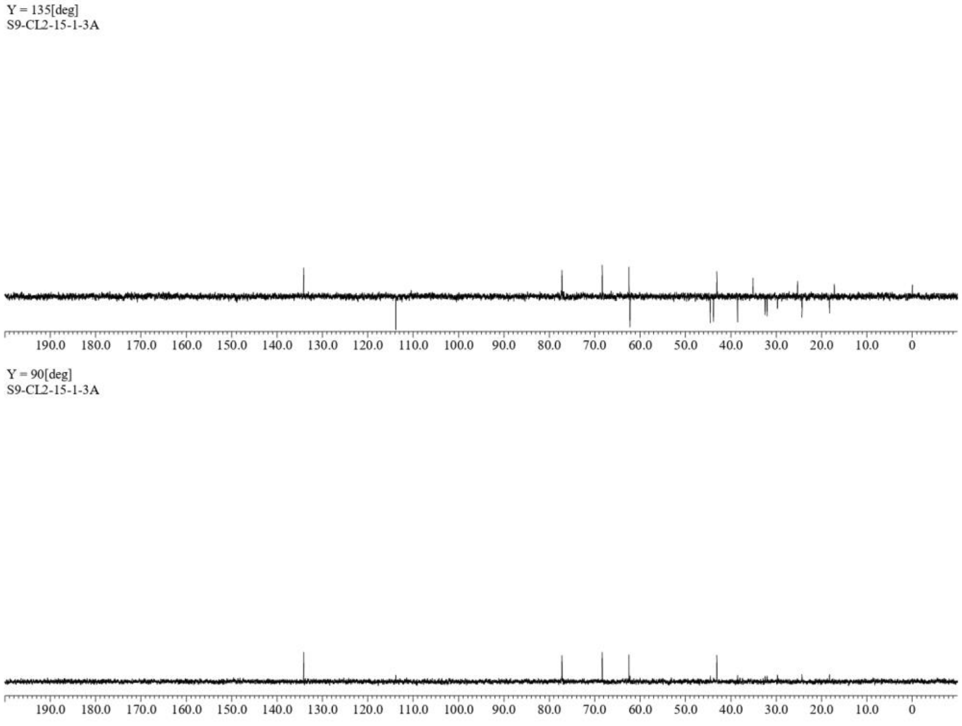

Figure S4. DEPT spectrum (150 MHz) of compound **1** in $CDCl_3$

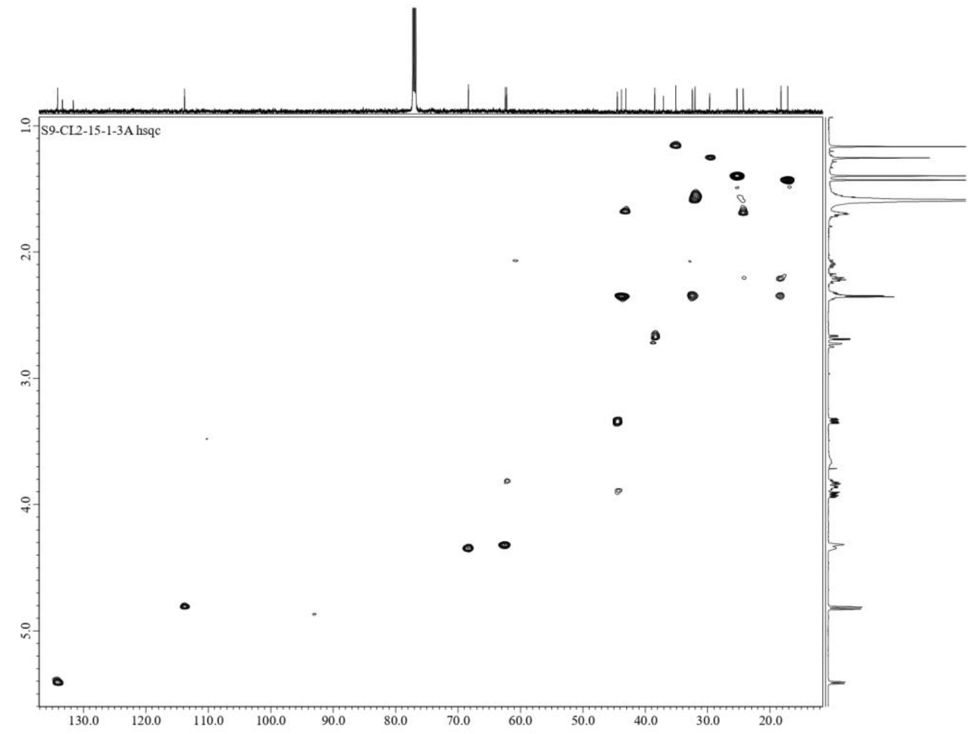

Figure S5. HSQC spectrum of compound **1** in $CDCl_3$

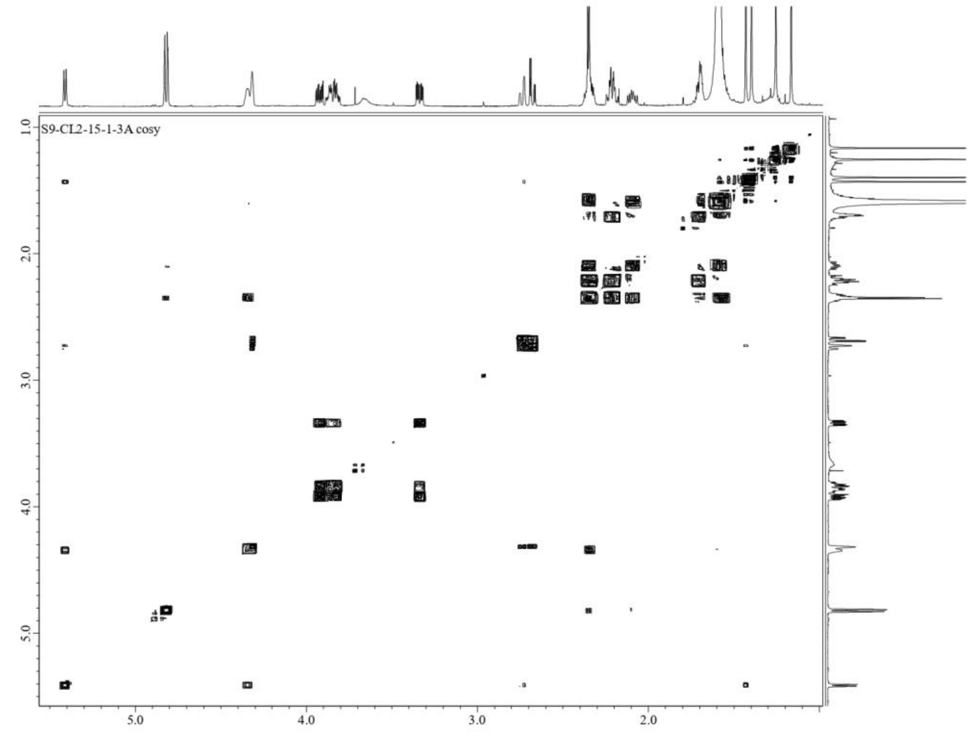

Figure S6. ^{1}H-^{1}H COSY spectrum of compound **1** in $CDCl_3$

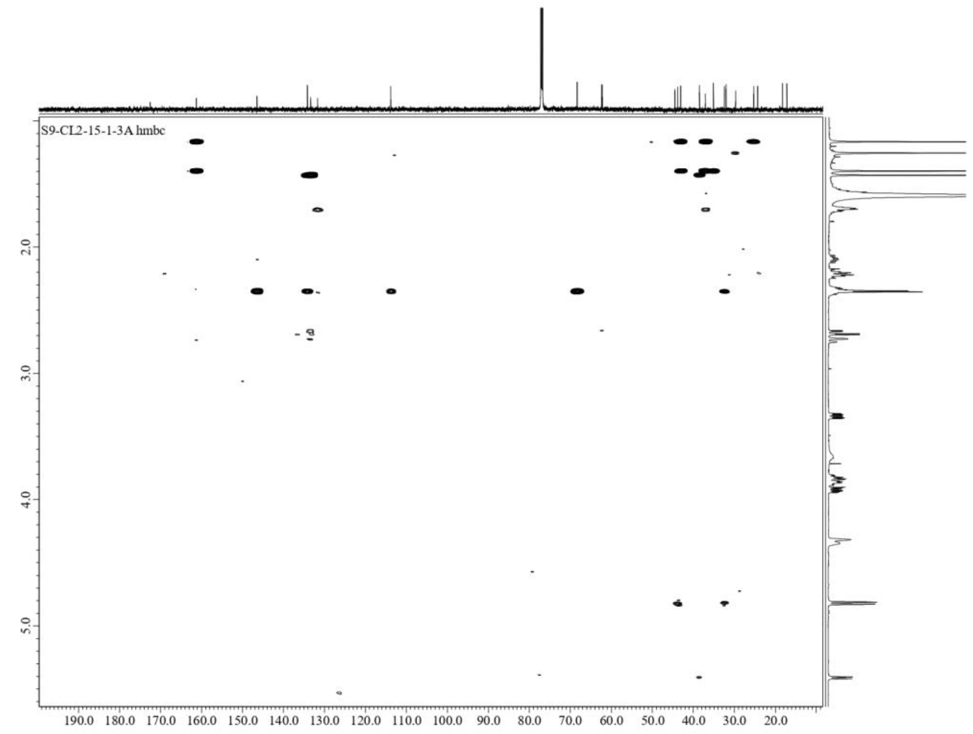

Figure S7. HMBC spectrum of compound **1** in $CDCl_3$

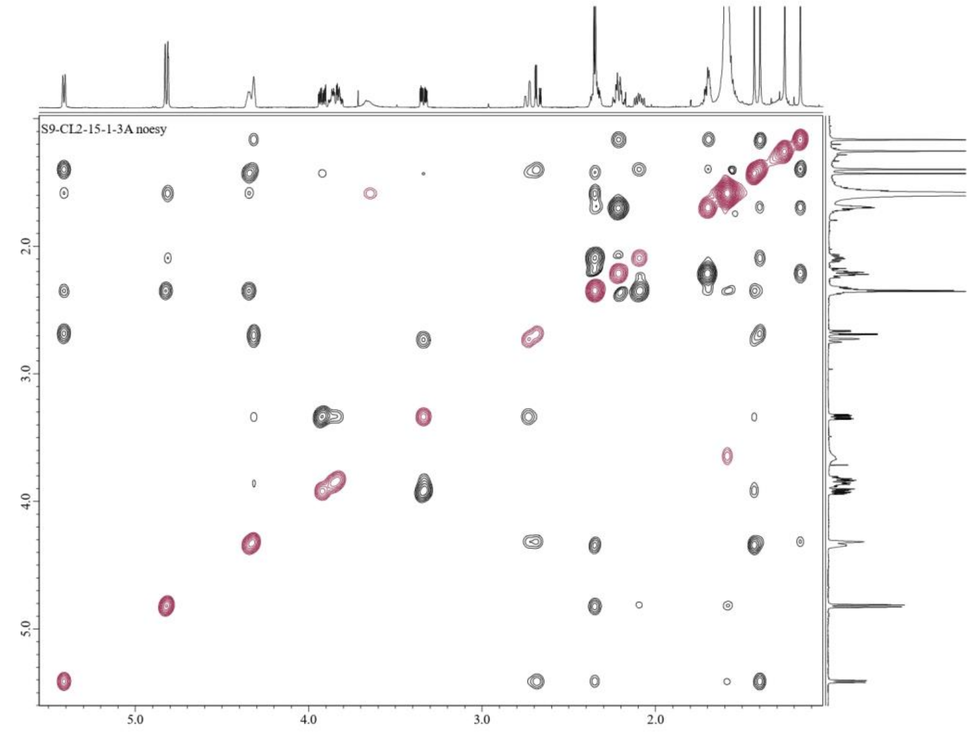

Figure S8. NOESY spectrum of compound **1** in $CDCl_3$

Mass Spectrum SmartFormula Report

Analysis Info

Analysis Name	D:\Data\a1\S9CL21513A2_000002.d	9/9/2021 12:39:49 PM
Method	broadband first signal	Operator: YU HSIAO-CHING
Sample Name	S9-CL2-15-1-3A-2	Instrument: BRUKER FT-MS solariX
Comment	ESI Positive	

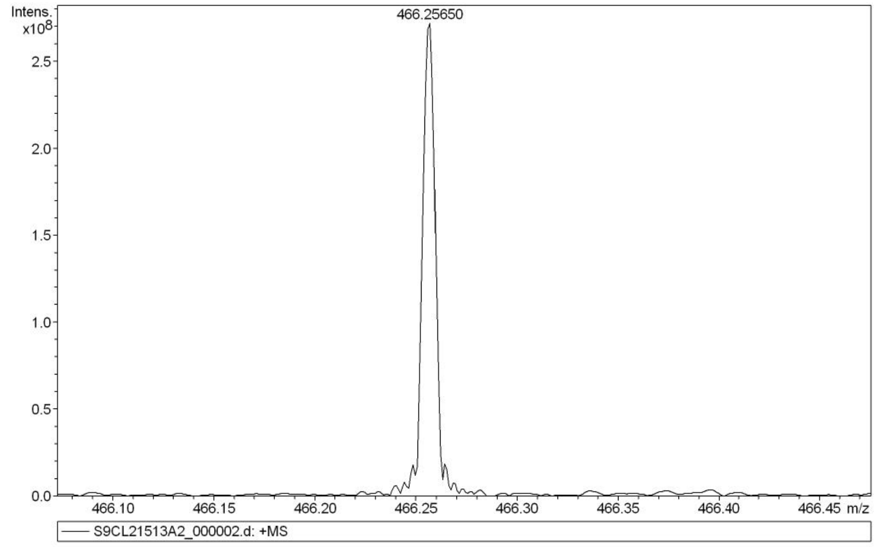

Meas. m/z	#	Formula	Score	m/z	err [mDa]	err [ppm]	mSigma	rdb	e⁻ Conf	N-Rule
466.25650	1	C 26 H 37 N Na O 5	100.00	466.25639	-0.11	-0.23	13.6	8.5	even	ok

Figure S9. HRESIMS of compound **1a**

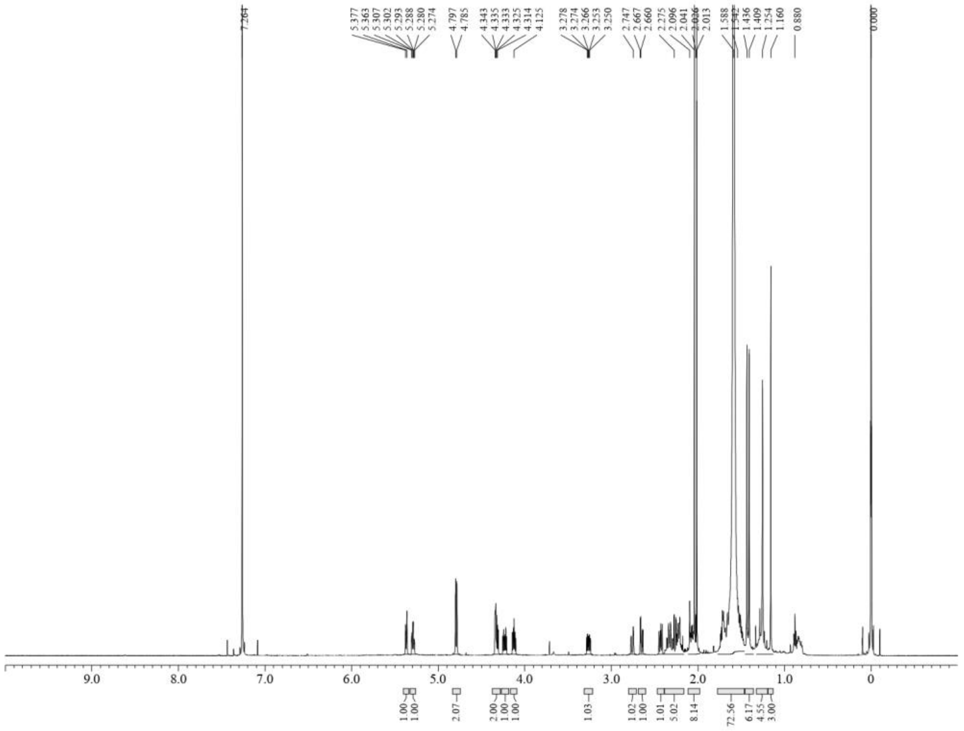

Figure S10. ^{1}H NMR spectrum (600MHz) of compound **1a** in $CDCl_3$

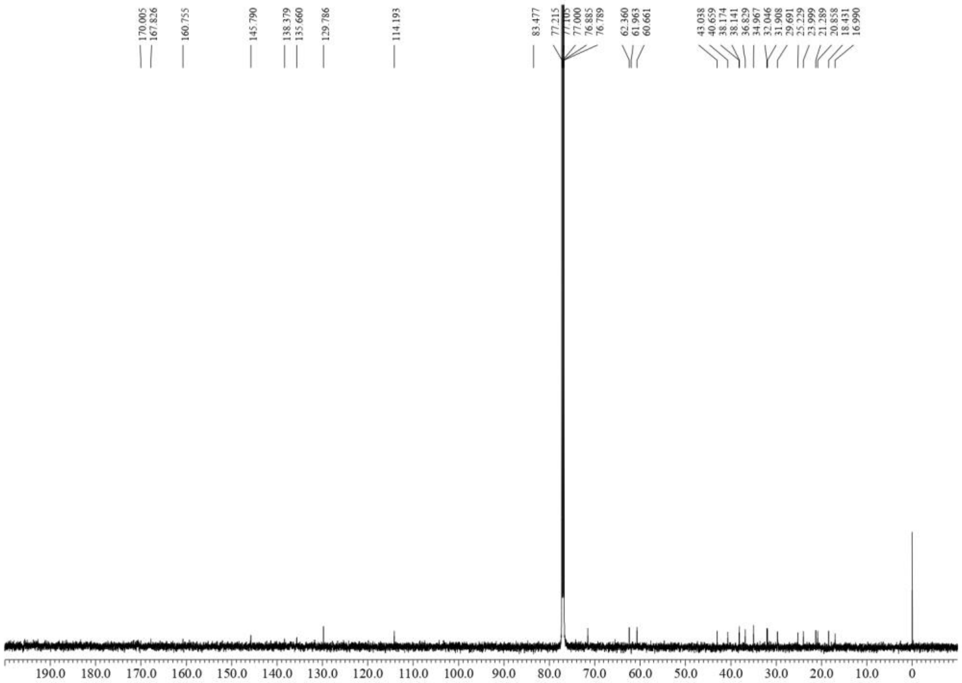

Figure S11. ^{13}C NMR spectrum (150 MHz) of compound **1a** in $CDCl_3$

Analysis Info

Analysis Name	D:\Data\a1\S9CL285D_000004.d	4/12/2021 5:11:11 PM
Method	broadband first signal	Operator: YU HSIAO-CHING
Sample Name	S9-CL2-8-5D	Instrument: BRUKER FT-MS solariX
Comment	ESI Positive	

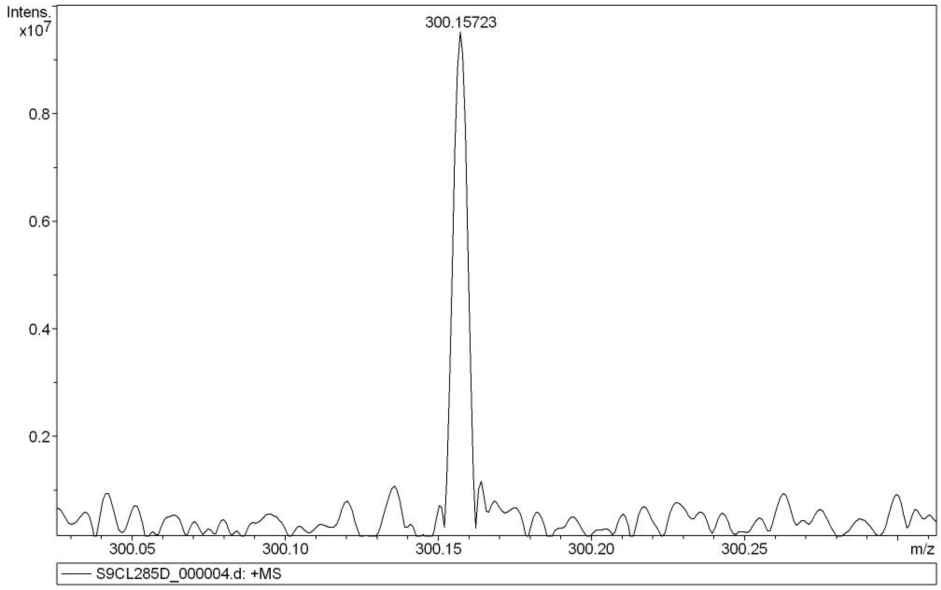

Meas. m/z	#	Formula	Score	m/z	err [mDa]	err [ppm]	mSigma	rdb	e⁻ Conf	N-Rule
300.15723	1	C 16 H 23 N Na O 3	100.00	300.15701	-0.22	-0.72	16.8	5.5	even	ok

Figure S12. HRESIMS of compound **2**

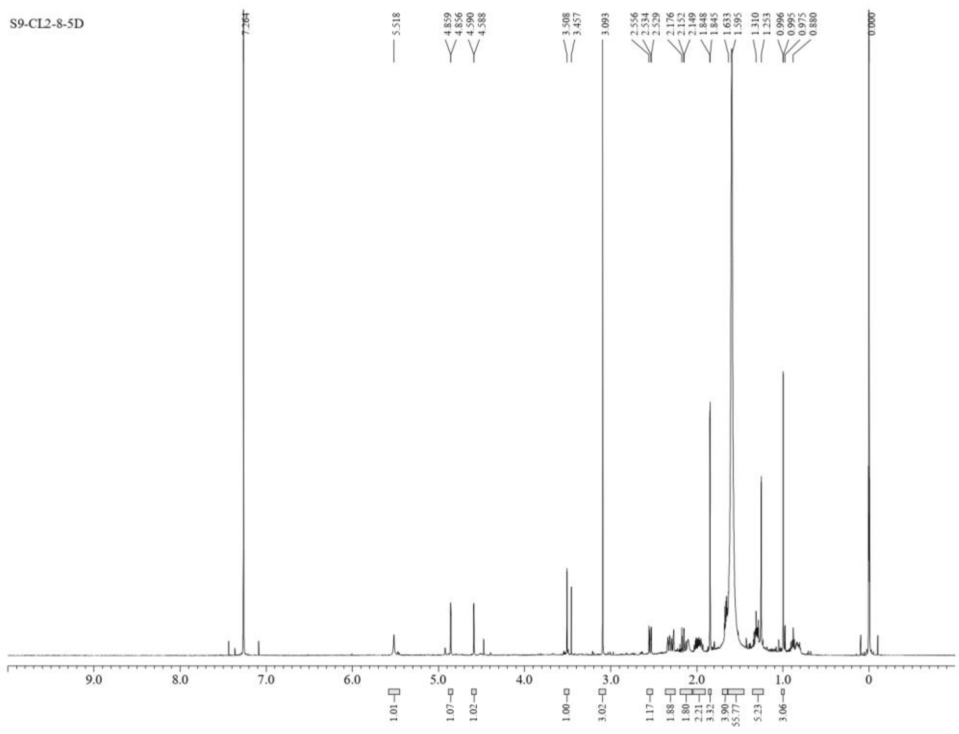

Figure S13. ^{1}H NMR spectrum (600MHz) of compound **2** in $CDCl_3$

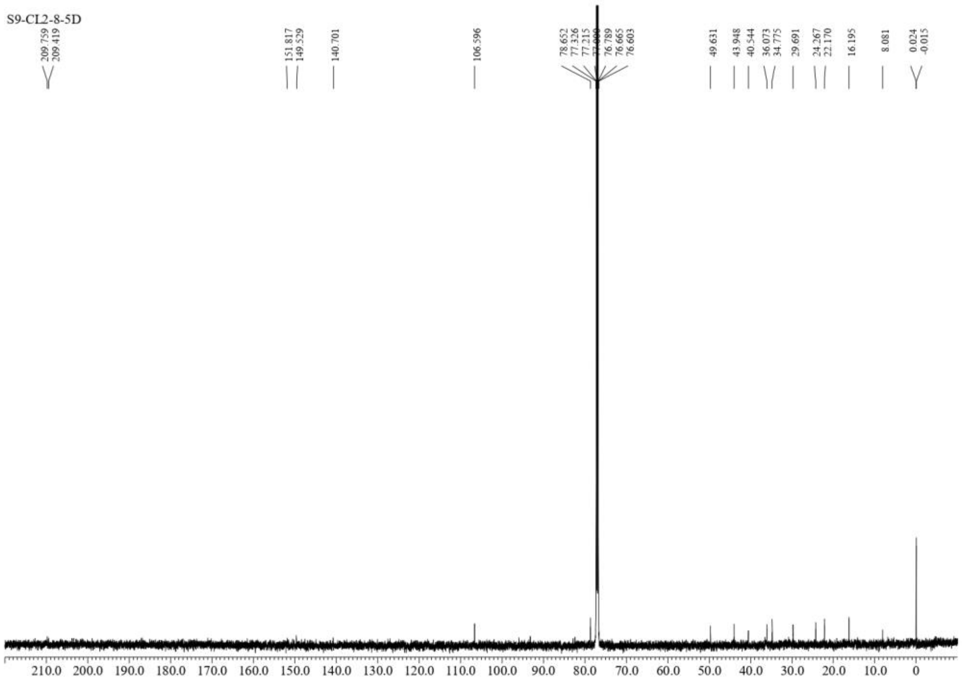

Figure S14. ^{13}C NMR spectrum (150 MHz) of compound **2** in $CDCl_3$

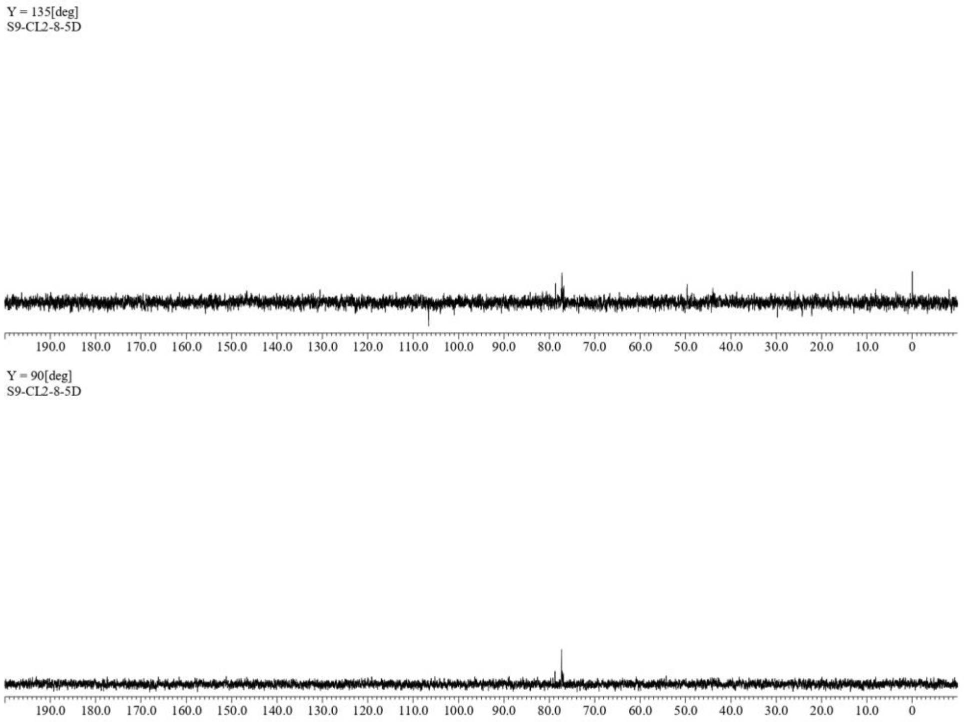

Figure S15. DEPT spectrum (150 MHz) of compound **2** in $CDCl_3$

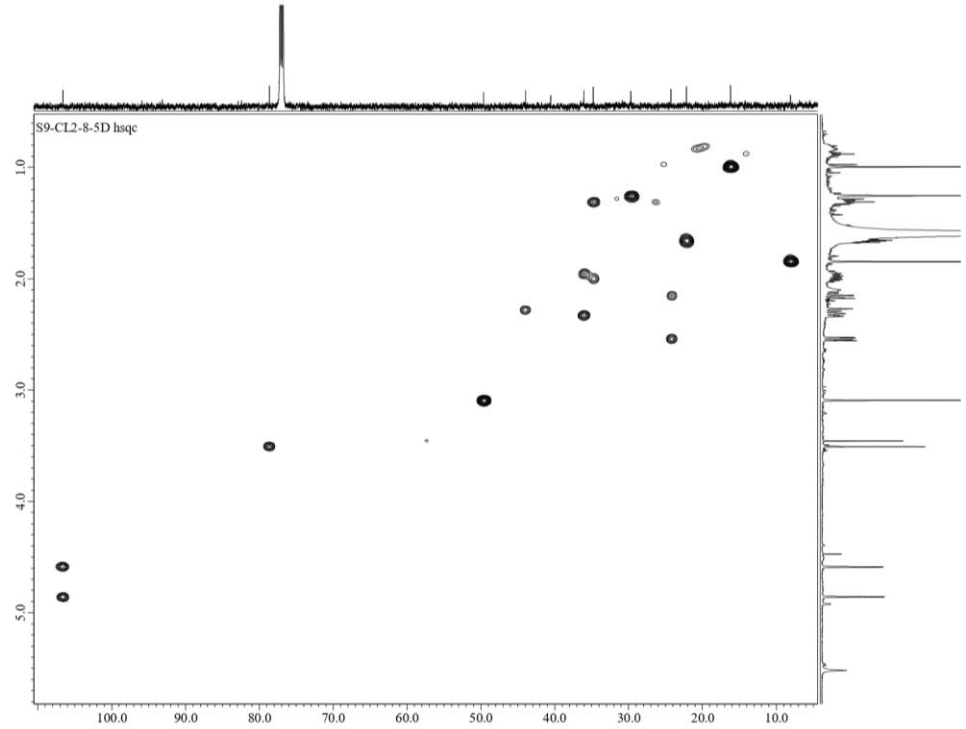

Figure S16. HSQC spectrum of compound **2** in $CDCl_3$

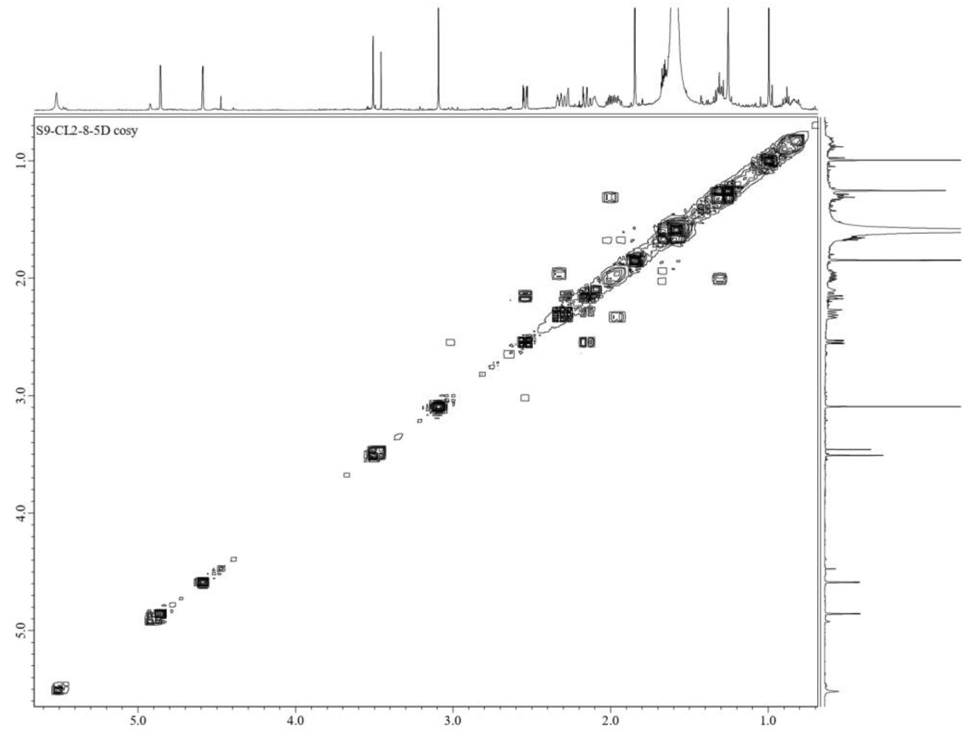

Figure S17. ^{1}H-^{1}H COSY spectrum of compound **2** in $CDCl_3$

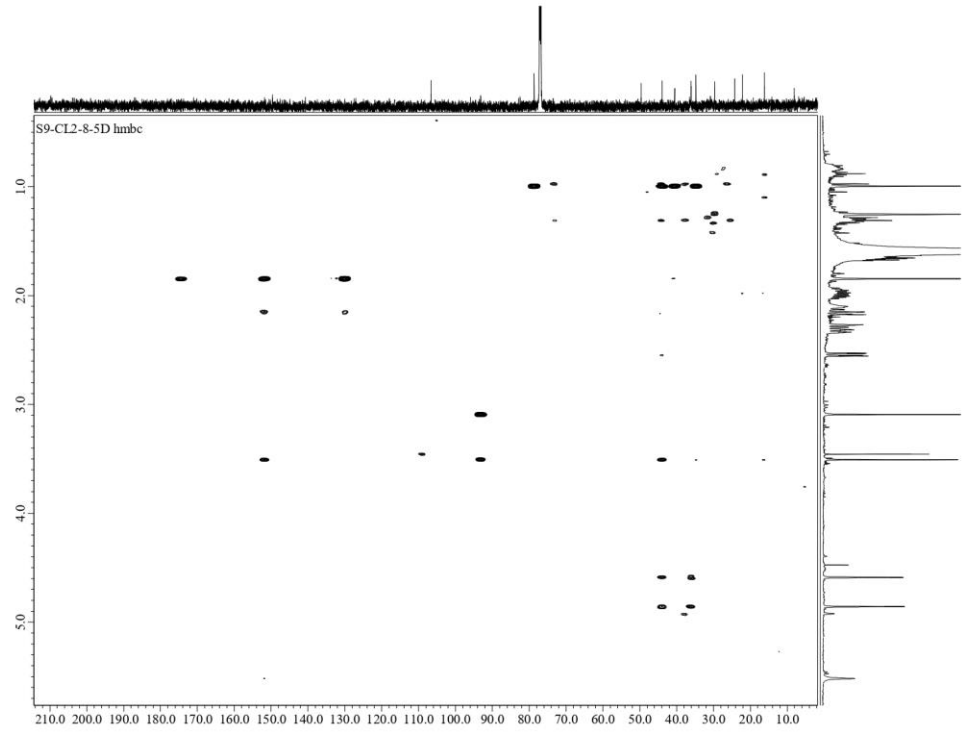

Figure S18. HMBC spectrum of compound **2** in $CDCl_3$

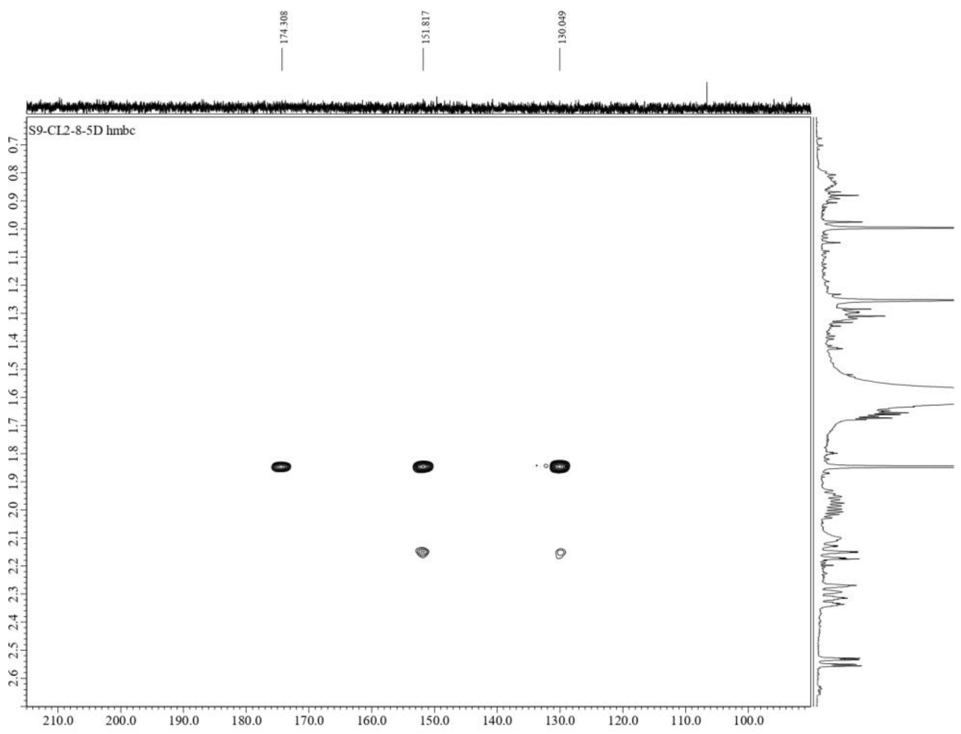

Figure S19. The amplificatory HMBC spectrum of compound **2** in $CDCl_3$

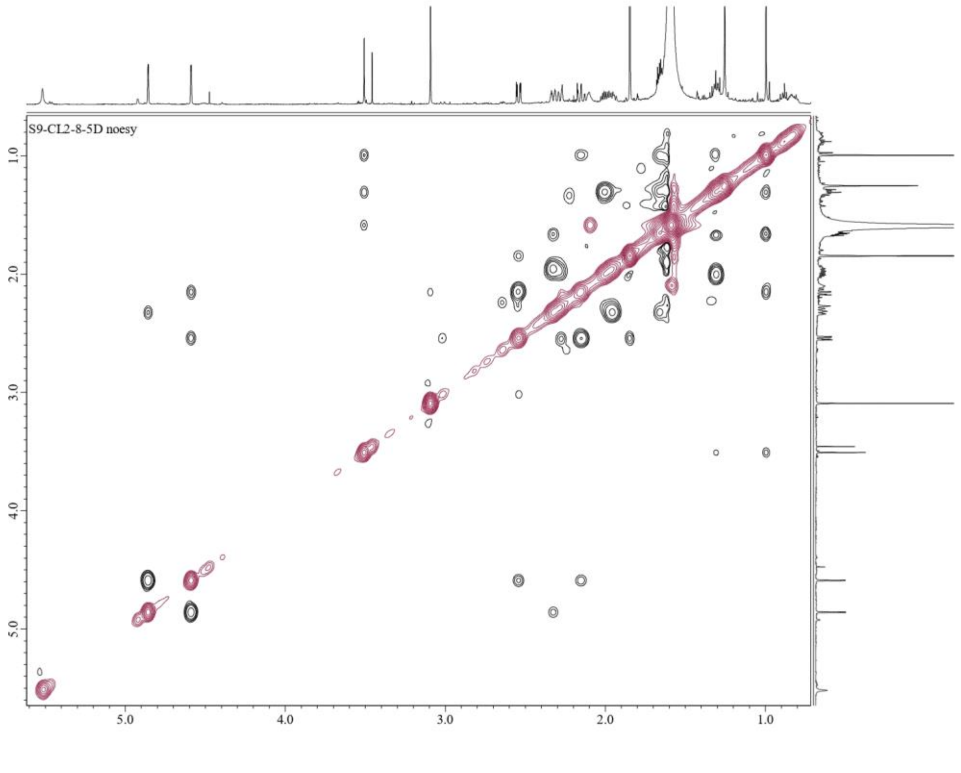

Figure S20. NOESY spectrum of compound **2** in $CDCl_3$

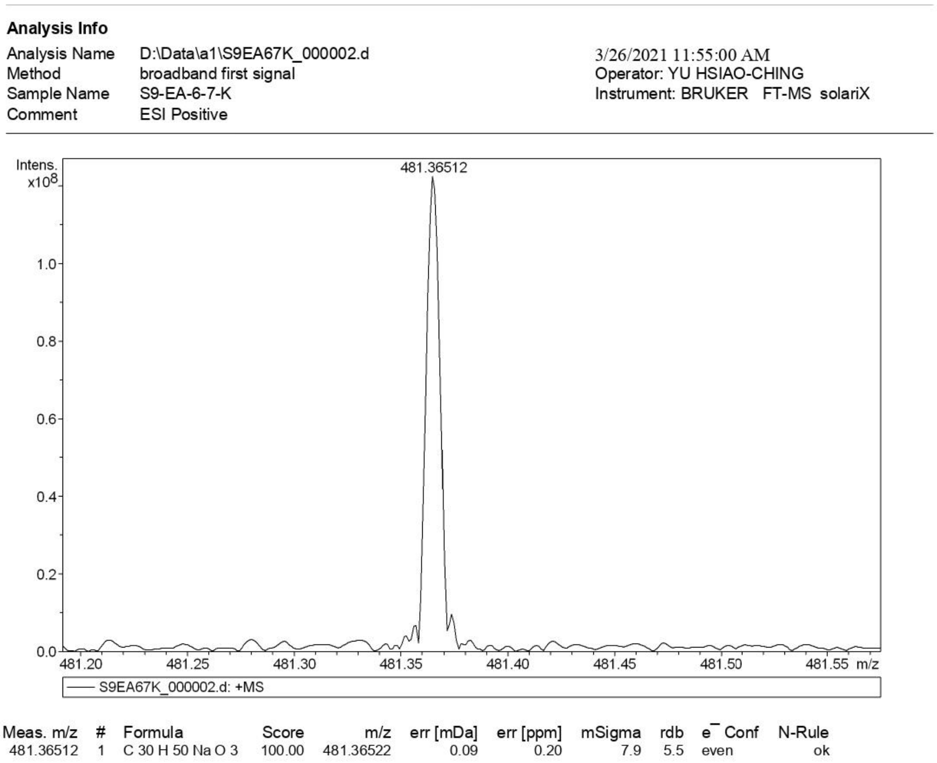

Figure S21. HRESIMS of compound **3**

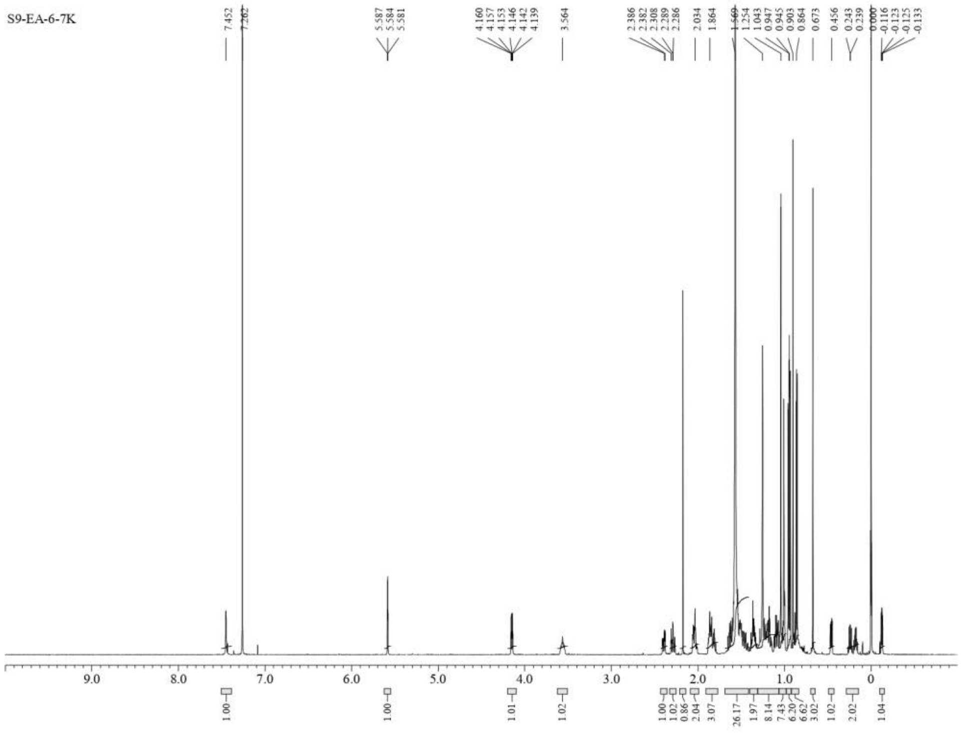

Figure S22. ^{1}H NMR spectrum (600MHz) of compound **3** in $CDCl_3$

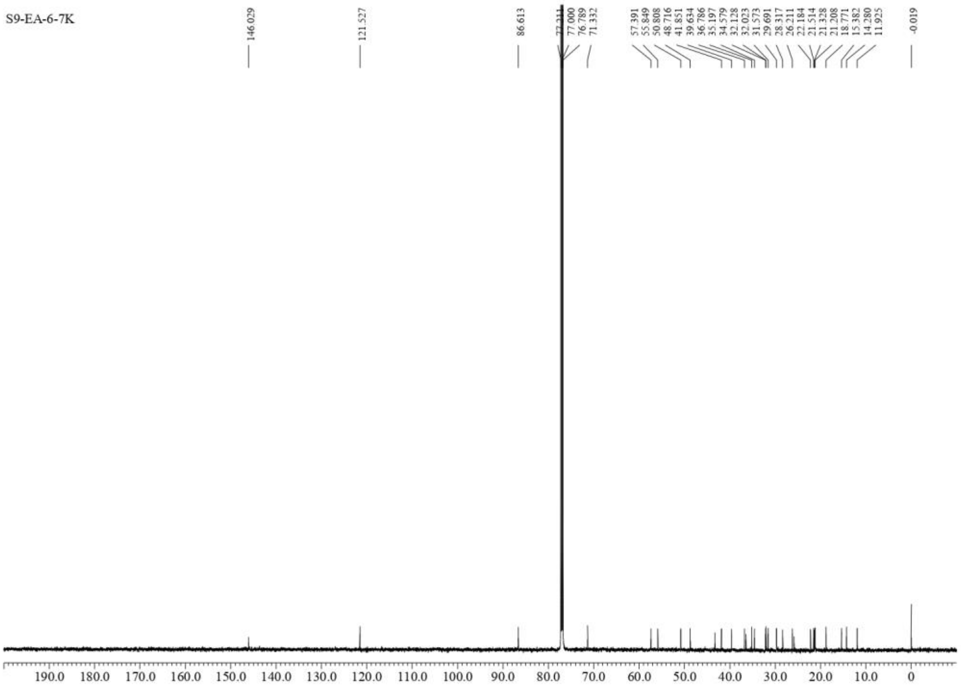

Figure S23. ^{13}C NMR spectrum (150 MHz) of compound **3** in $CDCl_3$

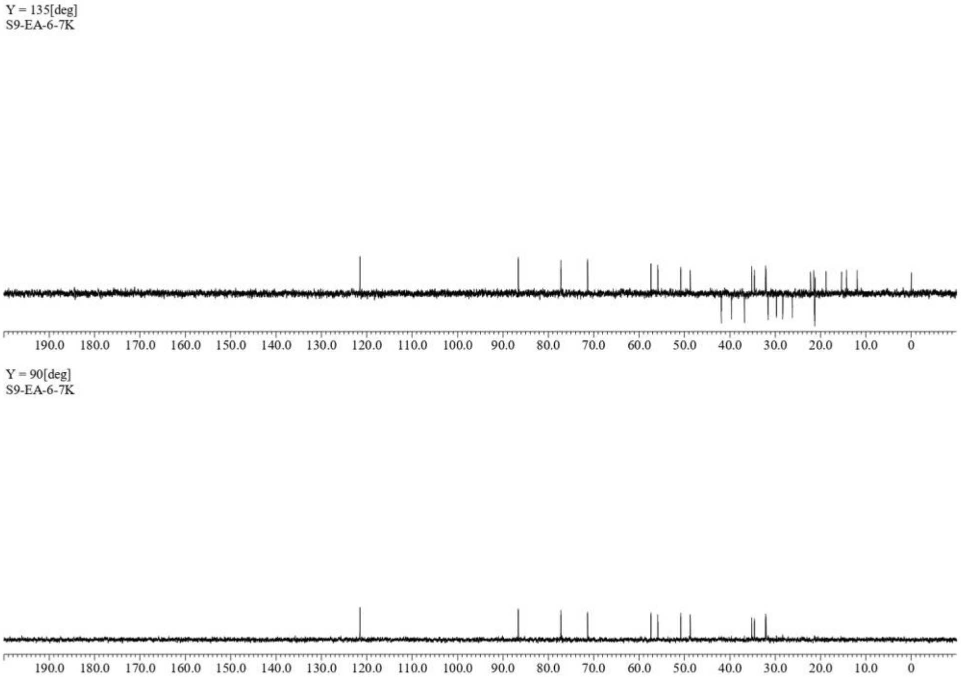

Figure S24. DEPT spectrum (150 MHz) of compound **3** in $CDCl_3$

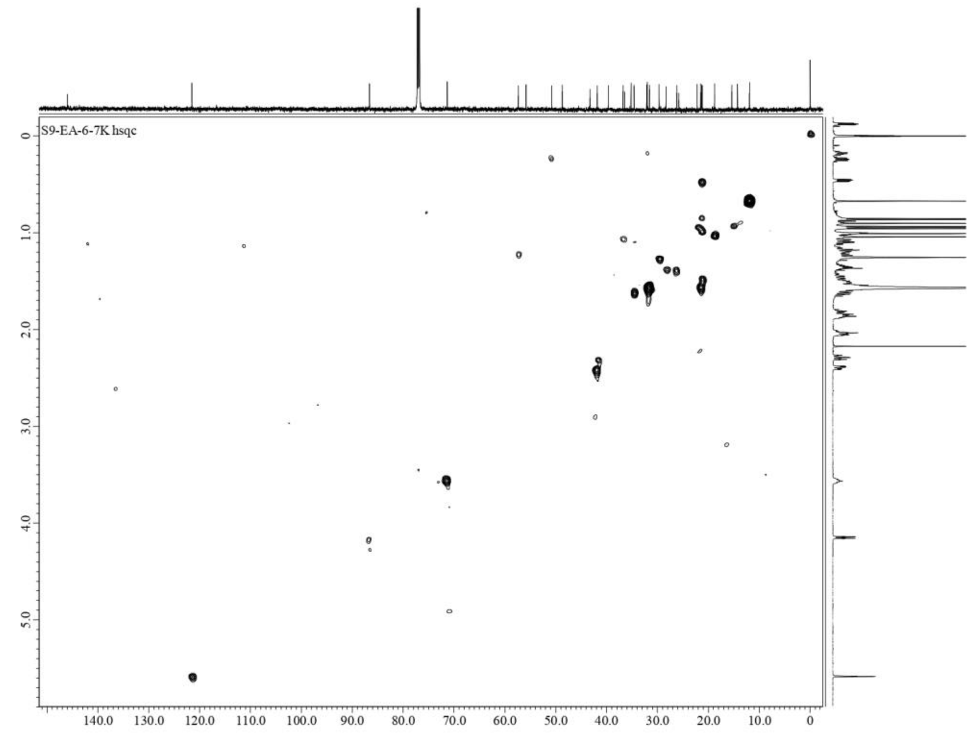

Figure S25. HSQC spectrum of compound **3** in $CDCl_3$

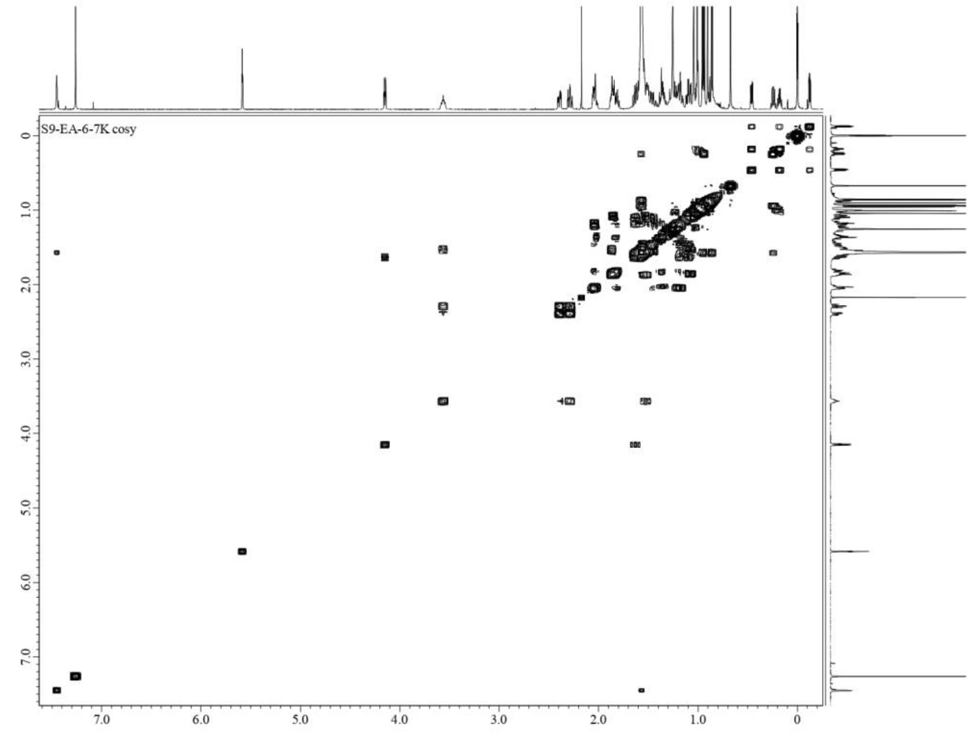

Figure S26. ^1H-^1H COSY spectrum of compound **3** in $CDCl_3$

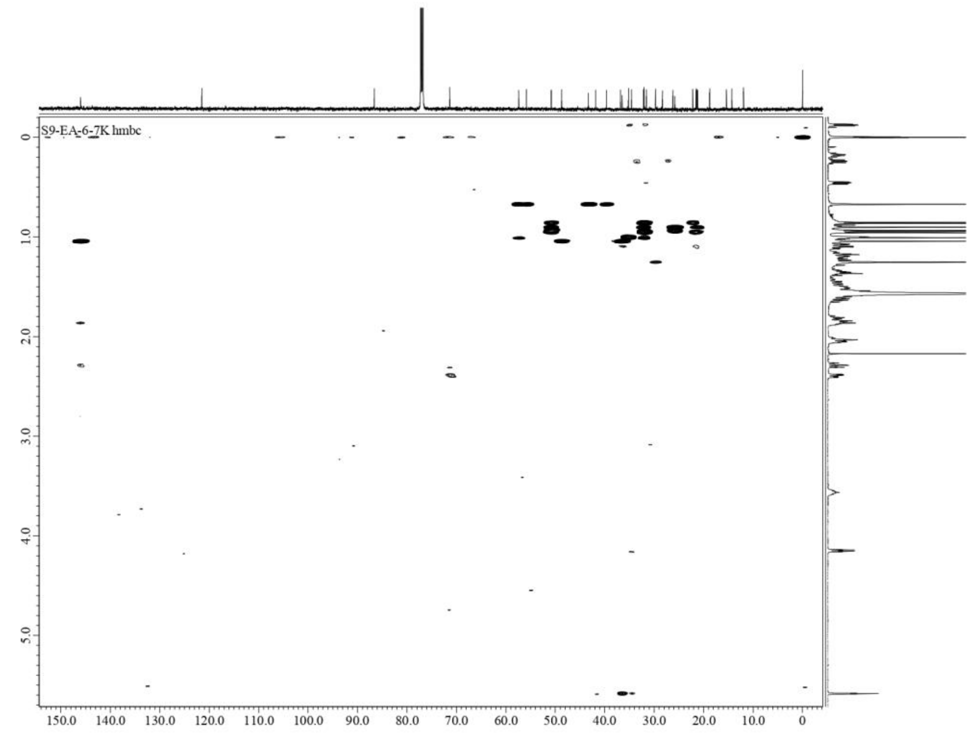

Figure S27. HMBC spectrum of compound **3** in $CDCl_3$

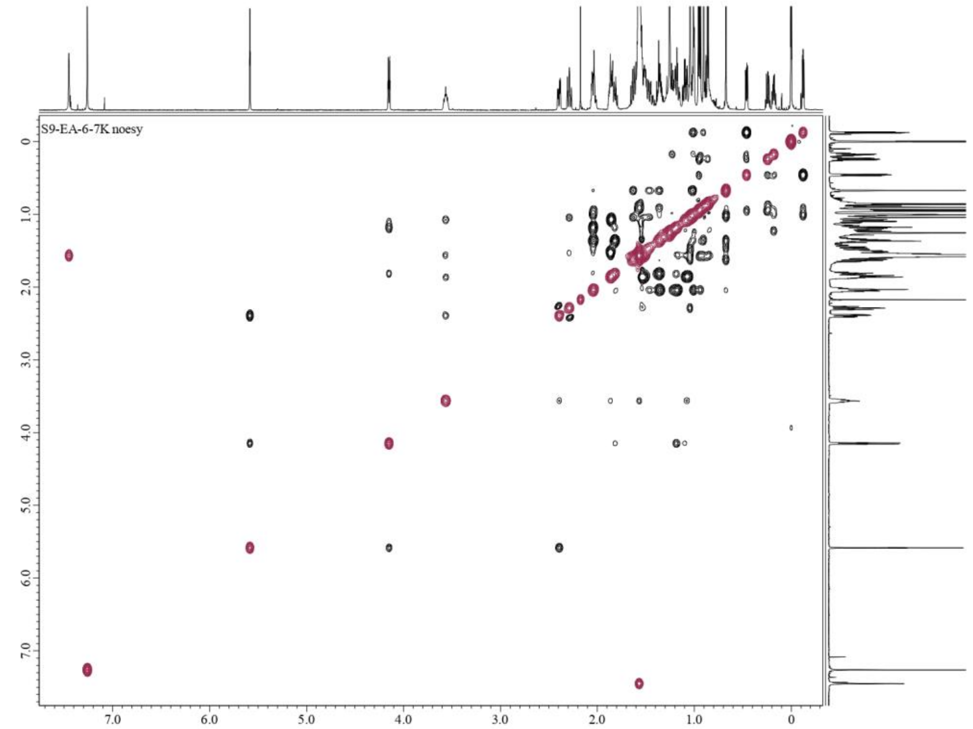

Figure S28. NOESY spectrum of compound **3** in $CDCl_3$

Analysis Info

Analysis Name	D:\Data\a1\S9EA67J_000003.d	3/26/2021 11:49:41 AM
Method	broadband first signal	Operator: YU HSIAO-CHING
Sample Name	S9-EA-6-7-J	Instrument: BRUKER FT-MS solariX
Comment	ESI Positive	

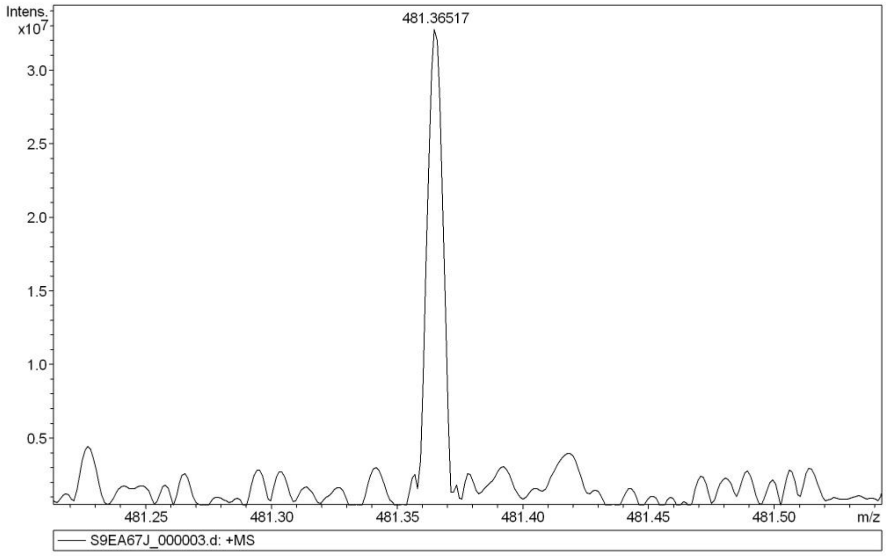

Meas. m/z	#	Formula	Score	m/z	err [mDa]	err [ppm]	mSigma	rdb	e⁻ Conf	N-Rule
481.36517	1	C 30 H 50 Na O 3	100.00	481.36522	0.05	0.10	37.5	5.5	even	ok

Figure S29. HRESIMS of compound **4**

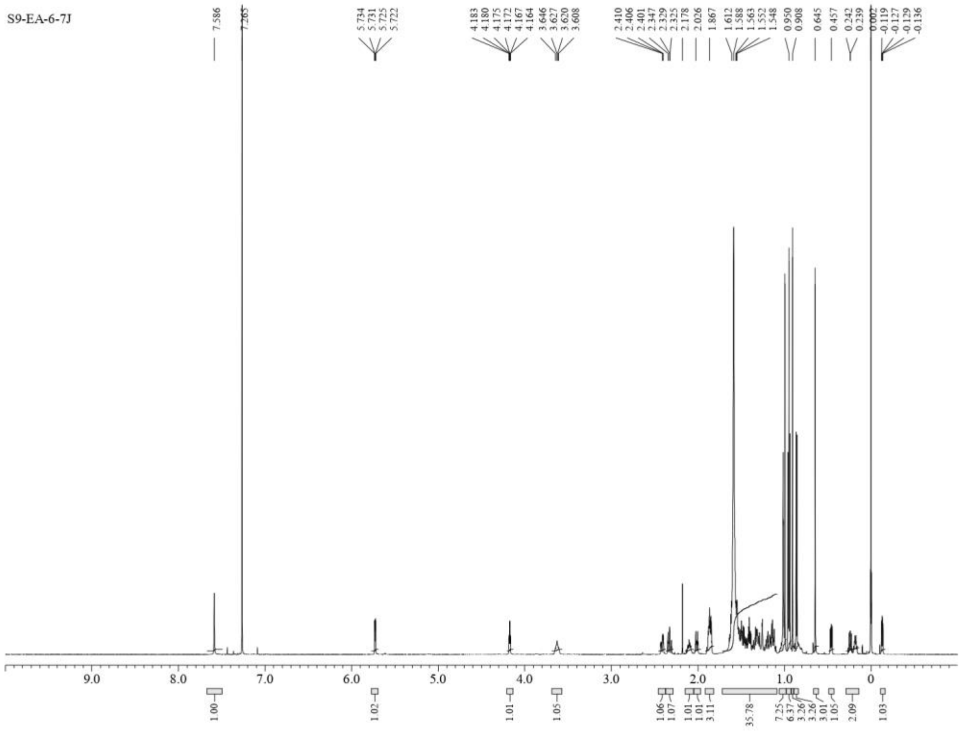

Figure S30. ^{1}H NMR spectrum (600MHz) of compound **4** in $CDCl_3$

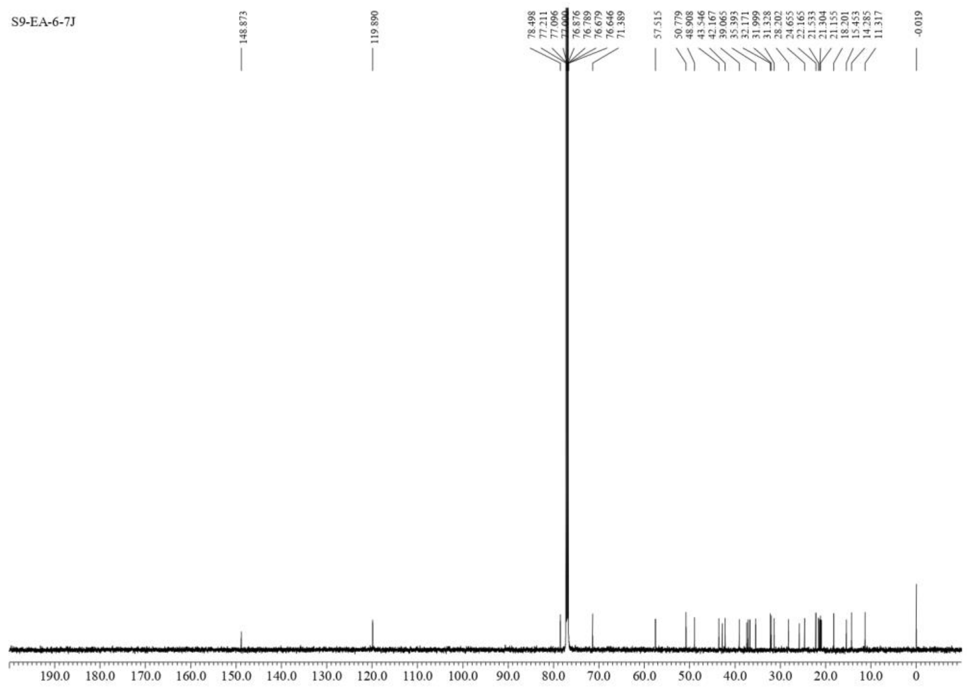

Figure S31. ^{13}C NMR spectrum (150 MHz) of compound **4** in $CDCl_3$

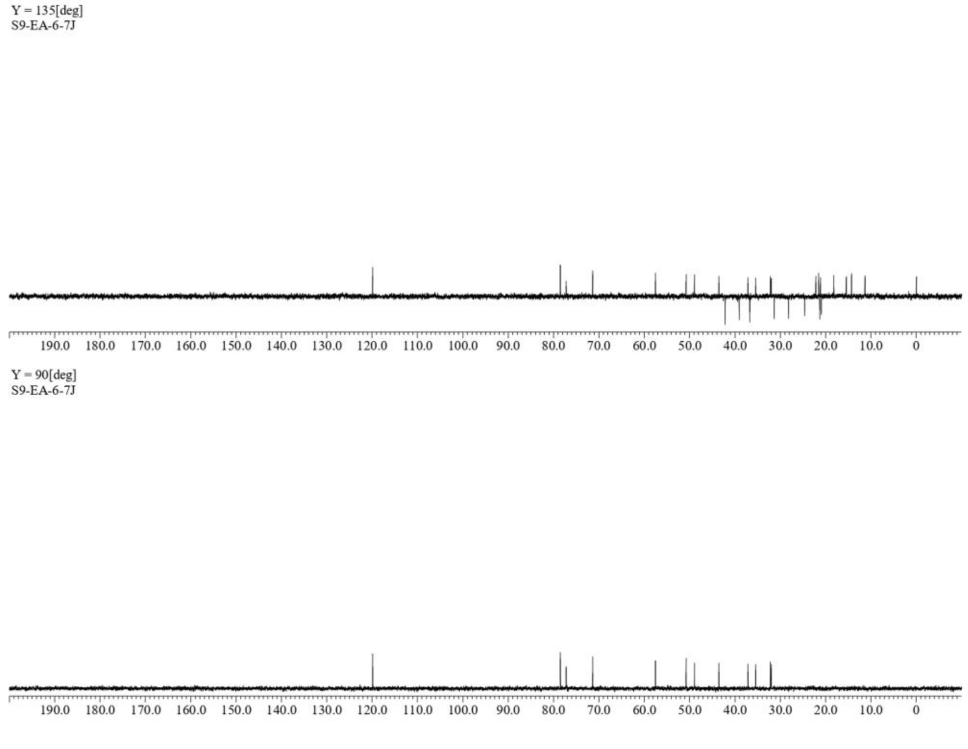

Figure S32. DEPT spectrum (150 MHz) of compound **4** in $CDCl_3$

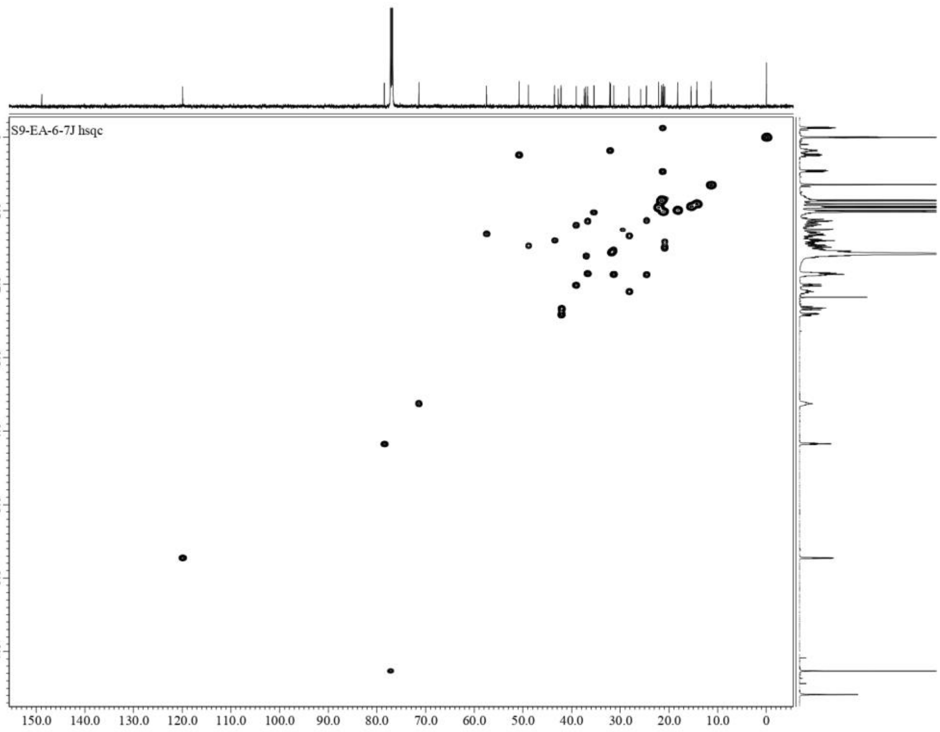

Figure S33. HSQC spectrum of compound **4** in $CDCl_3$

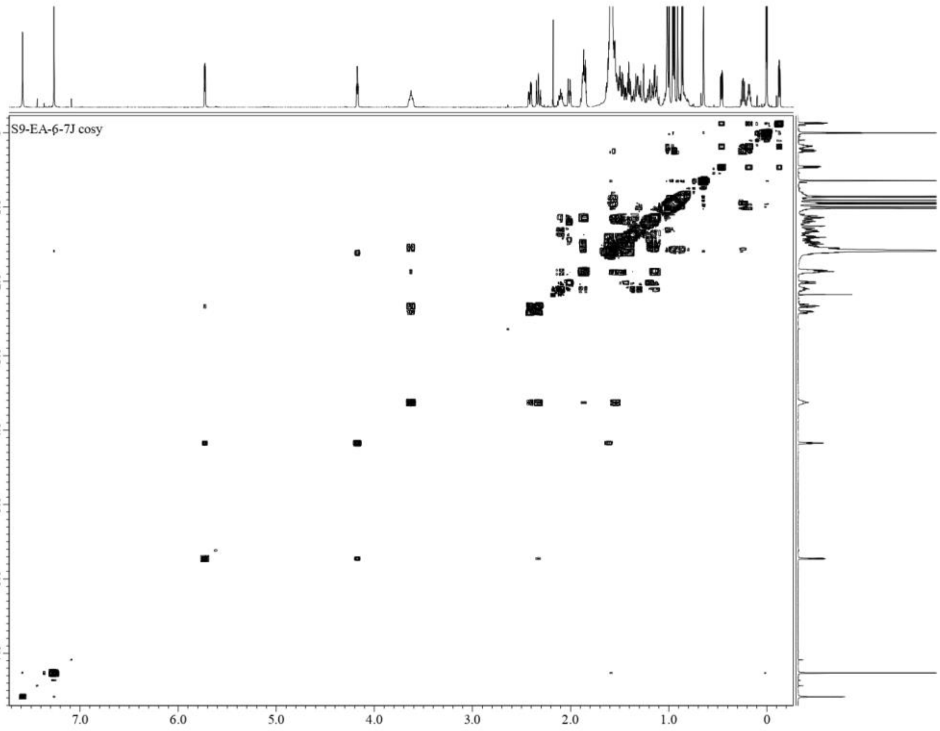

Figure S34. ^1H-^1H COSY spectrum of compound **4** in $CDCl_3$

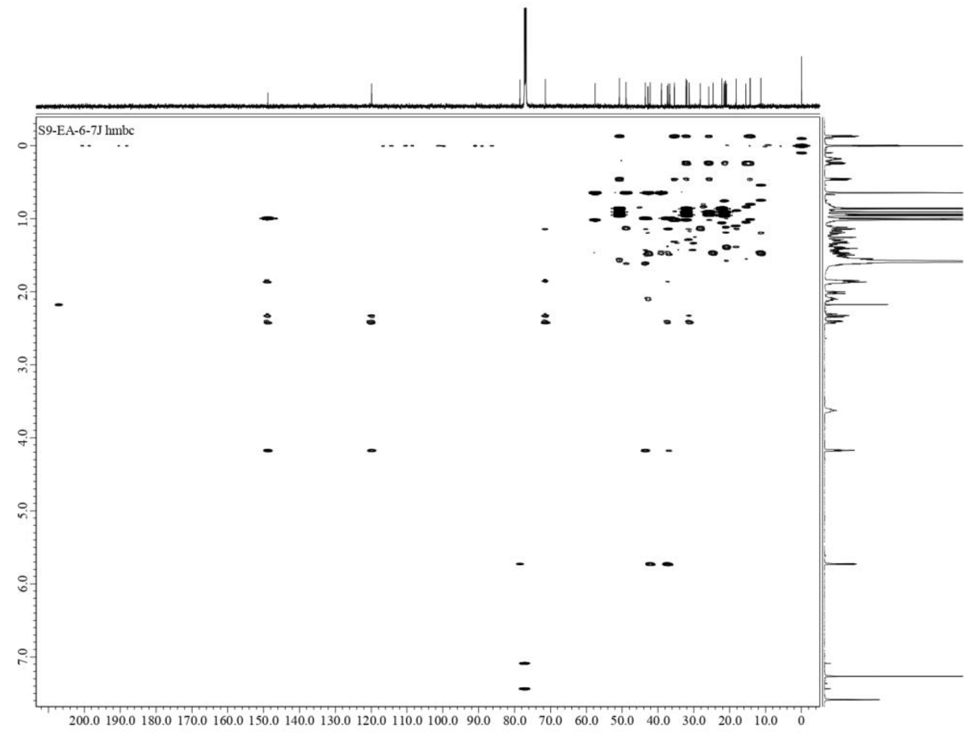

Figure S35. HMBC spectrum of compound **4** in $CDCl_3$

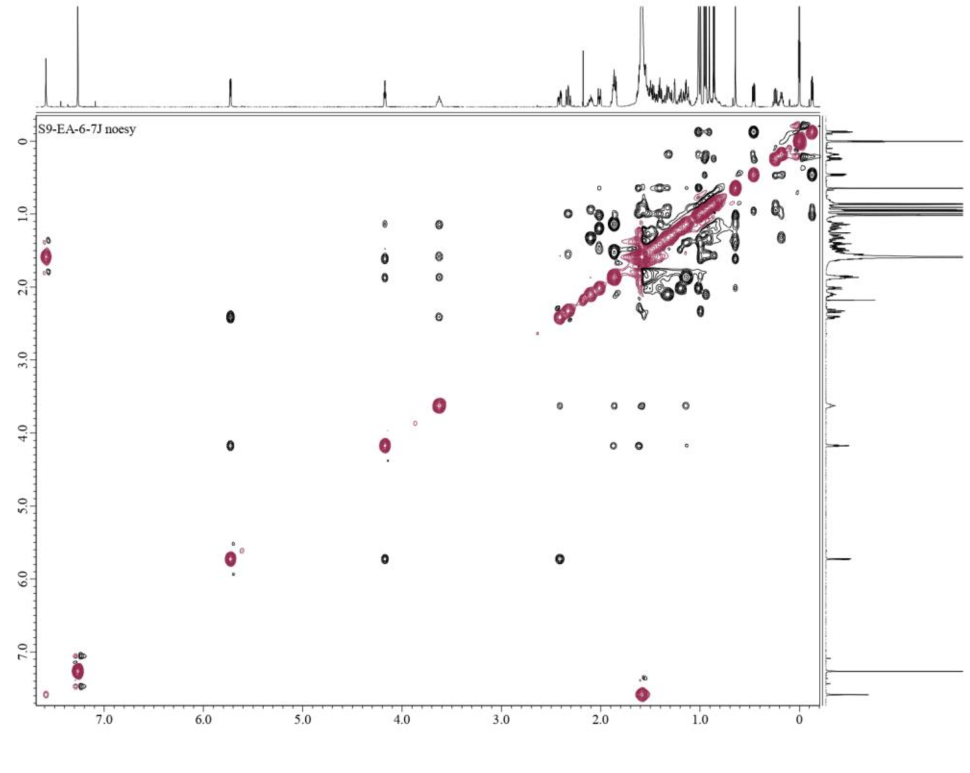

Figure S36. NOESY spectrum of compound **4** in $CDCl_3$

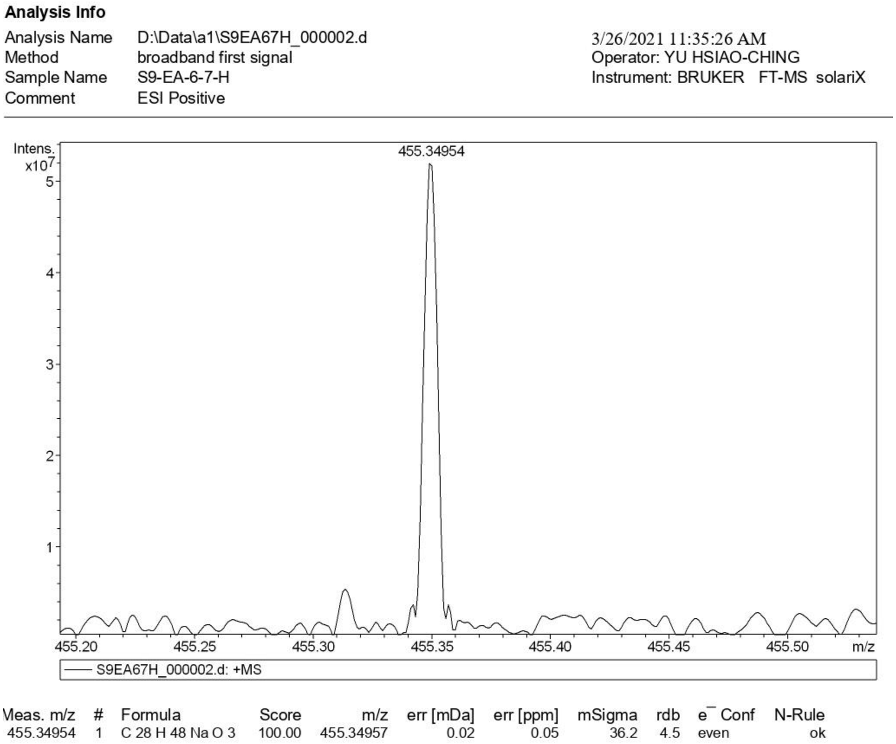

Figure S37. HRESIMS of compound **5**

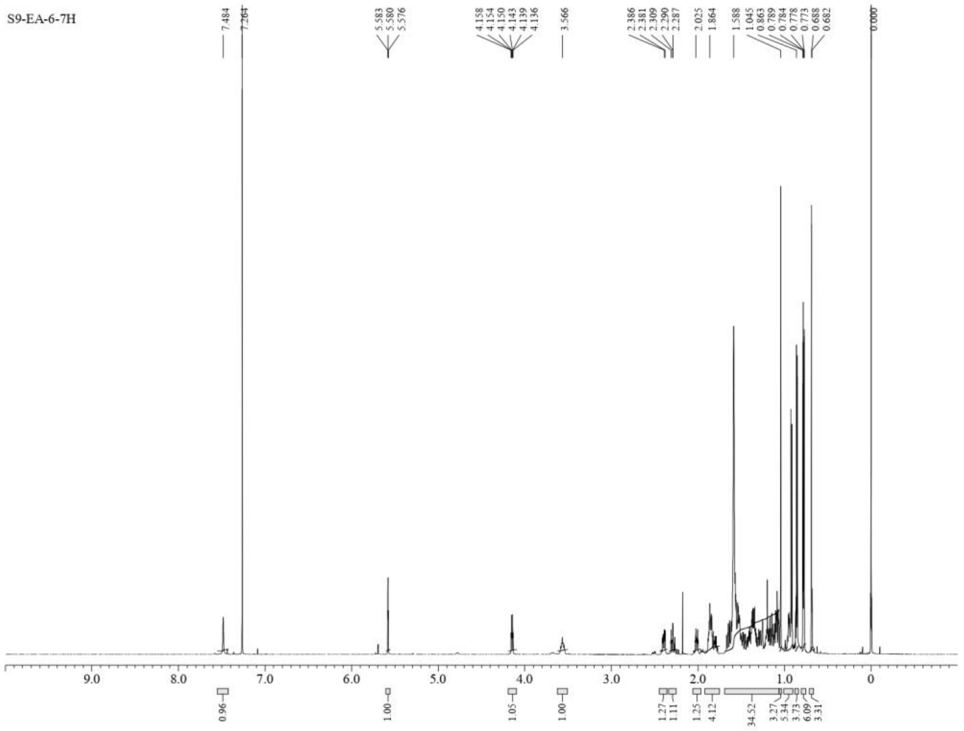

Figure S38. ^{1}H NMR spectrum (600MHz) of compound **5** in $CDCl_3$

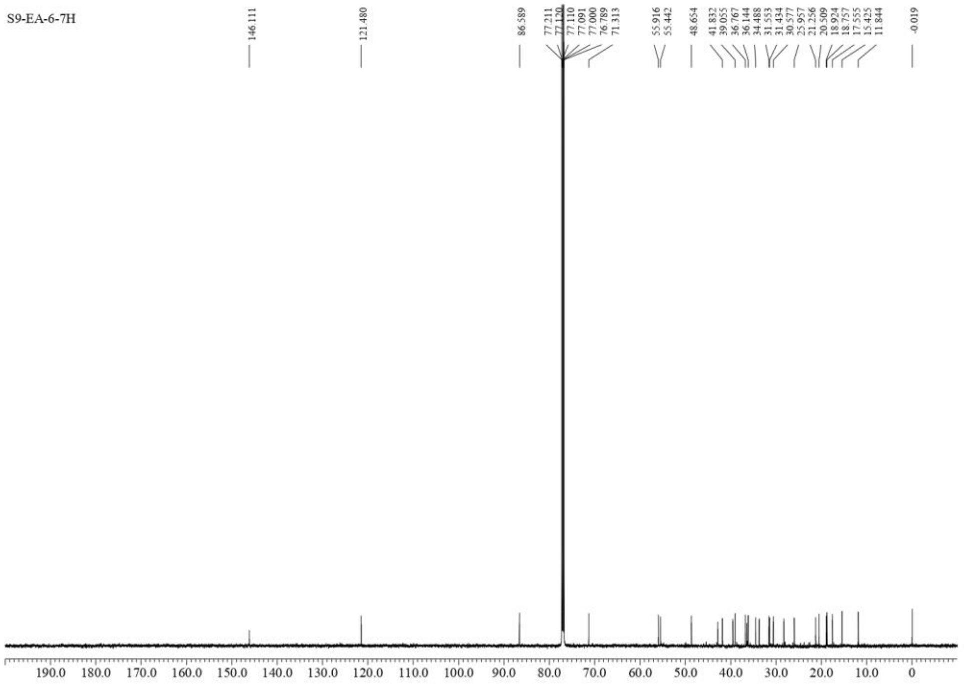

Figure S39. ^{13}C NMR spectrum (150 MHz) of compound **5** in $CDCl_3$

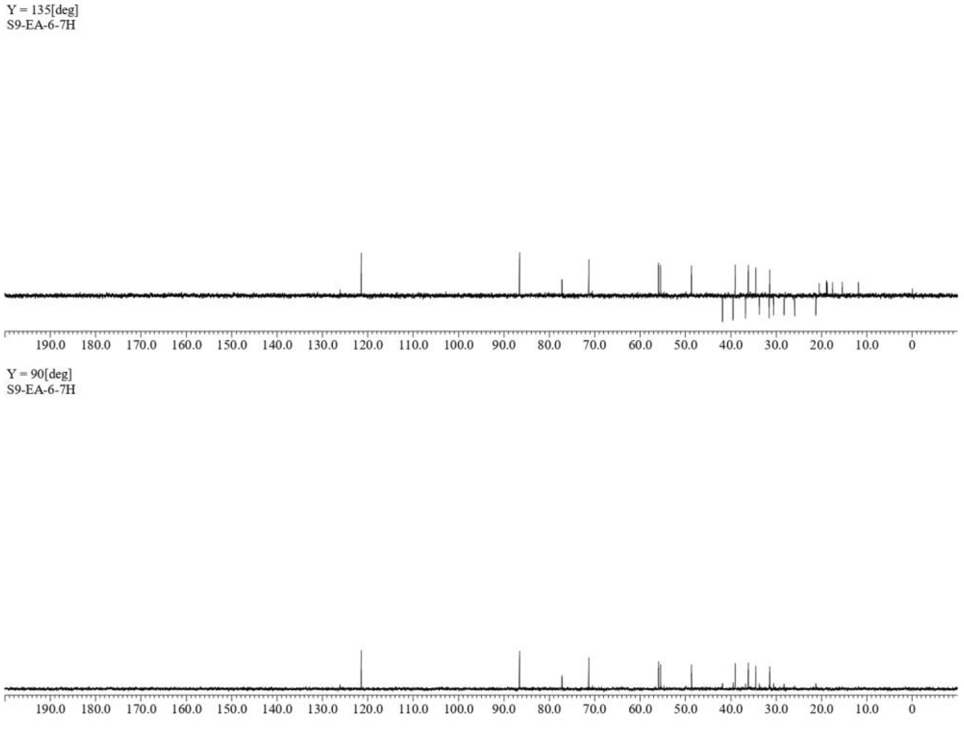

Figure S40. DEPT spectrum (150 MHz) of compound **5** in $CDCl_3$

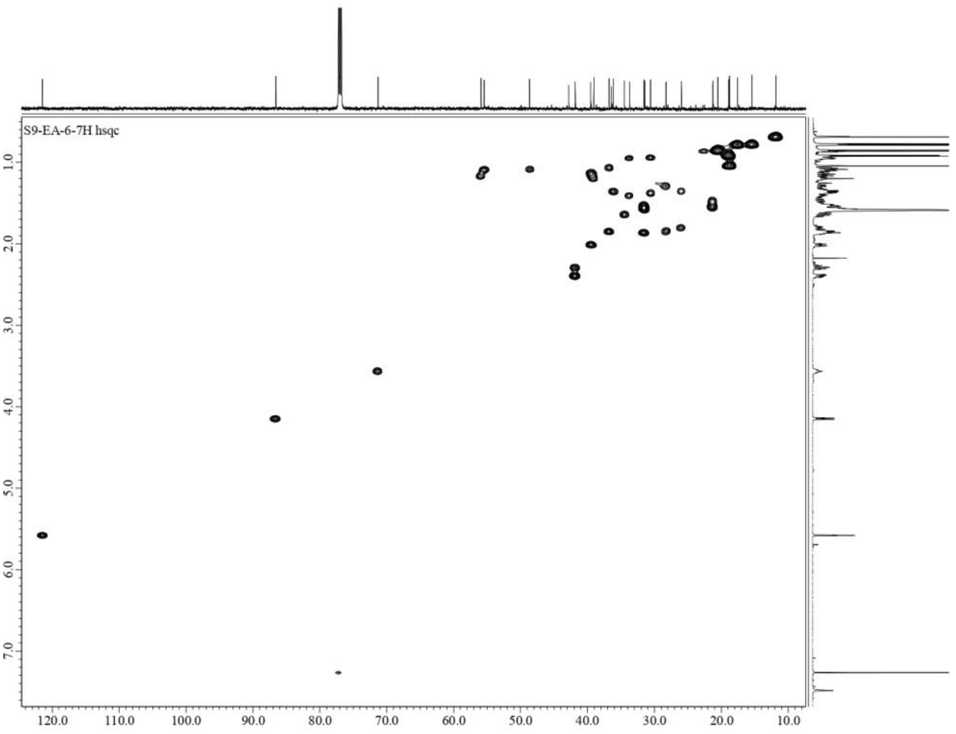

Figure S41. HSQC spectrum of compound **5** in $CDCl_3$

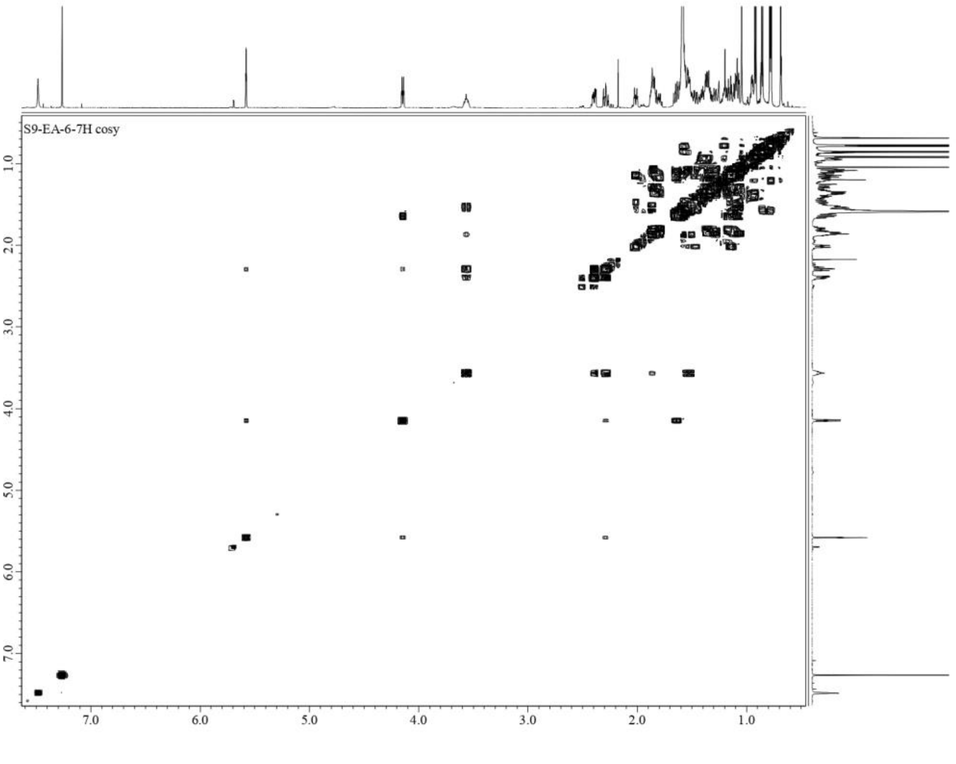

Figure S42. ^1H-^1H COSY spectrum of compound **5** in $CDCl_3$

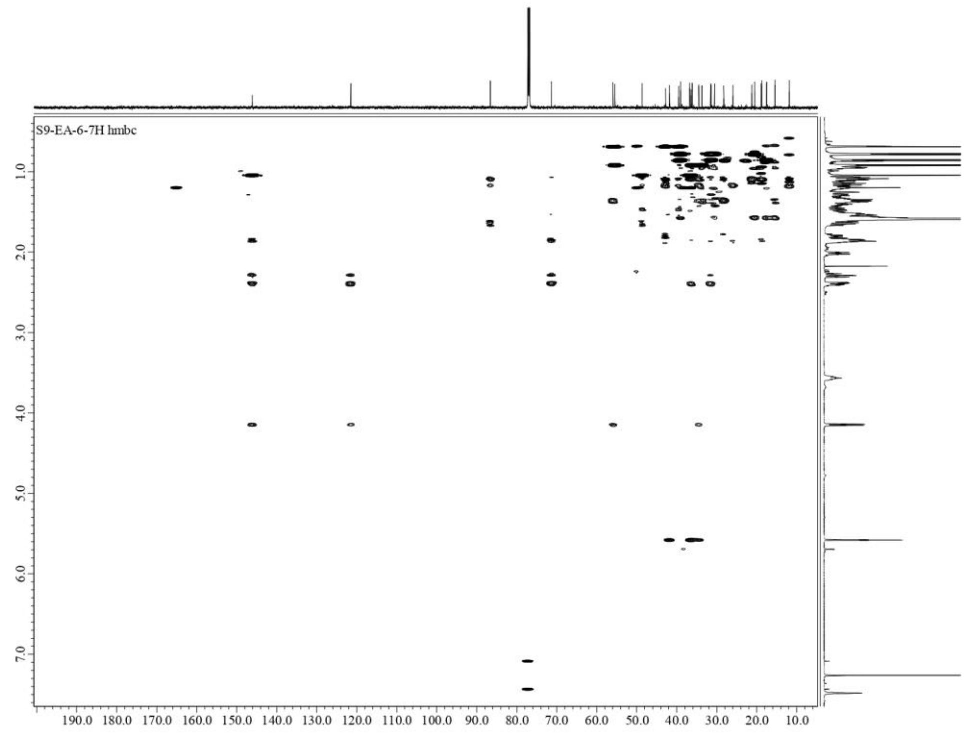

Figure S43. HMBC spectrum of compound **5** in $CDCl_3$

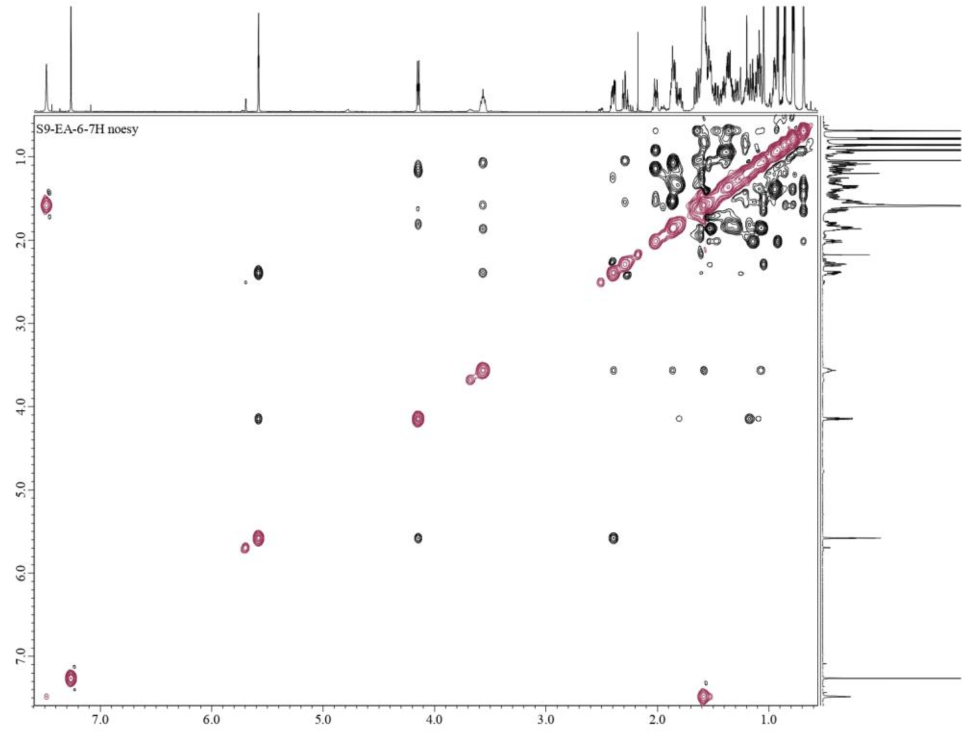

Figure S44. NOESY spectrum of compound **5** in $CDCl_3$

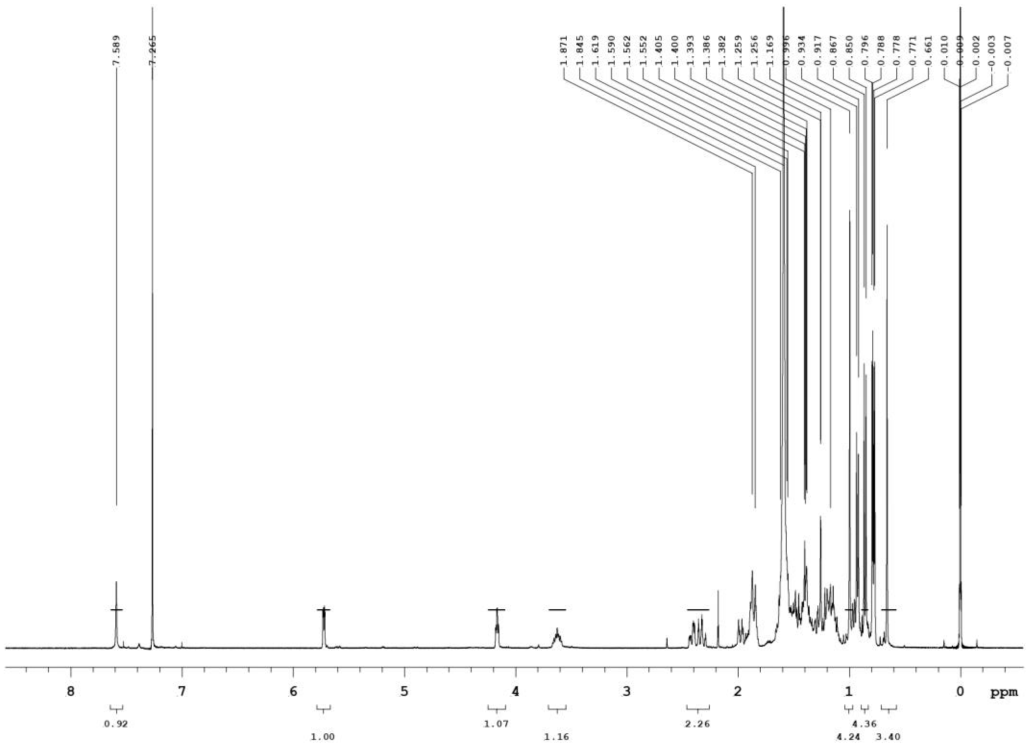

Figure S45. ^{1}H NMR spectrum (400MHz) of compound **6** in $CDCl_3$

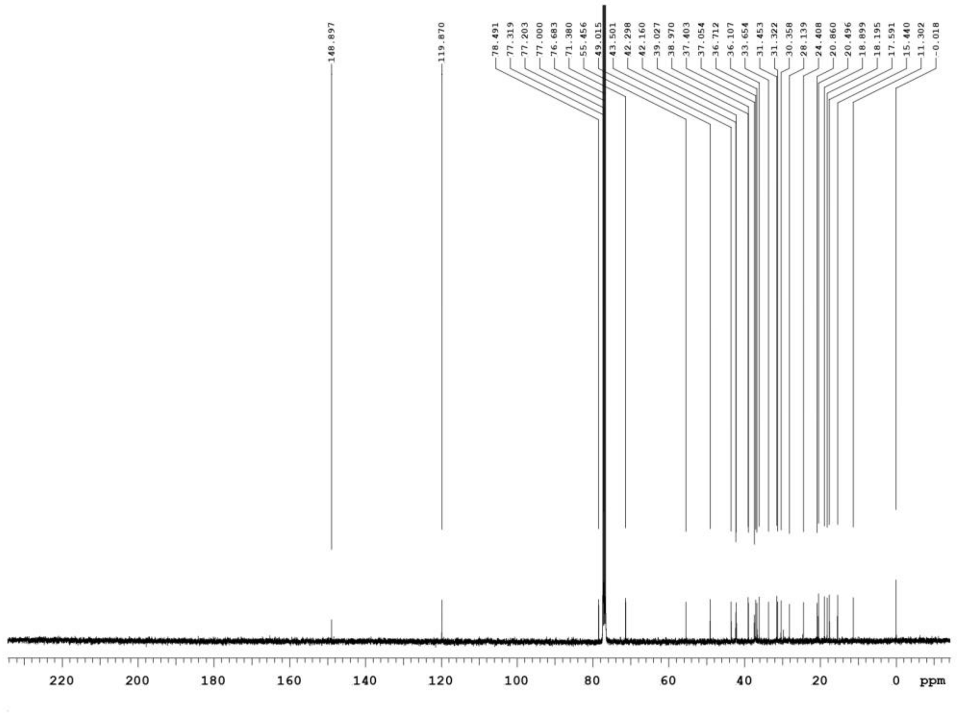

Figure S46. ^{13}C NMR spectrum (100 MHz) of compound **6** in $CDCl_3$

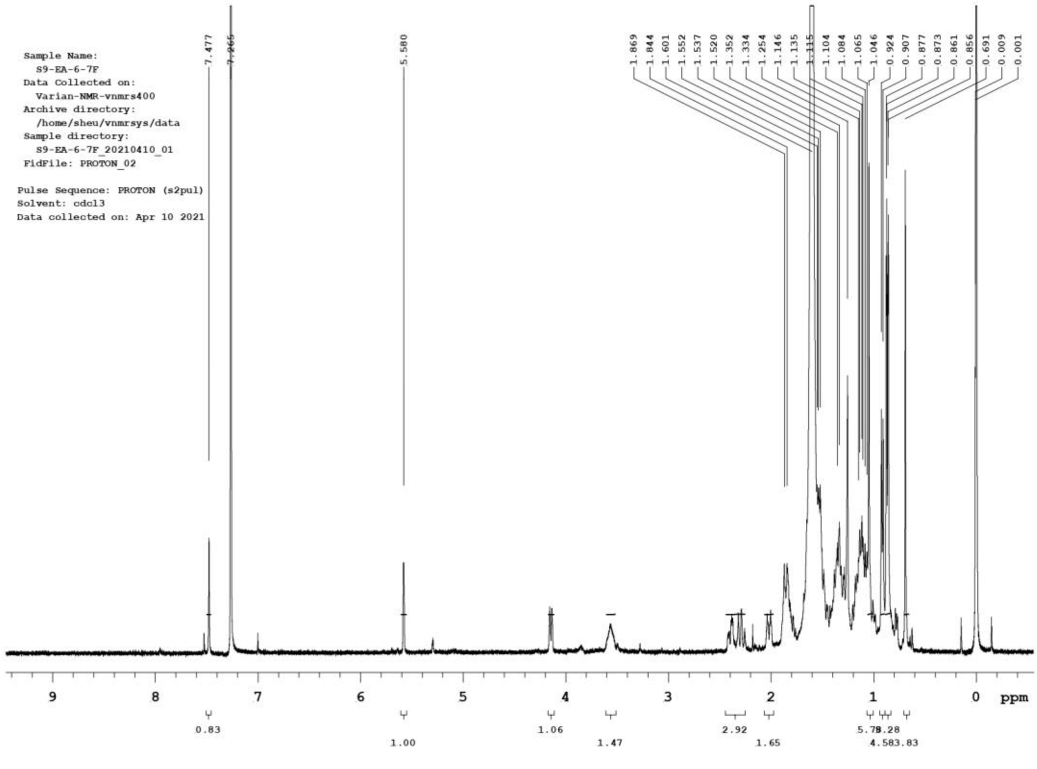

Figure S47. ^{1}H NMR spectrum (400MHz) of compound **7** in $CDCl_3$

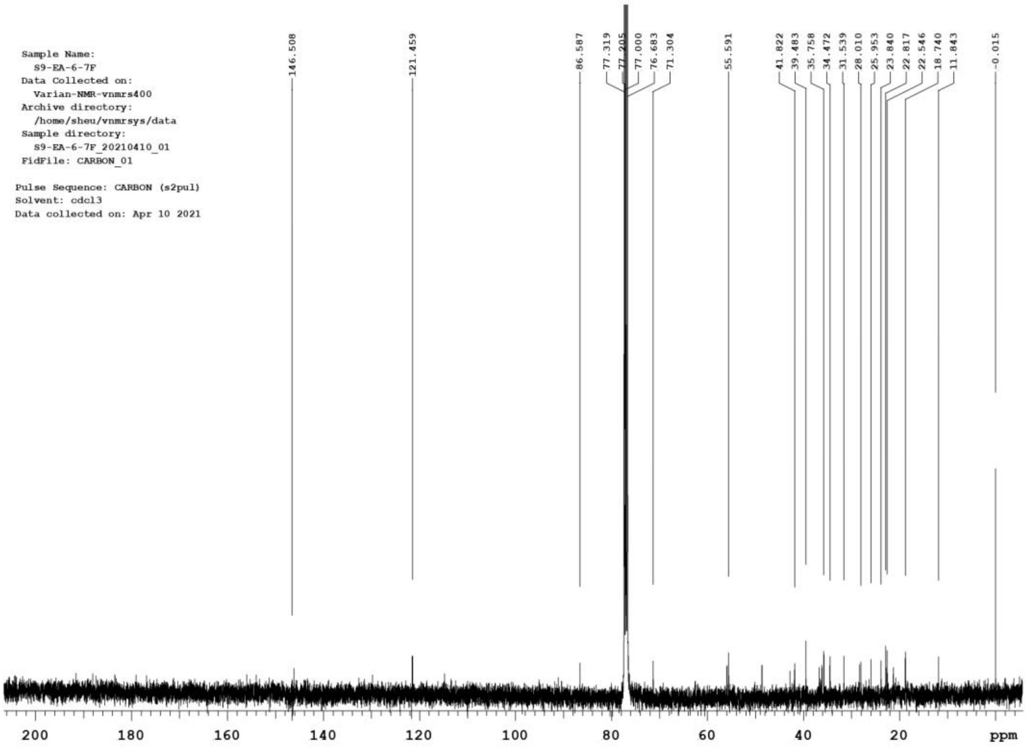

Figure S48. ^{13}C NMR spectrum (100 MHz) of compound **7** in $CDCl_3$

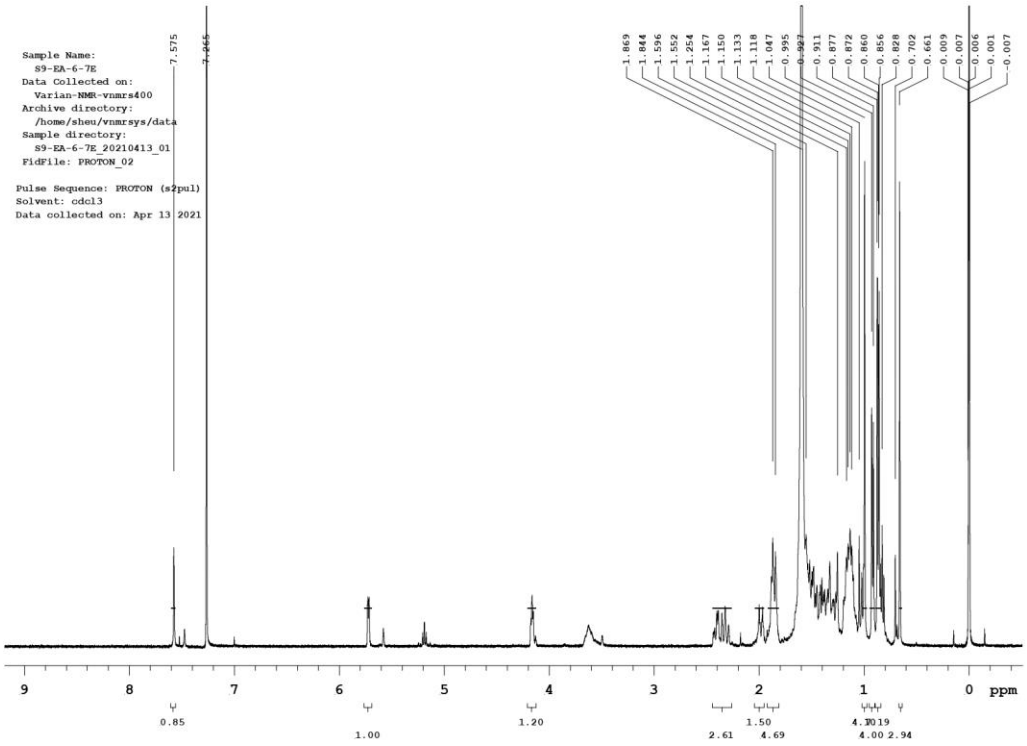

Figure S49. ^{1}H NMR spectrum (400MHz) of compound **8** in $CDCl_3$

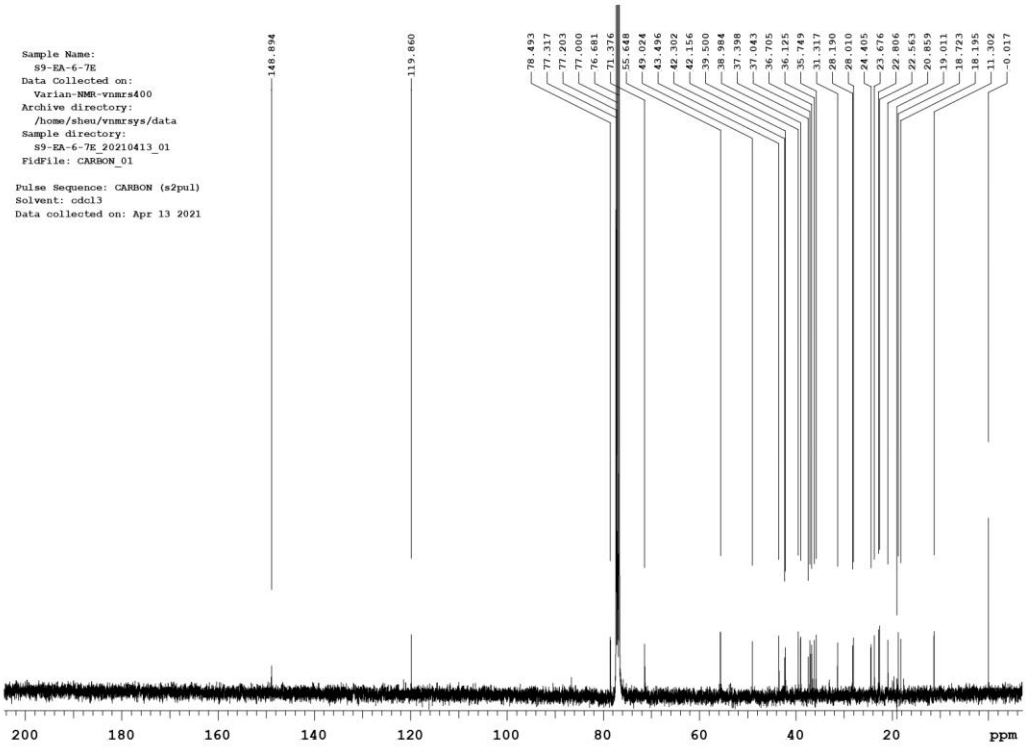

Figure S50. ^{13}C NMR spectrum (100 MHz) of compound **8** in $CDCl_3$

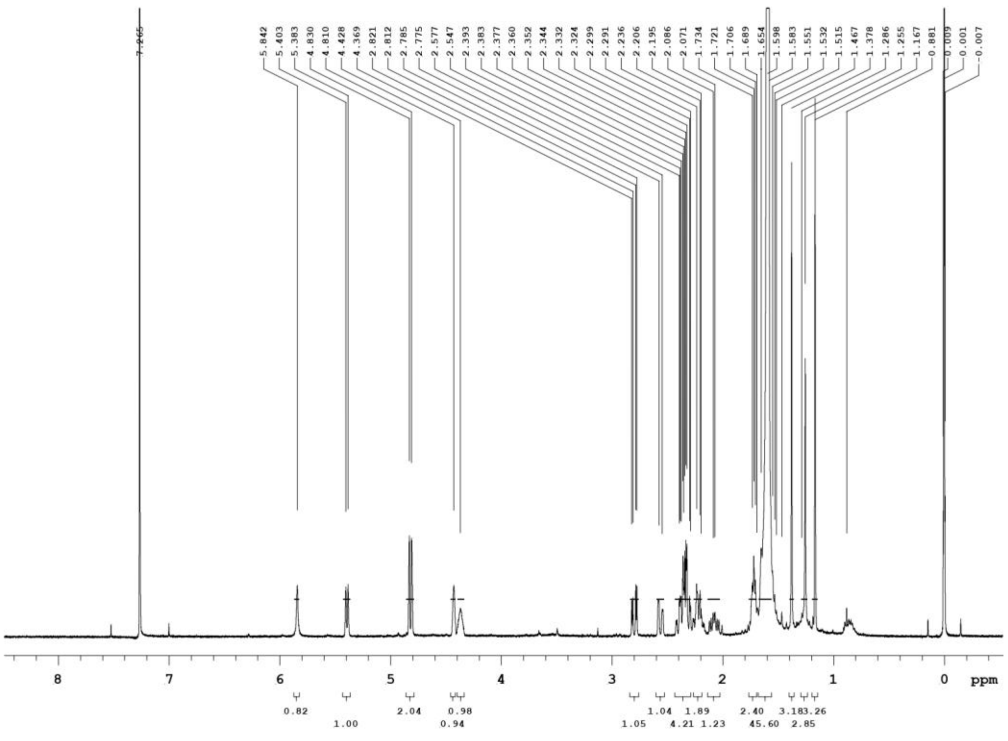

Figure S51. ^{1}H NMR spectrum (400MHz) of compound **9** in $CDCl_3$

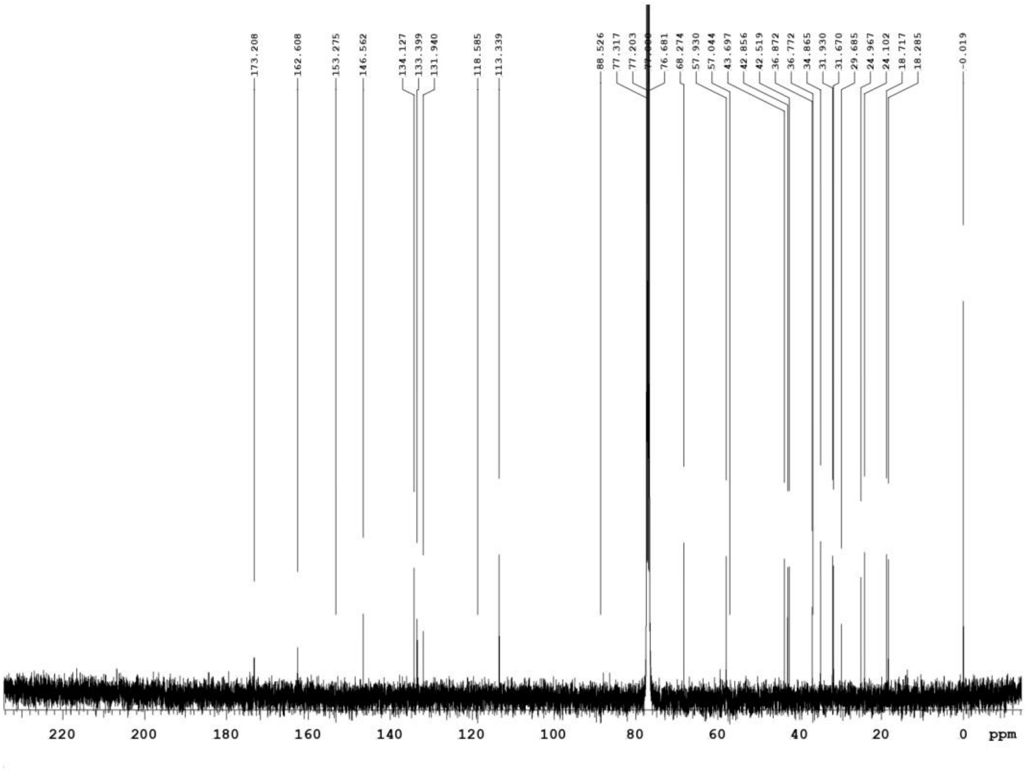

Figure S52. ^{13}C NMR spectrum (100 MHz) of compound **9** in $CDCl_3$

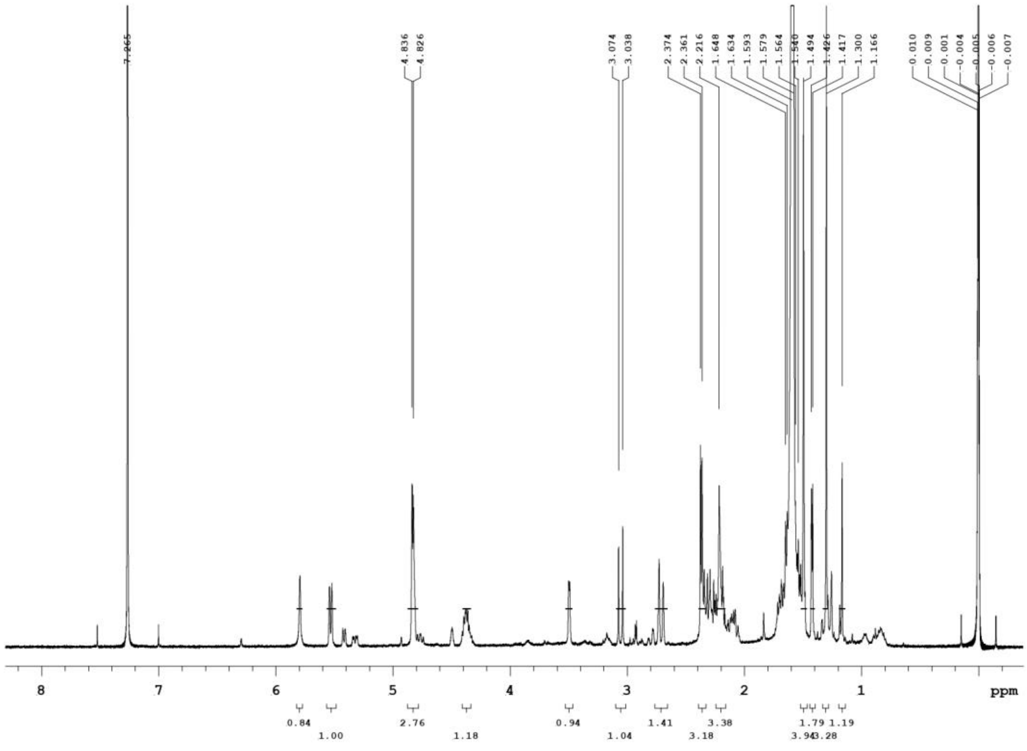

Figure S53. ^{1}H NMR spectrum (400MHz) of compound **10** in $CDCl_3$

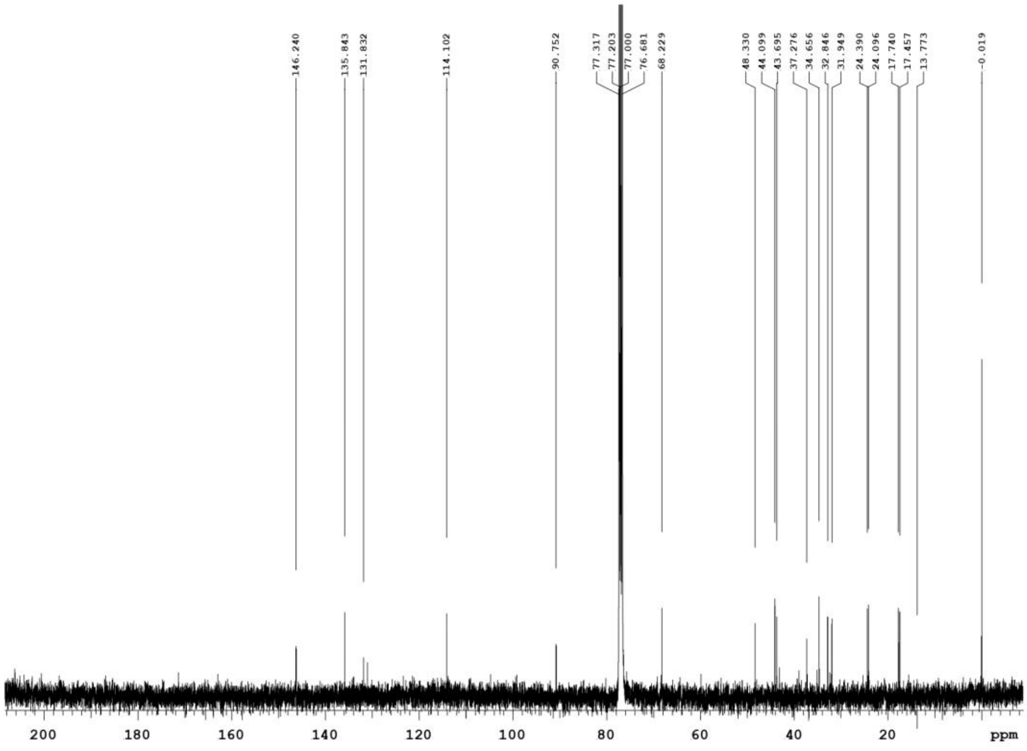

Figure S54. ^{13}C NMR spectrum (100 MHz) of compound **10** in $CDCl_3$

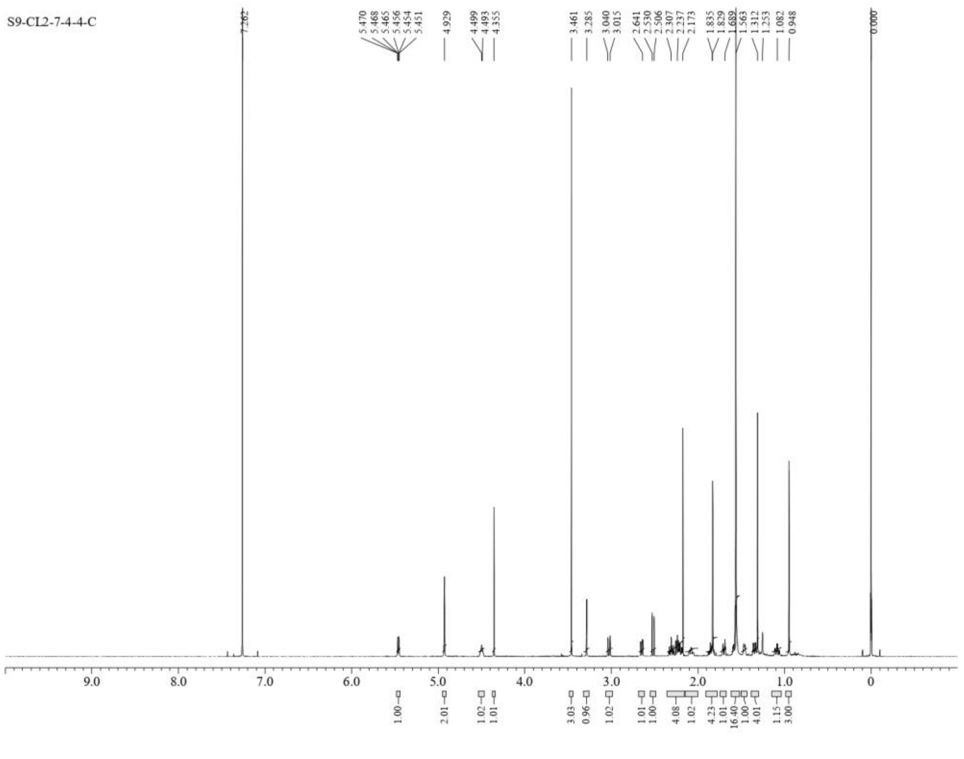

Figure S55. ^{1}H NMR spectrum (600MHz) of compound **11** in $CDCl_3$

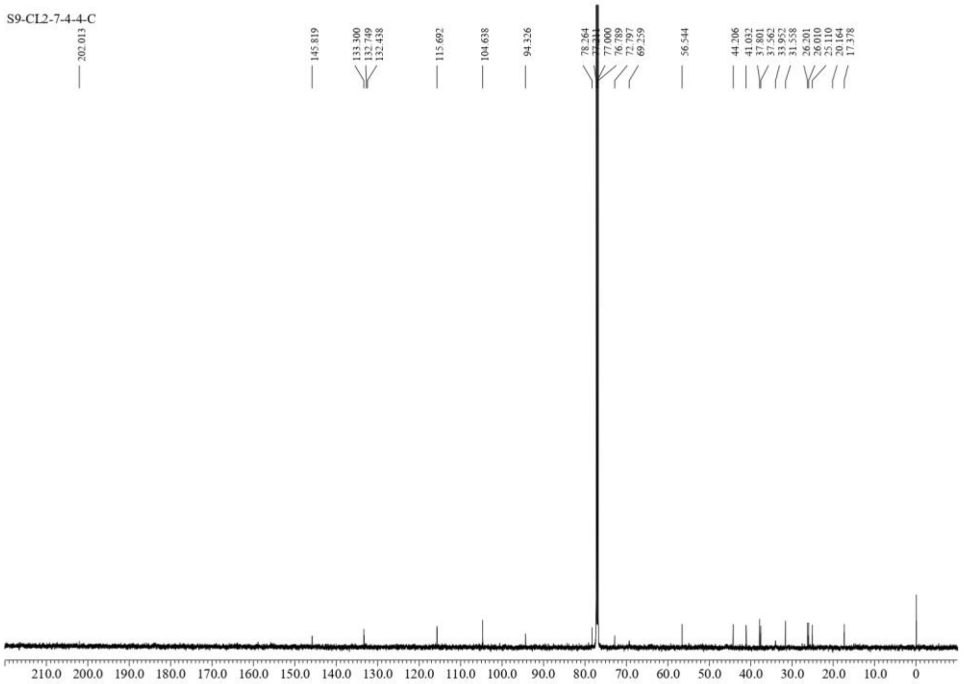

Figure S56. ^{13}C NMR spectrum (150 MHz) of compound **11** in $CDCl_3$

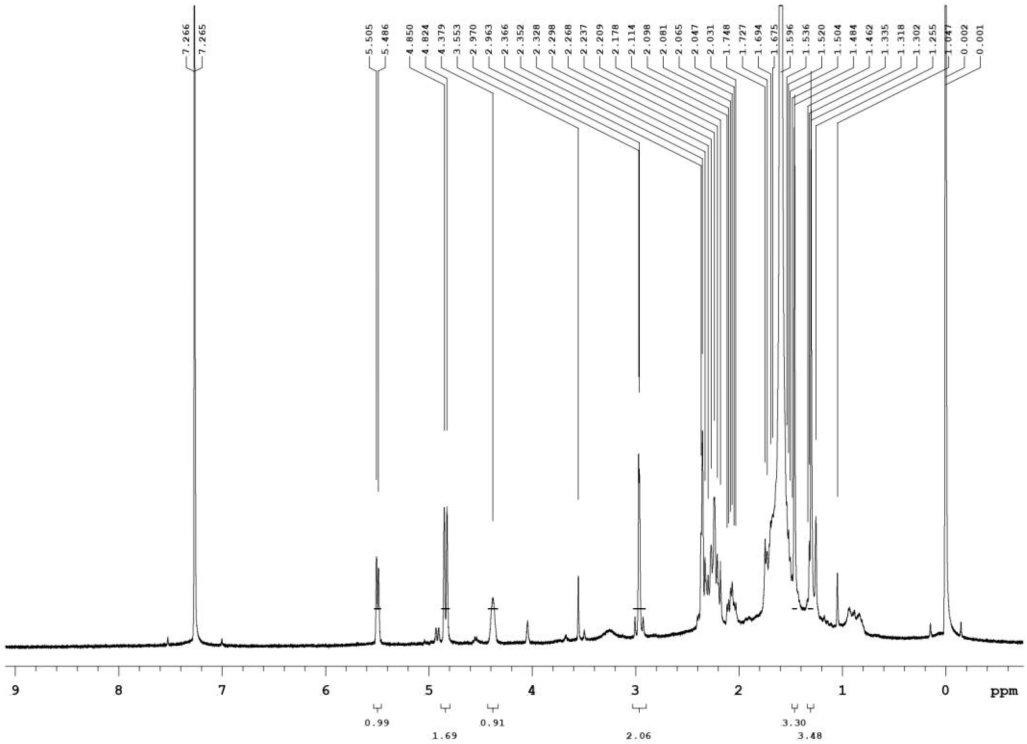

Figure S57. ^{1}H NMR spectrum (400MHz) of compound **12** in $CDCl_3$

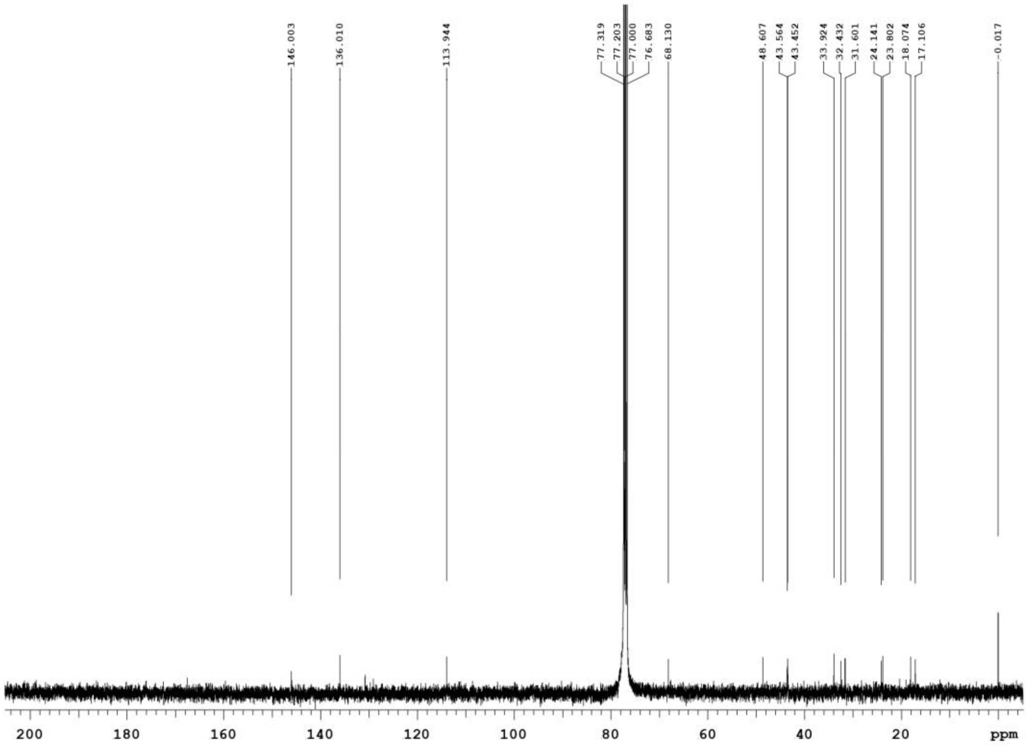

Figure S58. ^{13}C NMR spectrum (100 MHz) of compound **12** in $CDCl_3$

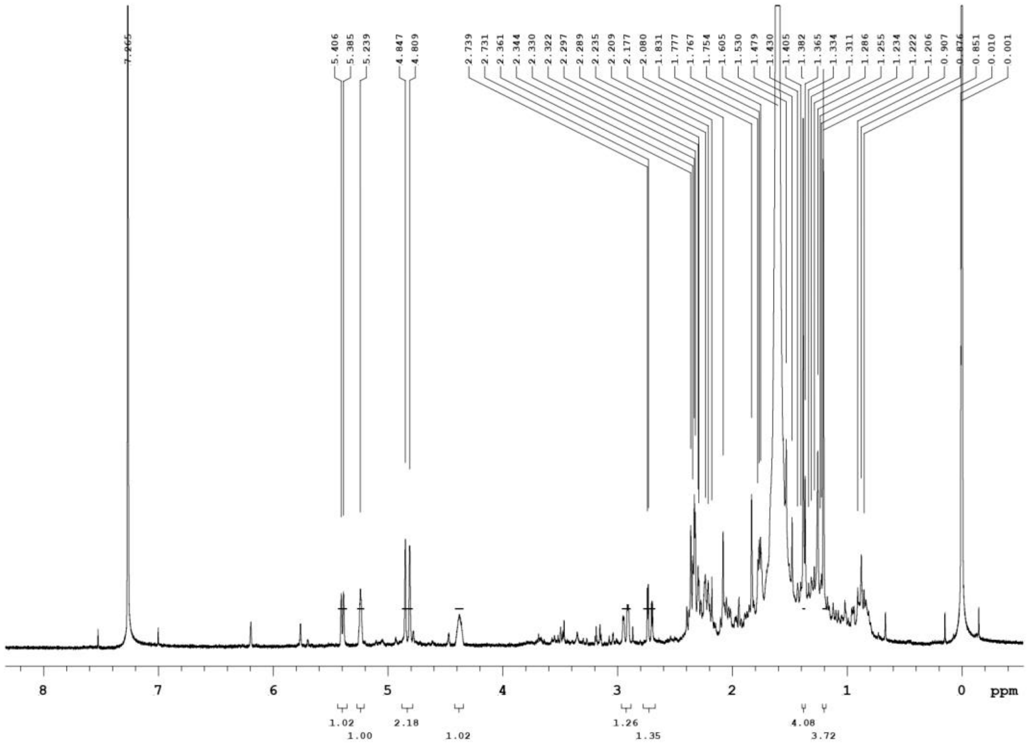

Figure S59. ^{1}H NMR spectrum (400MHz) of compound **13** in $CDCl_3$

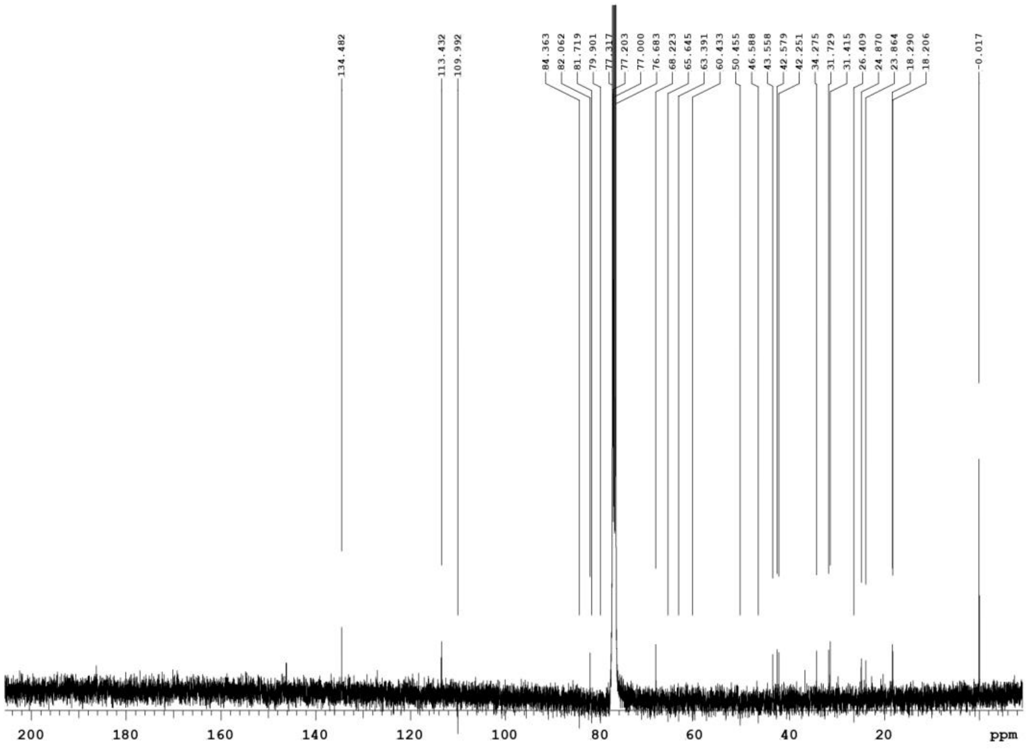

Figure S60. ^{13}C NMR spectrum (100 MHz) of compound **13** in $CDCl_3$

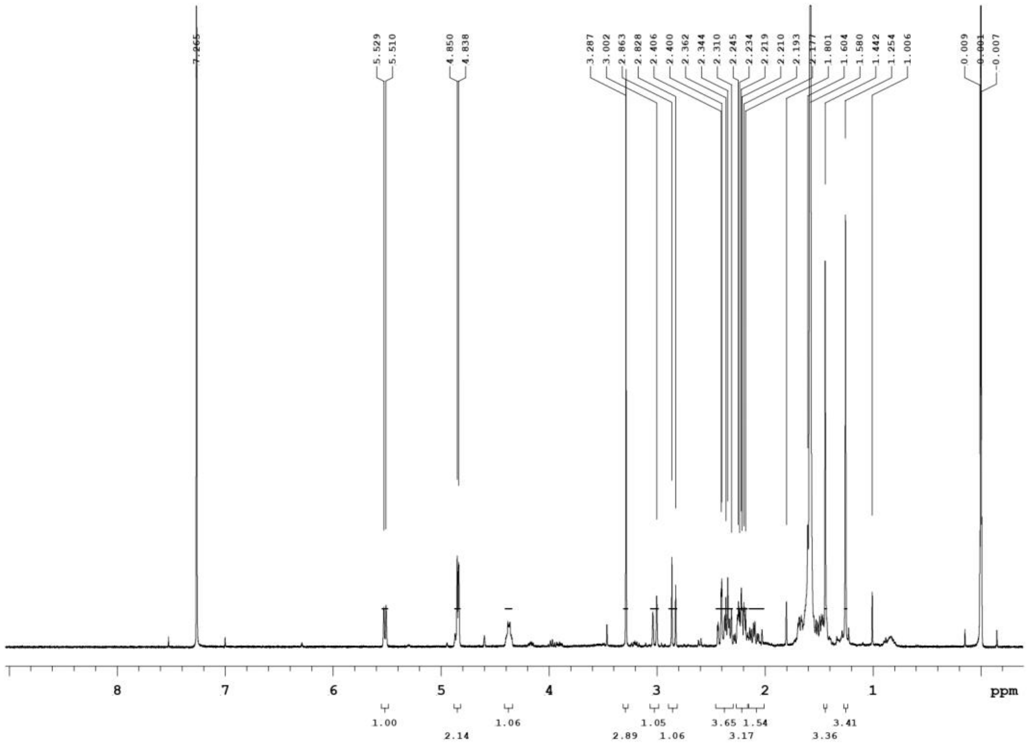

Figure S61. ^{1}H NMR spectrum (400MHz) of compound **14** in $CDCl_3$

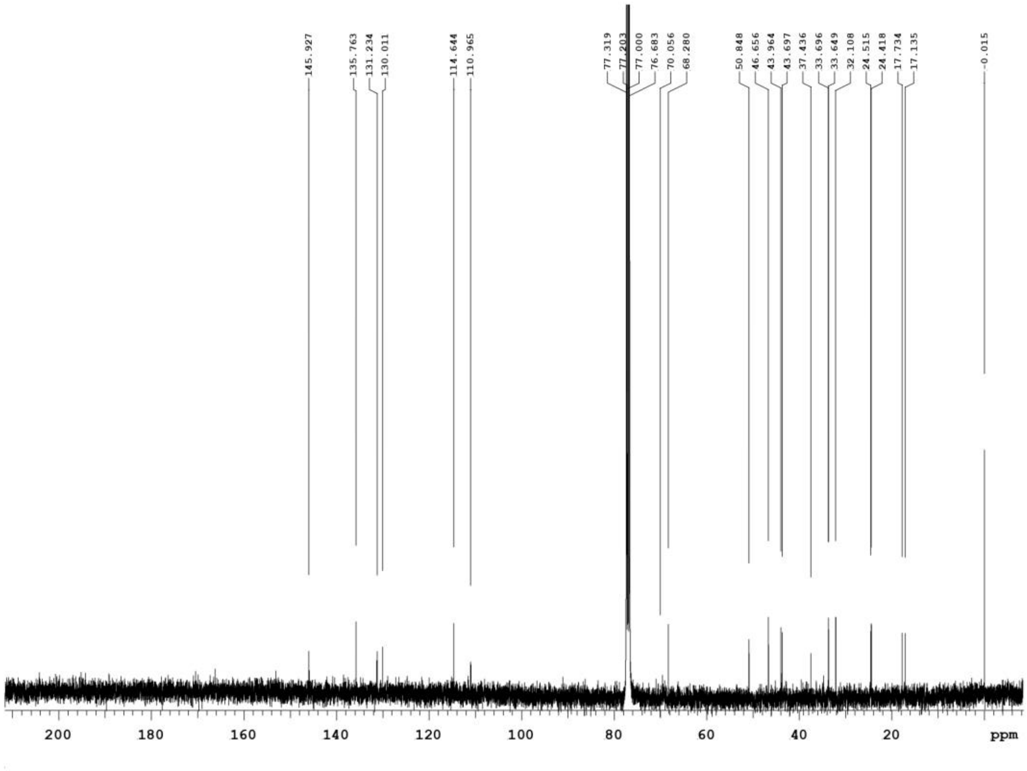

Figure S62. ^{13}C NMR spectrum (100 MHz) of compound **14** in $CDCl_3$

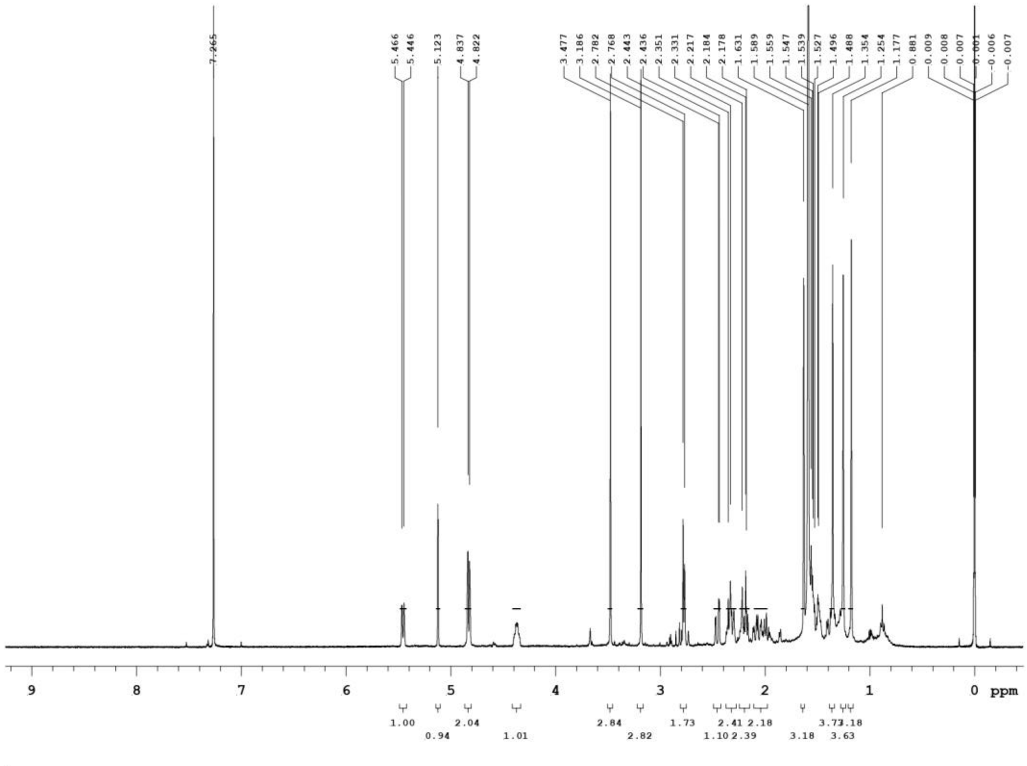

Figure S63. ^{1}H NMR spectrum (400MHz) of compound **15** in $CDCl_3$

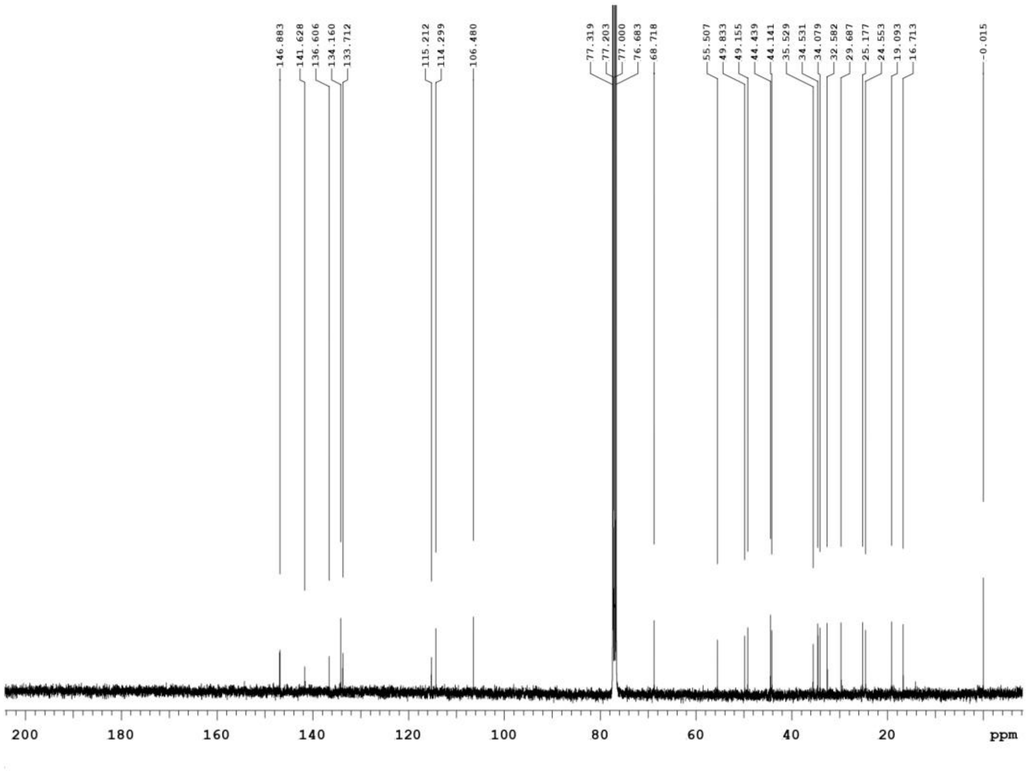

Figure S64. ^{13}C NMR spectrum (100 MHz) of compound **15** in $CDCl_3$

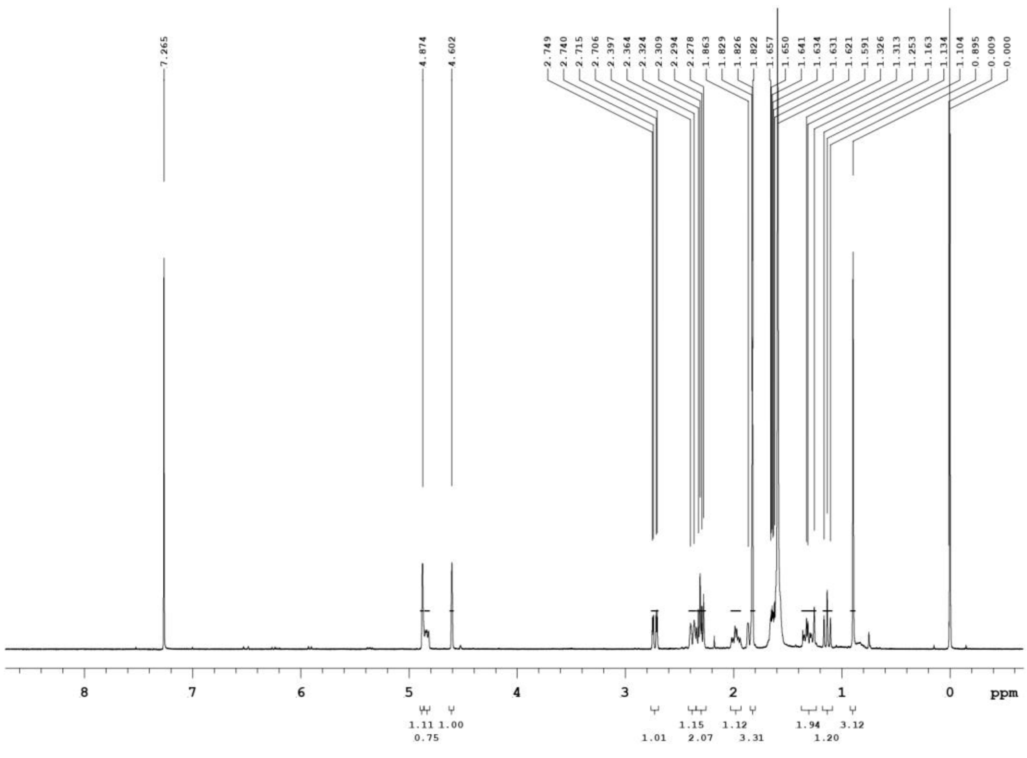

Figure S65. ^{1}H NMR spectrum (400MHz) of compound **16** in $CDCl_3$

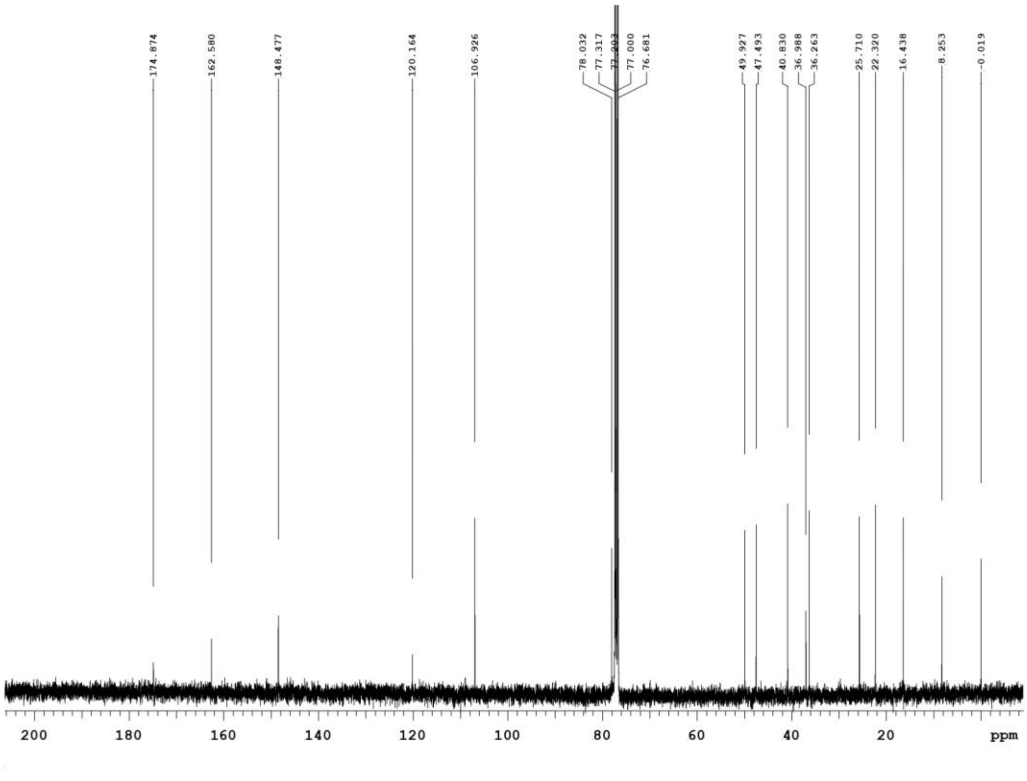

Figure S66. ^{13}C NMR spectrum (100 MHz) of compound **16** in $CDCl_3$

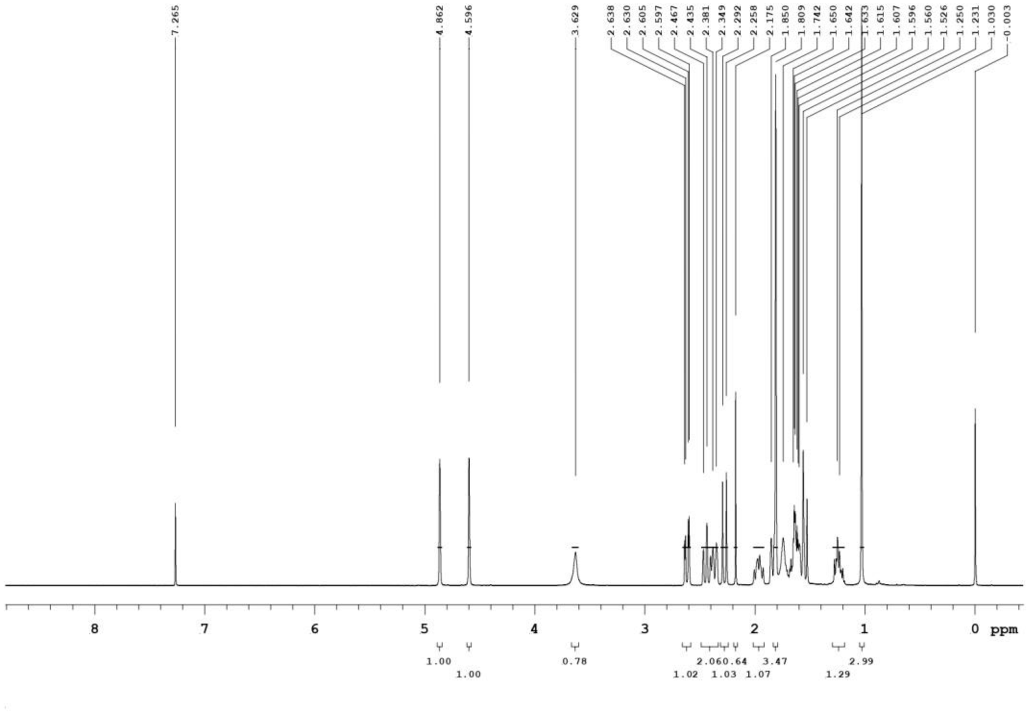

Figure S67. ^{1}H NMR spectrum (400MHz) of compound **17** in $CDCl_3$

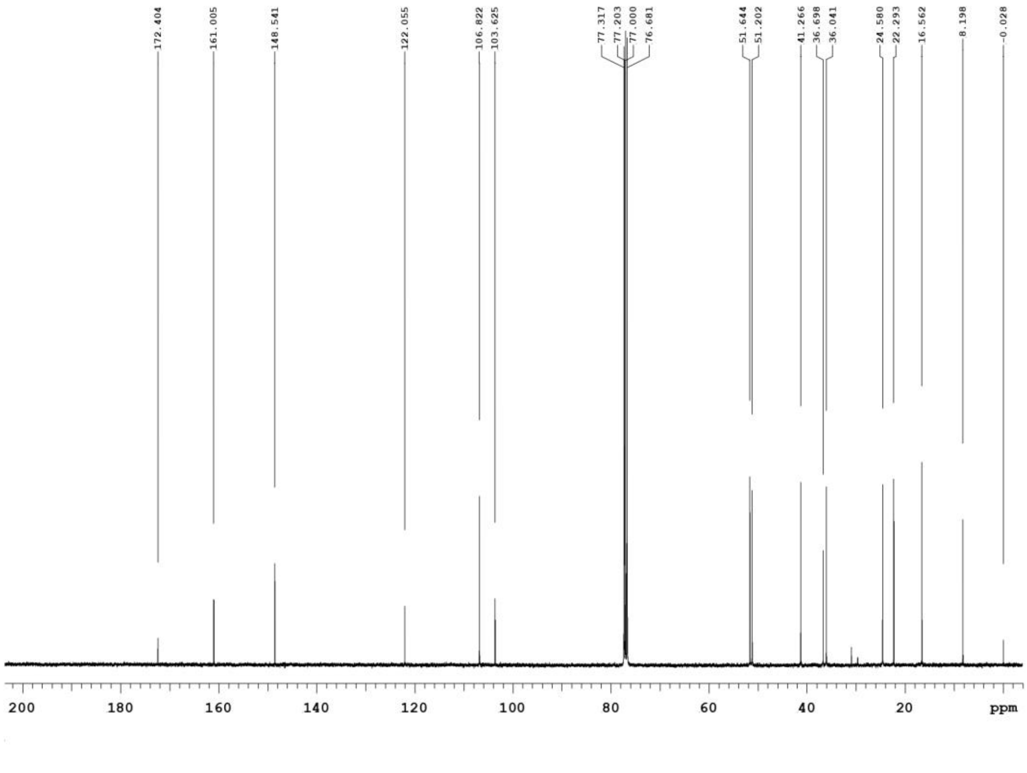

Figure S68. ^{13}C NMR spectrum (100 MHz) of compound **17** in $CDCl_3$

Table S1. The cytotoxicity data of compounds **1–16** and **1a**.

Cell lines IC_{50} (μg/mL)			
Compounds	HepG2	MDA-MB231	A549
**1**	> 20	> 20	> 20
**1a**	> 20	> 20	> 20
**2**	> 20	> 20	> 20
**3**	> 20	> 20	> 20
**4**	> 20	> 20	> 20
**5**	> 20	15.40	18.74
**6**	> 20	> 20	> 20
**7**	> 20	> 20	> 20
**8**	> 20	> 20	> 20
**9**	> 20	> 20	> 20
**10**	> 20	> 20	> 20
**11**	> 20	> 20	> 20
**12**	> 20	> 20	> 20
**13**	> 20	> 20	> 20
**14**	> 20	> 20	> 20
**15**	> 20	> 20	> 20
**16**	> 20	> 20	> 20
Doxorubicin	0.37	0.30	0.15

Table S2. Antibacterial activity (zone of inhibition in mm).

	Inhibition zone (mm)									
Compounds (25 μg/disk)	*B. s.*	*S. a.*	*S. e.*	*E. a.*	*E. c.*	*K. p.*	*S. m.*	*S. s.*	*S. t.*	*Y. e.*
**3**	–	–	–	–	–	–	–	–	–	–
**4**	–	–	–	–	–	–	–	–	–	–
**5**	–	–	6	–	–	5	–	–	–	–
**6**	–	–	–	–	–	–	–	–	–	–
**7**	–	–	–	–	–	–	–	–	–	–
**9**	–	–	–	–	–	9	–	–	–	–
**12**	–	–	–	–	–	–	–	–	–	–
Ampicillin	9	12	10	5	5	5	8	9	8	18

–: Inactive

Ampicillin: Positive control

B. s.: *Bacillus subtilis* (ATCC6051)

S. a.: *Staphylococcus aureus* (ATCC9144)

S. e.: *Salmonella enterica* (ATCC13076)

E. a.: *Enterobacter aerogenes* (ATCC13048)

E. c.: *Escherichia coli* (ATCC25922)

K. p.: *Klebsiella pneumoniae* (ATCC10031)

S. m.: *Serratia marcescens* (ATCC25419)

S. s.: *Shigella sonnei* (ATCC11060)

S. t.: *Salmonella typhimurium* (ATCC14028)

Y. e.: *Yersinia enterocolitica* (ATCC23715)

Table S3. Inhibitory effects of compounds **1–10**, **1a**, **14**, and **15** on TNF-*α* expression, PGE_2, and NO production in LPS induced dendritic cells.

Inh %[1]			
Compounds	TNF-*α*	PGE_2	NO
**1**	2.3 ± 2.0	2.9 ± 1.5	11.0 ± 2.4
**1a**	–0.1 ± 1.6	–0.8 ± 5.9	14.0 ± 1.5 *
**2**	7.3 ± 3.2	12.8 ± 3.5	19.6 ± 4.0 ***
**3**	8.0 ± 3.0	11.4 ± 2.1	33.8 ± 1.5 ***
**4**	3.8 ± 2.7	2.4 ± 2.1	34.9 ± 3.9 ***
**5**	0.8 ± 1.8	3.5 ± 4.2	16.0 ± 3.7 **
**6**	5.0 ± 2.9	–4.4 ± 0.4	14.6 ± 3.9 *
**7**	1.9 ± 5.1	–7.6 ± 2.8	3.7 ± 5.7
**8**	0.2 ± 0.5	–4.9 ± 3.2	10.2 ± 3.2
**9**	3.3 ± 1.7	1.2 ± 5.9	16.8 ± 4.7 **
**10**	–3.9 ± 1.1	–11.5 ± 4.6	11.9 ± 2.7
**14**	5.2 ± 1.8	–8.8 ± 6.3	24.8 ± 1.4***
**15**	23.6 ± 2.5 ***	21.2 ± 0.9 *	35.0 ± 3.7 ***
DEX[2]	85.6 ± 3.4 ****	–	73.4 ± 1.3 ****

1. Percentage of inhibition (Inh %) at the concentration 100 μM for **1–3**, **5**, **7**, **9**, **11**, **14** and **15** and 25 μM for **4**, **6**, and **8** compared with the control group (100 % for stimulated LPS alone). Results are presented as mean ± SEM. (n = 3). * $p < 0.05$, ** $p < 0.01$, *** $p < 0.001$, **** $p < 0.0001$.
2. Positive control: dexamethasone (DEX) at 100 μM.
